# Spectra–Structure Correlations in Isotopomers of Ethanol (CX_3_CX_2_OX; X = H, D): Combined Near-Infrared and Anharmonic Computational Study

**DOI:** 10.3390/molecules24112189

**Published:** 2019-06-11

**Authors:** Krzysztof B. Beć, Justyna Grabska, Christian W. Huck, Mirosław A. Czarnecki

**Affiliations:** 1 Institute of Analytical Chemistry and Radiochemistry, Leopold-Franzens University, Innrain 80/82, CCB-Center for Chemistry and Biomedicine, 6020 Innsbruck, Austria; Justyna.Grabska@uibk.ac.at (J.G.); Christian.W.Huck@uibk.ac.at (C.W.H.); 2 Faculty of Chemistry, University of Wrocław, F. Joliot-Curie 14, 50-383 Wrocław, Poland; miroslaw.czarnecki@chem.uni.wroc.pl

**Keywords:** near-infrared spectroscopy, ethanol, anharmonic quantum mechanical calculations, isotopic substitution, overtones, combinations bands

## Abstract

The effect of isotopic substitution on near-infrared (NIR) spectra has not been studied in detail. With an exception of few major bands, it is difficult to follow the spectral changes due to complexity of NIR spectra. Recent progress in anharmonic quantum mechanical calculations allows for accurate reconstruction of NIR spectra. Taking this opportunity, we carried out a systematic study of NIR spectra of six isotopomers of ethanol (CX_3_CX_2_OX; X = H, D). Besides, we calculated the theoretical spectra of two other isotopomers (CH_3_CD_2_OD and CD_3_CH_2_OD) for which the experimental spectra are not available. The anharmonic calculations were based on generalized vibrational second-order perturbation theory (GVPT2) at DFT and MP2 levels with several basis sets. We compared the accuracy and efficiency of various computational methods. It appears that the best results were obtained with B2PLYP-GD3BJ/def2-TZVP//CPCM approach. Our simulations included the first and second overtones, as well as binary and ternary combinations bands. This way, we reliably reproduced even minor bands in the spectra of diluted samples (0.1 M in CCl_4_). On this basis, the effect of isotopic substitution on NIR spectra of ethanol was accurately reproduced and comprehensively explained.

## 1. Introduction

Near-infrared (NIR) spectra are appreciably more complex and difficult for interpretation than IR or Raman spectra [1,2,3,4,5,6]. This results from a large number of strongly overlapping overtones and combination bands, numerous resonances between different modes and anharmonicity of vibrations [1,2,3,4,5,6]. Interpretation of vibrational bands has been aided by studies of a series of similar compounds (including isotopomers), or by reconstruction of the spectra by using quantum mechanical calculations [5]. The former way may provide highly speculative assignments, while the latter method has limitations that prevent their common use in NIR spectroscopy. From the point of view of applied spectroscopy, there exists an essential difference in the applicability of quantum mechanical calculation of mid-infrared (MIR) [7,8,9] and NIR spectra. A simplistic and computationally inexpensive harmonic approximation fails to predict the overtones and combination modes [10]. Because of a considerable computational cost of anharmonic calculations, simulations of NIR spectra are rare. Nevertheless, in the literature one can find examples of application of different approaches used for calculation of overtones and combination bands, including variational approaches, which are expensive but useful in selected cases [11,12]. However, the studies using a vibrational self-consistent field (VSCF) approach [13,14] and its refined variants—e.g., PT2-VSCF—are more common [15,16,17]. Recently, a number of theoretical reconstructions of NIR spectra by means of efficient vibrational second-order perturbation (VPT2) method have been reported [18]; e.g., carboxylic acids [19], fatty acids [20,21,22], aminoacids [23], nucleobases [24], nitriles [25], azines [26], phenols [27,28], and alcohols [29,30]. Considerable efforts have been undertaken in order to develop anharmonic approaches applicable to even larger molecular systems [31,32,33,34]. On the other hand, meticulous probing of vibrational potential capable of yielding nearly-exact results is also available [35,36,37,38]. Recent advances in this field include the development of multi-dimensional approaches that provide complete information on mode couplings in linear triatomic molecules [39].

The isotopic effect appears to be helpful for the analysis of NIR spectra [10]. By shifting a part of overlapped contributions, one can reduce their complexity and reveal individual bands. Time-resolved NIR spectroscopy of deuterated alcohols has also been successfully used for elucidating the diffusion coefficients [40]. In our previous work, the effect of isotopic substitution on NIR spectra of methanol has been accurately reproduced by anharmonic calculations [41]. In particular, we were able to predict the vibrational contributions from non-uniformly substituted CX_3_OX (X = H, D) species, which are not available from the experiment [41]. Further studies on molecules more complex than methanol are still necessary. A reasonable progress in this field is expected by examination of ethanol and all its isotopomers [42,43]. Ethanol has eight major isotopomers resulting from deuteration (CX_3_CX_2_OX; X = H, D), as compared to four in methanol. Moreover, the internal rotation around C-O(H) bond leads to rotational isomerism (*gauche*, *trans*) in molecules of ethanol, which adds additional origin of spectral variability in NIR spectra [42,43].

To enable detailed examination of the impact of various effects on NIR spectra of ethanol isotopomers, at first it is necessary to perform reliable theoretical reproduction of NIR spectra for eight isotopomers of ethanol (CX_3_CX_2_OX; X = H, D). We are interested in accurate reproduction of subtle effects observed in NIR spectra. Therefore, a combination of several electronic methods underlying VPT2 vibrational analysis will be useful for establishing the best approach capable of reproducing fine features in NIR spectra. The determination of electronic structure underlying the geometry optimization and harmonic analysis will be based on Møller–Plesset second-order perturbation (MP2) and density functional (DFT) theories. The efficiency and accuracy of reproduction of NIR bands by MP2 and DFT with single-hybrid B3LYP and double-hybrid B2PLYP density functionals will be overviewed. MP2 and DFT calculations included basis sets of increasing quality (6-31G(d,p), SNST, def2-TZVP, and aug-cc-pVTZ). Moreover, the impact of solvent cavity model will be evaluated. The anharmonic vibrational analysis will be carried out by means of generalized VPT2 (GVPT2). In our previous studies on methanol, it has been demonstrated that the relative contributions from the second overtones and ternary combinations are different for various isotopomers [41]. This work will provide detailed information on contributions from different vibrational modes and the trends observed with increasing of the alkyl chain length in going from methanol to ethanol. In addition, we will elucidate the accuracy of prediction of the three quanta transitions in NIR spectra.

## 2. Results and Discussion

### 2.1. Accuracy of Reproduction of NIR Spectra by Selected Approaches

Anharmonic calculations are significantly more challenging compared to harmonic approximation [1,2,3,4]. This holds even for efficient anharmonic approaches based on VPT2 method. At the same time, the higher quanta transitions are more prone to inaccuracies than the fundamental ones [44]. An insufficient accuracy of the ground state geometry and potential energy surface may easily propagate into inaccuracy of prediction in VPT2 calculation step. Thus, the theoretical prediction of NIR spectra is usually a compromise between the cost and accuracy. Effects like isotopic substitution [41] and conformational isomerism [29,30] may further complicate the vibrational analysis of NIR spectra. 

One of the aims of this work was assessment of the efficiency of several combinations of the electronic theory methods and basis sets (Table 1). In addition, we examined the effect of the solvent model on the anharmonic vibrational energies. An efficient single-hybrid B3LYP functional is commonly used tool for spectroscopic studies [10]. Empirical correction for dispersion has been introduced to overcome one of the major weaknesses of DFT method [45]. In some cases, this approach markedly improves the robustness of calculation of primary parameters (i.e., energy). Therefore, recent literature suggests employing empirical correction for dispersion in DFT calculations [46]. Our previous studies have shown that, even for small and isolated molecules, this correction is advantageous in spectra modeling [29]. For molecules in solution an approximation of the solvent cavity often improves the quality of the simulated NIR spectra [28]. However, in the present work this advantage is less important (Table 1). We observed an improvement of RMSE from 45 to 35 cm^−1^ for CH_3_CH_2_OH, and from 27 to 24 cm^−1^ for CH_3_CH_2_OD. However, considering small additional cost of CPCM (ca. 10% of total CPU time in the case of GVPT2//B3LYP-GD3BJ/6-31G(d,p) calculations), it is advisable to include this correction step in the calculations.

**Figure 1:**
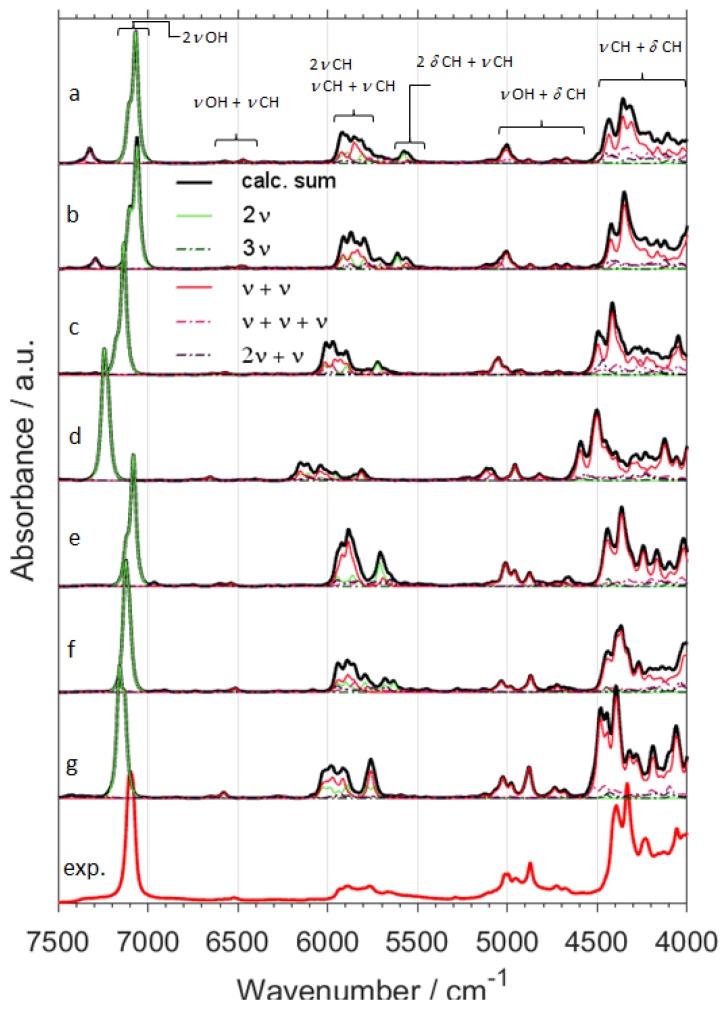
NIR spectra of CH_3_CH_2_OH calculated with GVPT2 method at different levels of electronic theory; (**a**) B3LYP-GD3BJ/6-31G(d,p); (**b**) B3LYP-GD3BJ/6-31G(d,p)//CPCM; (**c**) B2PLYP-GD3BJ/6-31G(d,p)//CPCM; (**d**) MP2/6-31G(d,p)//CPCM; (**e**) B3LYP-GD3BJ/SNST//CPCM; (**f**) B2PLYP-GD3BJ/def2-TZVP//CPCM; (**g**) MP2/aug-cc-pVTZ//CPCM; (**exp.**) Experimental spectrum of CH_3_CH_2_OH in CCl_4_ (0.1 M).

**Figure 2:**
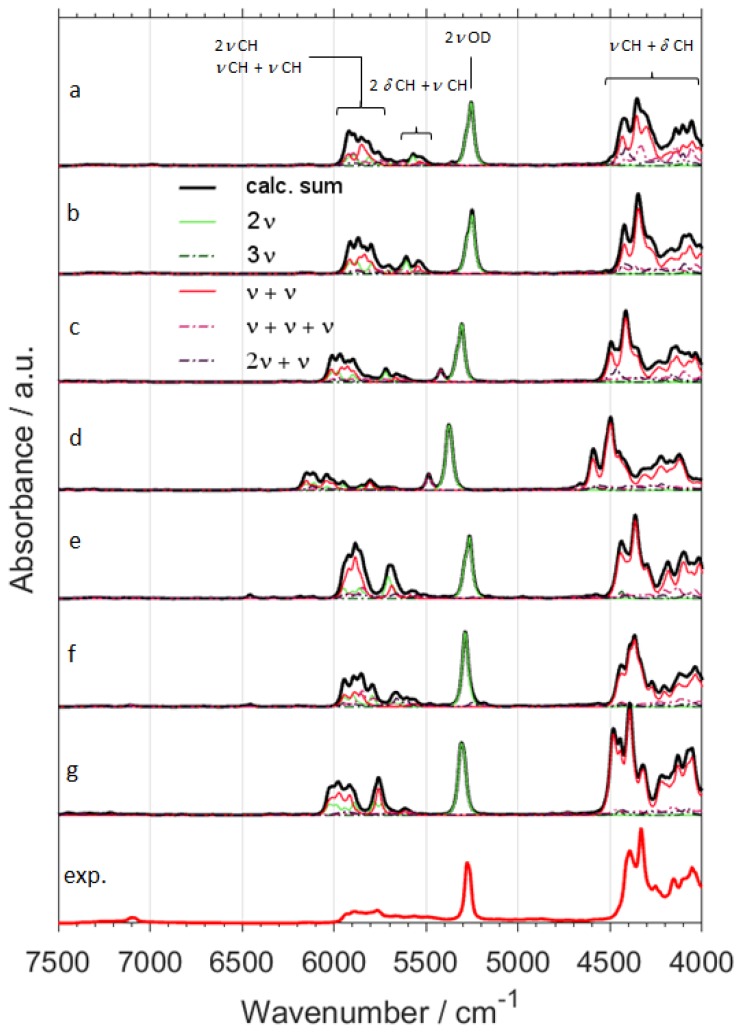
NIR spectra of CH_3_CH_2_OD calculated with GVPT2 method at different levels of electronic theory; (**a**) B3LYP-GD3BJ/6-31G(d,p); (**b**) B3LYP-GD3BJ/6-31G(d,p)//CPCM; (**c**) B2PLYP-GD3BJ/6-31G(d,p)//CPCM; (**d**) MP2/6-31G(d,p)//CPCM; (**e**) B3LYP-GD3BJ/SNST//CPCM; (**f**) B2PLYP-GD3BJ/def2-TZVP//CPCM; (**g**) MP2/aug-cc-pVTZ//CPCM; (**exp.**) Experimental spectrum of CH_3_CH_2_OD in CCl_4_ (0.1 M).

**Figure 3:**
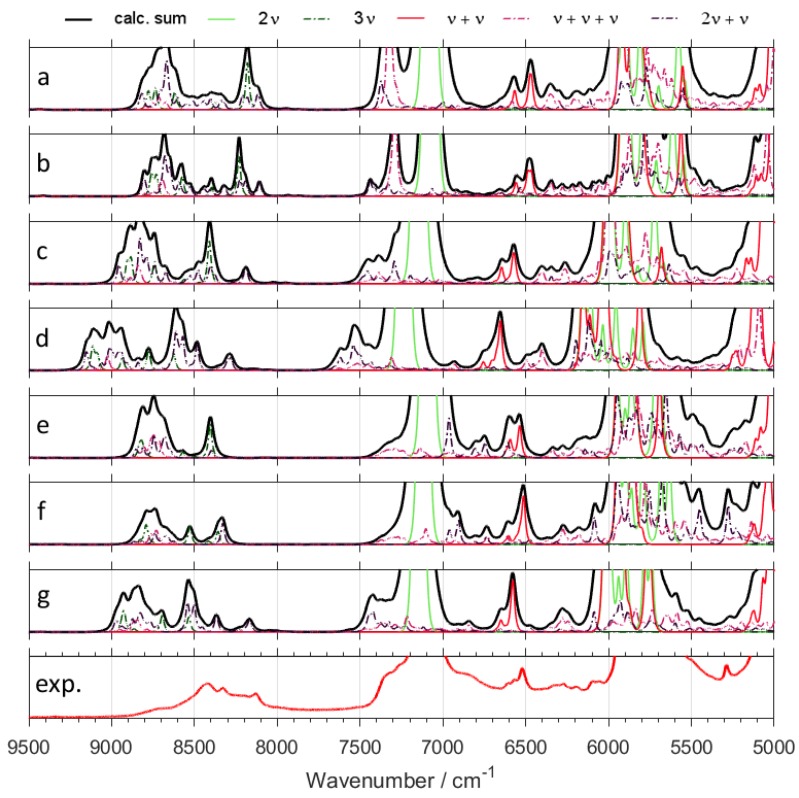
Contributions from minor bands in NIR spectra of CH_3_CH_2_OH calculated with GVPT2 method at different levels of electronic theory; (**a**) B3LYP-GD3BJ/6-31G(d,p); (**b**) B3LYP-GD3BJ/6-31G(d,p)//CPCM; (**c**) B2PLYP-GD3BJ/6-31G(d,p)//CPCM; (**d**) MP2/6-31G(d,p)//CPCM; (**e**) B3LYP-GD3BJ/SNST//CPCM; (**f**) B2PLYP-GD3BJ/def2-TZVP//CPCM; (**g**) MP2/aug-cc-pVTZ//CPCM; (**exp.**) Experimental spectrum of CH_3_CH_2_OH in CCl_4_ (0.1 M).

**Figure 4:**
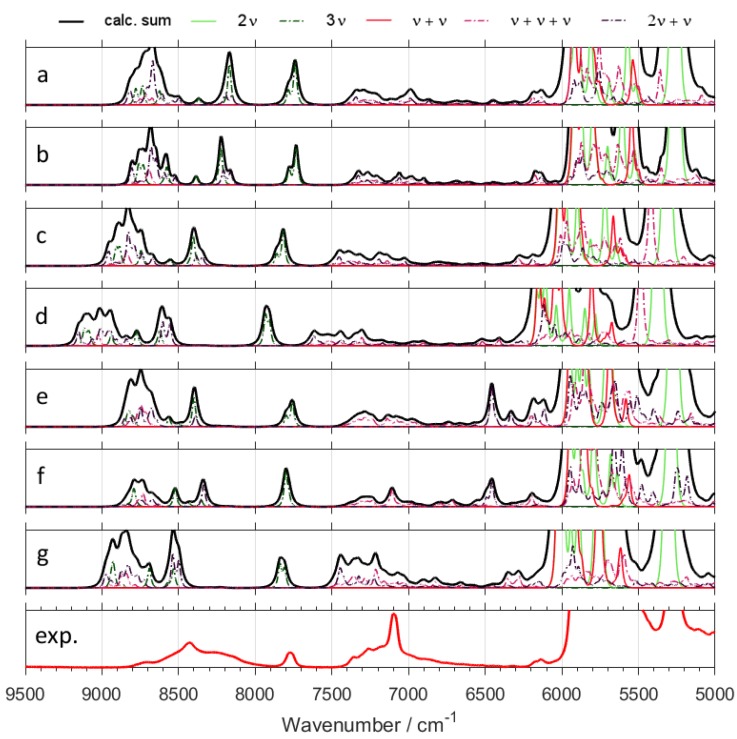
Contributions from minor bands in NIR spectra of CH_3_CH_2_OD calculated with GVPT2 method at different levels of electronic theory; (**a**) B3LYP-GD3BJ/6-31G(d,p); (**b**) B3LYP-GD3BJ/6-31G(d,p)//CPCM; (**c**) B2PLYP-GD3BJ/6-31G(d,p)//CPCM; (**d**) MP2/6-31G(d,p)//CPCM; (**e**) B3LYP-GD3BJ/SNST//CPCM; (**f**) B2PLYP-GD3BJ/def2-TZVP//CPCM; (**g**) MP2/aug-cc-pVTZ//CPCM; (**exp.**) Experimental spectrum of CH_3_CH_2_OD in CCl_4_ (0.1 M).

Switching from B3LYP to B2PLYP density functional with a small basis set (6-31G(d,p)) leads to an increase in the RMSE value (Table 1). However, B2PLYP method overestimated the band positions in a systematic manner. In contrast, B3LYP approach provides irregular results. Some of the band positions (*δ*CH) are blue-shifted, while the others (*ν*OX and *ν*CX; X = H, D) are red-shifted. Thus, more uniform band shift from B2PLYP method (Figure 1 and Figure 2) resulted in better interpretability of NIR spectra as compared with B3LYP results. It has a peculiar effect in NIR spectra of aliphatic alcohols, as it reduces RMSE of NIR band positions, particularly for the *ν*OX + *δ*CH, and *ν*CX + *δ*CH combination bands. However, this apparent gain does not improve the true interpretability of the spectra. It is likely that simulated NIR spectra of larger molecules may suffer even more due to binary combinations involving the stretching and deformation of the C-H and O-H vibrations. Similar inconsistency of B3LYP as compared to B2PLYP has been noted before [29,41]. Due to electron correlation being computed effectively at the MP2 level, it is commonly accepted that B2PLYP functional requires larger basis sets. B2PLYP coupled with large def2-TZVP basis set noticeably improves the quality of simulated NIR spectra (Figure 1 and Figure 2). This improvement is particularly evident in the reproduction of minor bands originating from the three quanta transitions (Figure 3 and Figure 4). This effect is nicely illustrated by reduction of RMSE from 72–83 cm^−1^ for B2PLYP/6-31G(d,p) to 18–19 cm^−1^ for B2PLYP/def2-TZVP. SNST basis set, less complex than def2-TZVP but still of triple-*ζ* quality, leads to worse results. An exception was observed for the prominent doublet from the *ν*CH combination bands (at ca. 4400 cm^−1^), where B2PLYP/SNST calculations reproduced the peak shapes more resembling the experimental ones. However, the position of this doublet was also overestimated by this method. Hence, B2PLYP/def2-TZVP method appears to be the better tool for reliable reconstruction of NIR spectra. A similar conclusion was obtained for butyl alcohols [30].

In contrast, MP2 method does not appear to be particularly useful for anharmonic calculations of NIR spectra of ethanol isotopomers due to significant redshift of the *ν*CX frequencies (Figure 1 and Figure 2). It is an interesting observation, as the tendency of MP2 to describe incorrectly repulsive forces is known in the literature [47]. In this work, however, an insufficient basis set (6-31G(d,p)) may strongly deviate the results. Here, MP2 method seems to be more sensitive to the effect of a small basis set than DFT-B2PLYP method. As expected, an application of a larger basis set, e.g., aug-cc-pVTZ, improves the accuracy of calculations. However, these results are not as good as those obtained from B2PLYP/def2-TZVP method. Moreover, this improvement is accompanied by a substantial increase of computing time (by ca. 4 times). Nevertheless, selected spectral ranges (*ν*OH + *δ*CH ≈ 5050–4800 cm^−1^ and *ν*CH + *δ*CH doublet ≈ 4400 cm^−1^) were better reproduced by MP2/aug-cc-pVTZ computations. Therefore, MP2 approach may be recommended as a reference method in selected cases. Our results demonstrate that different computational methods achieve different accuracy for particular regions of NIR spectra, and these regions do not overlap. Thus, comparison of the spectra simulated by different methods (e.g., resulting from VPT2 calculations at DFT or MP2 levels) appears to be the best way for reliable interpretation of the experimental spectra.

Particular attention should be paid to 2*ν*OH/OD band, which is the most characteristic peak for alcohols and the other important compounds like, e.g., phenols [27], terpenes [28], and polyphenols [48]. This peak is very sensitive to the chemical environment and inter- and intra-molecular interactions, and is frequently used for studies of the structure and physicochemical properties [49,50,51,52,53]. Hence, its proper theoretical reproduction is of essential importance. As shown in Figure 1, Figure 2, Figure 3 and Figure 4, and Figures S1–S6 in SM, most of the methods did not reproduce correctly the shape of this band. The experimental band from 2*ν*OH/OD vibration reveals a slight asymmetry. This asymmetry is a result of convolution of two components due to *trans* and *gauche* conformers. The lower-frequency *gauche* component has also the lower intensity. Among isotopomers of ethanol, this feature was reproduced correctly only by B2PLYP/def2-TZVP method. MP2/6-31G(d,p) and MP2/aug-cc-pVTZ methods predicted correct shape of the 2*ν*OH/OD peak only for CH_3_CD_2_OH and CD_3_CD_2_OD. As can be seen (Table 1), the peak position was overestimated by all used methods, but B2PLYP calculations give the best agreement. 

In comparison with other modes, large amplitude motions (LAMs)—e.g., torsion modes and hindered rotations—are more difficult for accurate description in harmonic approximation and also by anharmonic approaches that probe the potential curve relatively shallow (e.g., VPT2) [54]. We did not find any evidence that these low-frequency modes influence NIR bands directly (i.e., their overtone and combination modes do not appear in NIR region). However, a NIR spectrum provides some insights on LAMs as well. In our case, the shape of the 2*ν*OH/OD band is an indirect probe of the accuracy of prediction of the low-frequency modes. The shape of this band results from two components due to *gauche* and *trans* rotational conformers. Unreliable theoretical abundances of these forms would result in biased relative intensities of the 2*ν*OH/OD components (Table S1). Gibbs free energies may be affected by erroneous LAMs, which would propagate into incorrect relative abundances of *gauche* and *trans* conformers. Because it is an isolated band of strong intensity, the simulated 2*ν*OH/OD may be used to assess the reliability of prediction of LAMs and the related Gibbs free energies. This kind of error would manifest itself as a distorted shape of simulated 2*ν*OH/OD band. Above effect can be seen for some of the methods used in this study, e.g., for B3LYP (B3LYP-GD3BJ/6-31G(d,p); B3LYP-GD3BJ/6-31G(d,p)//CPCM; B3LYP-GD3BJ/SNST//CPCM;) and B2PLYP coupled with an insufficient basis set (B2PLYP-GD3BJ/6-31G(d,p)//CPCM;). However, the methods which yielded the most accurate spectra in the other regions (B2PLYP/def2-TZVP; MP2/6-31G(d,p); MP2/aug-cc-pVTZ) also reproduced 2*ν*OH/OD peak accurately (Figure 1 and Figure 2). Therefore, we conclude that the LAMs of ethanol and its derivatives were determined adequately by MP2 method. B2PLYP method also provides correct results, but it is more sensitive to the selection of a basis set. On the other hand, B3LYP tends to falsify the Gibbs free energies corrected by anharmonic ZPE. Further studies are needed to determine, whether this effect occurs because of an unreliable description of LAMs. On the other hand, inaccuracy of the 2*ν*OH/OD frequencies prediction by VPT2 may also be considered as another contributing factor, as we have evidenced such occurrence in the case of the conformers of cyclohexanol [27]. Note that B3LYP functional coupled with a relatively simple basis set yields reasonable reproduction of NIR spectra and correctly predicts the effects of isotopic substitution at a relatively modest computational expense (Figure 1, Figure 2, Figure 3 and Figure 4 and Figures S1–S6 in SM). However, a tendency to over- and underestimate the position and intensity of some bands may be unfavorable for the reliable interpretation of theoretical NIR spectra. For exploration of more subtle effects, B2PLYP functional seems to be more suitable. In the present study of isotopic substitution and the other effects (e.g., rotational isomerism) on NIR spectra of ethanol, we used B2PLYP/def2-TZVP method with additions of GD3BJ and CPCM.

### 2.2. Origins of NIR Bands of CX_3_CX_2_OX (X = H, D)

**Figure 5:**
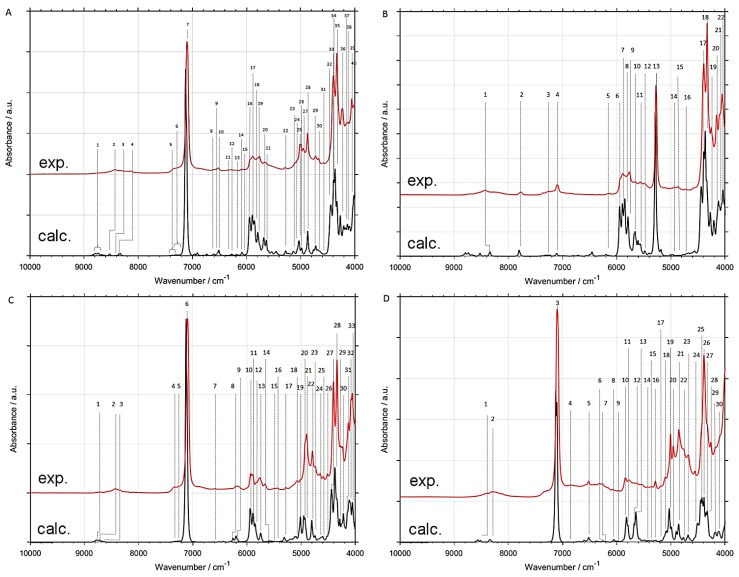
Band assignments in NIR spectra of deuterated ethanols based on GVPT2//B2PLYP-GD3BJ/def2-TZVP//CPCM calculations. (**A**) CH_3_CH_2_OH; (**B**) CH_3_CH_2_OD; (**C**) CH_3_CD_2_OH; (**D**) CD_3_CH_2_OH. Band numbering corresponds to that presented in Table 2, Table 3, Table 4 and Table 5.

**Figure 6:**
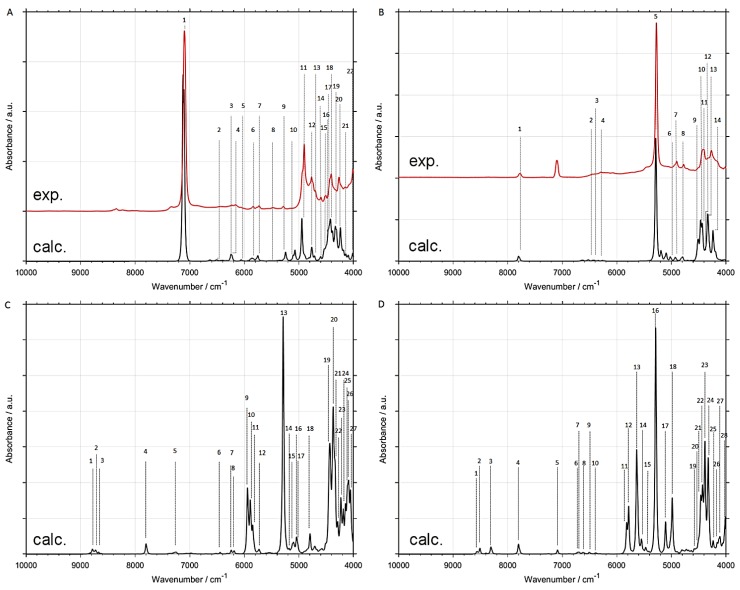
Band assignments in NIR spectra of deuterated ethanols based on GVPT2//B2PLYP-GD3BJ/def2-TZVP//CPCM calculations. (**A**) CD_3_CD_2_OH; (**B**) CD_3_CD_2_OD; (**C**) CH_3_CD_2_OD; (**D**) CD_3_CH_2_OD. Band numbering corresponds to that presented in Table 6, Table 7, Table 8 and Table 9.

The simulations of NIR spectra of ethanol isotopomers in CCl_4_ solutions by GVPT2 anharmonic method at B2PLYP-GD3BJ/def2-TZVP//CPCM level accurately reproduced most of the experimental bands (Figure 5 and Figure 6). On this basis, we performed detailed and reliable band assignments (Table 2, Table 3, Table 4, Table 5, Table 6, Table 7, Table 8 and Table 9). The consistency of these assignments was positively verified by comparison with the experimental spectra of six isotopomers. High accuracy of simulations allows to analyze the theoretical spectra of CH_3_CD_2_OD and CD_3_CH_2_OD (Figure 6C,D and Table 8 and Table 9) which are not available commercially. All assignments were supported by an analysis of the potential energy distributions (PEDs; Tables S2–S9).

NIR spectra of ethanol isotopomers mainly consist of the combinations of stretching and bending OX and CX (X = H, D) modes (Figure 1, Figure 2, Figure 3, Figure 4, Figure 5 and Figure 6 and Table 2, Table 3, Table 4, Table 5, Table 6, Table 7, Table 8 and Table 9). The region below 5500 cm^−1^ for CH_3_CH_2_OH is almost entirely contributed by the combination bands, while absorption from the overtones dominates above 5500 cm^−1^. NIR spectrum of ethanol may be roughly divided into four regions, but only two of them contain meaningful contributions from overtones. These regions are contributed mainly by vibrations from: (1) 2*ν*OX; (2) 2*ν*CX and *ν*CX + *ν*CX; (3) *ν*OX + *δ*CX; (4) *ν*CX + *δ*CX; (X = H, D). The other combination bands like *ν*OX + *ν*CX, and 2*δ*CX + *ν*CX have low intensity. Isotopic substitution introduces significant band shift, strongly affecting the appearance of NIR spectra (Figure 5 and Figure 6 and Figures S1–S6). It should be noted, that the region of 5700–5400 cm^−1^ for ethanols containing CH_3_ and CH_2_ groups is strongly affected by the anharmonic effects. This effect is well seen for CH_3_CH_2_OH (Figure 1) and CH_3_CH_2_OD (Figure 2). The most meaningful contributions in this region originate from *ν*CH + *ν*CH (*ν*_as_CH_2_ + *ν*_s_CH_2_), 2*δ*CH + *ν*CH, and 2*ν*_as_CH_2_ vibrations as well.

One can notice the overestimated intensities of the 2*ν*CH and *ν*CH + *ν*CH bands appearing in the 6000–5500 cm^−1^ region (Figure 5 and Figure 6 and Figures S1–S6). The magnitude of this effect varies between the different methods; however, it is present in all cases. A similar overestimation we have observed for butyl alcohols [30]. At present, we are unable to explain the reasons for these overestimations. Unexpectedly, B2PLYP functional (regardless of basis set; 6-31G(d,p), SNST, and def2-TZVP yielded similar results) significantly overestimates the frequencies of 2*δ*CH + *δ*CH transitions, shifting them to the 5500 cm^−1^ region. In contrast, the most of other transitions in NIR region is accurately reproduced by this approach. In the case of the 2*δ*CH + *δ*CH modes, large positive anharmonic constants appeared in GVPT2 vibrational analysis. Consequently, positions of the corresponding bands were predicted far from a simple combination of the harmonic frequencies. This shift has not been observed for the remaining approaches. Presently, the reason of this behavior is not clear. Because of very low intensity of 2*δ*CH + *δ*CH bands, these erroneous predictions do not provide meaningful contributions to NIR spectra. However, this occurrence demonstrates the need for using more than one method during examination of the fine spectral effects.

The deuteration of the OH group leads to a noticeable shift of the *ν*OD + *δ*CH band. In contrast, the other bands do not shift meaningfully, as can be easily seen from comparison of CH_3_CH_2_OH and CH_3_CH_2_OD spectra (Figure 5A,B). In particular, the absorption from the *ν*CH + *δ*CH in the 4600–4000 cm^−1^ region remains unaffected. This region can be used to monitor the isotopic substitutions of the CH_3_ and CH_2_ groups, as it leads to highly specific spectral changes. Obviously, simultaneous deuteration of both groups implies more significant changes. However, the most interesting effects result from the selective substitution of one of these groups. The presence of the CH_3_ group gives rise to a prominent doublet near 4395 and 4330 cm^−1^. This doublet has a complex structure resulting from overlapping of the contributions from the CH_2_ (Figure S7 in Supplementary Material), leading to a broadening of the high-frequency wing of the doublet. As expected, this contribution is not present in the spectrum of CH_3_CD_2_OH (Figure S7B). On the other hand, the isotopic substitution of the CH_3_ reveals a part of the overlapping contributions, as observed more clearly in the second derivative spectrum of CD_3_CH_2_OH (Figure S7C). This effect is well seen in the calculated spectra (Figure S7C).

The higher frequency NIR region (>7000 cm^−1^) is also very sensitive to the isotopic effect. A weak absorption from the higher order overtones and combination bands creates difficulties in the analysis of this region. The deuteration of the OH group significantly reduces the number of the bands as a result of red-shift of the combination bands. The simulated spectra confirmed the high isotopic purity of the samples, except of CD_3_CD_2_OD which shows the 2*ν*OH peak near 7100 cm^−1^ in the experimental spectrum (Figure 6B). This is in contrast to previously studied methanol, in which various non-uniform substitutions have been identified [41]. Contrary to -CX bonds, the H or D atoms in -OX bonds are labile, therefore, the OD group tends to exchange into the OH even by exposition of the deuterated alcohol to air. Since this band has a high absorptivity, therefore even small impurities due to the OH appear in NIR spectrum as a clear band at 7100 cm^−1^. In contrast, no -CH bands are observed in the spectrum of CD_3_CD_2_OD (Figure 6B). NIR spectroscopy is particularly sensitive and selective for the isotopic effect, although the theoretical calculations are necessary for proper spectra interpretation. The spectral manifestations of the OH group in OD derivatives are obscured by ternary combinations from the CH vibrations that appear in the same region. For example, the *δ*_as_’CH_3_ + *ν*_s_CH_2_ + *ν*_as_CH_2_ bands in CH_3_CH_2_OD (Figure 5B), and *δ*_sciss_CH_2_ + *ν*_s_CH_2_ + *ν*_as_CH_2_ bands in CD_3_CH_2_OD (Figure 6D) are observed. 

One can speculate that the isotopic substitution and conformational isomerism lead to convoluted spectral changes. This phenomenon will be a subject of our next paper (in preparation).

Another insight, which becomes possible only through theoretical simulation of NIR spectra, is estimation of the relative contributions from different kinds of vibrational transitions (Table 10). As compared with methanol [41], ethanol offers better opportunity to analyze these contributions, because of higher number of isotopomers and more complex NIR spectra. The effect of various kinds of isotopic substitution of the CH_3_, CH_2_, and OH groups on NIR spectra may be elucidated. In the 10,000–4000 cm^−1^ region two quanta transitions, first overtones (2*ν*_x_) and binary combinations (*ν*_x_ + *ν*_y_), are the most meaningful components of the spectra. In particular, binary combinations from the CH_3_ group have significant contribution—e.g., for CH_3_CH_2_OH they are responsible for 47% of NIR intensity—while upon deuteration of the CH_3_ group this contribution decreases to 32.6%. An even more pronounced effect is observed for OD derivatives, the analogous values for CH_3_CH_2_OD and CD_3_CH_2_OD are 51.2% and 35.5%, respectively. Simultaneously, the isotopic substitution of the methyl group increases the relative intensity of the first overtones, while the intensity of the second overtones remains insignificant. As expected, the importance of the second overtones increases in the upper NIR region (10,000–7500 cm^−1^). Interestingly, this trend is not observed for the ternary combinations (*ν*_x_ + *ν*_y_ + *ν*_z_ and 2*ν*_x_ + *ν*_y_), although for OD derivatives the 2*ν*_x_ + *ν*_y_ contribution increases and the *ν*_x_ + *ν*_y_ + *ν*_z_ contribution decreases upon deuteration of the CH_3_ group. The isotopic substitution of the CH_2_ group provides similar changes, but is noticeably less significant.

As can be seen (Table 10), the region above 7500 cm^−1^ is contributed only by three and higher quanta transitions. Therefore, in this region the effect of isotopic substitution is even more visible. The deuteration of the CH_3_ group increases the contributions from the second overtones at the expense of *ν*_x_ + *ν*_y_ + *ν*_z_ combinations, while the contributions from 2*ν*_x_ + *ν*_y_ remain similar. Interestingly, NIR spectrum of CD_3_CD_2_OD above 7500 cm^−1^ includes the second overtones only.

These observations remain in agreement with our previous findings on methanol isotopomers [41]. However, the contributions from the three quanta transitions are more important for ethanol. For CH_3_CH_2_OH these transitions involve 25.9% of total intensity (10,000–4000 cm^−1^), while for CH_3_OH this value was found to be 19.2%. The difference between CD_3_OD and CD_3_CD_2_OD is even larger (23.5% vs. 36.7%). 

## 3. Experimental and Computational Methods

### 3.1. Materials and Spectroscopic Measurements

In Table 11 are collected the details on the samples used in this work. The experimental spectrum of CH_3_CH_2_OH was taken from our previous work [29]. All samples were used as received, while solvent (CCl_4_) was distilled and additionally dried using freshly activated molecular sieves (Aldrich, 4A). All ethanols were measured in CCl_4_ solution (0.1 mol dm^−3^). NIR spectra were recorded on Thermo Scientific Nicolet iS50 spectrometer using InGaAs detector, with a resolution of 2 cm^−1^ (128 scans), in a quartz cells (Hellma QX, Hellma Optik GmbH, Jena, Germany) of 100 mm thicknesses at 298 K (25 °C). 

### 3.2. Computational Procedures

Our calculations were based on density functional theory (DFT) with double-hybrid B2PLYP density functional [55] (unfrozen core) coupled with Karlsruhe triple-*ζ* valence with polarization (def2-TZVP) [56] basis set. Grimme’s third formulation of empirical correction for dispersion with Becke-Johnson damping (GD3BJ) was applied [57]. To better reflect solvation of molecules, CCl_4_ cavity in solvent reaction field (SCRF) [58] was included at conductor-like polarizable continuum (CPCM) [59] level. Very tight criteria for geometry optimization and 10^−10^ convergence criterion in SCF procedure were set. Electron integrals and solving coupled perturbed Hartree-Fock (CPHF) equations were calculated over a superfine grid. The selected method provided good reproduction of NIR spectra of various molecules in CCl_4_ solution [19,29,30].

We carried out the anharmonic vibrational analysis at generalized vibrational second-order perturbation theory (GVPT2) [60,61] level. In this approach, the anharmonic frequencies and intensities of the vibrational transitions up to three quanta were obtained. This allows to simulate fundamental, first and second overtones, as well as binary and ternary combination bands. Quantum mechanical calculations were carried out with Gaussian 16 (A.03) [62]. One of the major features implemented in GVPT2 approach is the automatic treatment of tight vibrational degenerations, i.e., resonances [63]. In this work the search for resonances included Fermi (i.e., 1-2) of type I (*ω*_i_ ≈ 2*ω*_j_) and type II (*ω*_i_ ≈ *ω*_j_ + *ω*_k_), and Darling–Dennison (i.e., 2-2, 1-1, and 1-3) resonances. All possible resonant terms within search thresholds were included in the variational treatment. The resonance search thresholds (respectively, maximum frequency difference and minimum difference PT2 vs. variational treatment; in (cm^−1^)) were: 200 and 1 (for the search of 1–2 resonances), 100 and 10 (for 2-2, 1-1, and 1-3).

To display the simulated spectra we applied a four-parameter Cauchy–Gauss (Lorentz–Gauss) product function [20]. The theoretical bands were modelled with *a*_2_ and *a*_4_ parameters equal to 0.055 and 0.015, resulting with full-width at half-height (FWHH) of 25 cm^−1^. Exception was made for better agreement with the weaker and broader experimental bands, which are presented in Figure 3 and Figure 4. In this case the values were 0.075, 0.015, and 35 cm^−1^, respectively. The final theoretical spectra were obtained by combining the spectra of *trans* and *gauche* conformers, mixed in accordance with the calculated abundances of each form [64]. The relative abundances of the *gauche* (*n*_g_) and *trans* (*n*_t_) conformers were determined as following equation [65].

$$\frac{n_{g}}{n_{t}}=\frac{A_{t}}{A_{g}}e^{\frac{-\Delta G^{298}}{RT}}$$

where Gibbs free energy (Δ*G*) corresponds to the value calculated at 298 K corrected by anharmonic (VPT2) zero-point energy (ZPE); *A_t_* and *A_g_* are the degeneracy prefactors of the Boltzmann term for the *gauche* (1) and *trans* (2) conformers.

The band assignments were aided by calculations of potential energy distributions (PEDs). PEDs were obtained with Gar2Ped software [66], using natural internal coordinate system defined in accordance with Pulay [67]. The numerical analysis of the theoretical results and the processing of the experimental spectra were performed with MATLAB R2016b (The Math Works Inc.) [68]. 

## 4. Conclusions

Isotopic substitution leads to much higher variability in NIR spectra as compared with IR spectra, due to significant contribution from the combination bands. The pattern of OH/OD, CH_3_/CD_3_, and CH_2_/CD_2_ groups in ethanol often leads to fine spectral changes, which may be monitored and explained in detail by anharmonic quantum mechanical simulations. Our studies were devoted to NIR spectra of eight isotopomers of ethanol (CX_3_CX_2_OX (X = H, D)) by using anharmonic GVPT2 vibrational analysis. The calculations were performed at several levels of electronic theory, including DFT and MP2 to find accurate and efficient theoretical approach for studies of isotopic effect in NIR spectra. Our results indicate that DFT approach using double-hybrid B2PLYP functional, coupled with def2-TZVP basis set, and supported by GD3BJ correction with CPCM solvent model yielded the best results. The theoretical spectra obtained by this approach enabled us to assign most of NIR bands, including two (2*ν*_x_ and *ν*_x_ + *ν*_y_) and three quanta (3*ν*_x_, *ν*_x_ + *ν*_y_ + *ν*_z_, and 2*ν*_x_ + *ν*_y_) transitions. Accuracy of these calculations permitted us to analyze theoretical NIR spectra of CH_3_CD_2_OD and CD_3_CH_2_OD for which the experimental spectra are not available. The effect of the isotopic substitution of the OH, CH_3_, and CH_2_ groups was satisfactory reproduced and explained. Moreover, the relative contributions of selected groups and kinds of transitions were elucidated and discussed. The contributions from the CH_3_ group appear to be more important than those from the CH_2_ group. The isotopic substitution in the CH_3_ group leads to the most prominent intensity changes in NIR spectra as compared to the changes due to the substitution of the other groups. The bands from the three quanta transitions are more important for isotopomers of ethanol than for derivatives of methanol. 

## Figures and Tables

**Table 1:** Positions of selected NIR bands (in cm^−1^) from GVPT2 anharmonic vibrational analysis in CH_3_CH_2_OH and CH_3_CH_2_OD at different levels of electronic theory and corresponding RMSE values.

Assignment	Exp.	Calculated													
		MP2/aVTZ + CPCM	Diff.	B2PLYP-GD3BJ/def2-TZVP + CPCM	Diff.	B2PLYP-GD3BJ/SNST + CPCM	Diff.	MP2/6-31G(d,p) + CPCM	Diff.	B2PLYP-GD3BJ/6-31G(d,p) + CPCM	Diff.	B3LYP-GD3BJ/6-31G(d,p) + CPCM	Diff.	B3LYP-GD3BJ/6-31G(d,p)	Diff.
	CH_3_CH_2_OH														
2*ν*OH	7099	7157	58	7125	26	7081	−18	7243	144	7134	35	7061	−38	7096	−3
*ν*_s_CH_2_ + *ν*OH	6520.4	6578	57.6	6513	−7.4	6536	15.6	6654	133.6	6576	55.6	6477	-43.4	6472	−48.4
2*δ*_as’_CH_3_ + *ν*OH	5886.1	5980	93.9	5889	2.9	5922	35.9	6111	224.9	5970	83.9	5871	−15.1	5892	5.9
2*ν*_as_CH_2_	5765.7	5897	131.3	5790	24.3	5864	98.3	5957	191.3	5898	132.3	5797	31.3	5812	46.3
2*ν*_s_CH_2_; 2*ν*_s_CH_3_ + *δ*_s_CH_3_	5665.1	5759	93.9	5681	15.9	5708	42.9	5812	146.9	5722	56.9	5611	−54.1	5768	102.9
[*δ*_wagg_CH_2_, *δ*_s_CH_3_] + *ν*OH	5013.8	5026	12.2	5029	15.2	5012	−1.8	5114	100.2	5049	35.2	5040	26.2	5019	5.2
[*δ*_twist_CH_2_, *δ*_ip_COH, *δ*_wagg_CH_2_] + *ν*OH	4954.2	4978	23.8	4979	24.8	4959	4.8	5003	48.8	5009	54.8	5008	53.8	5003	48.8
*δ*_ip_COH + *ν*OH	4873	4881	8	4868	−5	4877	4	4958	85	4926	53	4874	1	4883	10
*δ*_s_CH_3_ + *ν*_as’_CH_3_	4394.8	4448	53.2	4366	−28.8	4443	48.2	4592	197.2	4473	78.2	4424	29.2	4436	41.2
[*δ*_oop_COH, *τ*CC] + *ν*OH	4333.5	4395	61.5	4331	−2.5	4364	30.5	4502	168.5	4416	82.5	4353	19.5	4357	23.5
		**RMSE**	70.0	**RMSE**	18.1	**RMSE**	40.8	**RMSE**	153.1	**RMSE**	72.2	**RMSE**	35.0	**RMSE**	44.6
	CH_3_CH_2_OD														
2*ν*_as_CH_3_	5885.2	5978	92.8	5895	9.8	5921	35.8	6114	228.8	5967	81.8	5871	−14.2	5894	8.8
2*ν*_as_CH_2_	5765.6	5917	151.4	5788	22.4	5862	96.4	6007	241.4	5896	130.4	5799	33.4	5815	49.4
2*ν*OD	5277.1	5312	34.9	5289	11.9	5265	−12.1	5378	100.9	5306	28.9	5250	−27.1	5255	−22.1
*δ*_sciss_CH_2_ + *ν*_as_CH_2_	4393.7	4445	51.3	4397	3.3	4439	45.3	4592	198.3	4493	99.3	4422	28.3	4424	30.3
[*δ*_s_CH_3_, *δ*_wagg_CH_2_] + *ν*_as_CH_3_	4331.8	4392	60.2	4364	32.2	4364	32.2	4500	168.2	4415	83.2	4349	17.2	4355	23.2
[*δ*_rock_CH_2_, *δ*_rock_CH_3_] + *ν*_s_CH_2_	4054.1	4057	2.9	4037	−17.1	4020	−34.1	4122	67.9	4036	−18.1	4068	13.9	4058	3.9
		**RMSE**	80.6	**RMSE**	18.6	**RMSE**	50.0	**RMSE**	179.4	**RMSE**	83.3	**RMSE**	23.6	**RMSE**	27.3

**Table 2:** Band assignments in NIR spectra of CH_3_CH_2_OH based on GVPT2//B2PLYP-GD3BJ/def2-TZVP//CPCM calculations. Band numbering corresponds to that presented in Figure 5A.

Peak Number	*ν*_Exp_	*ν*_Calc_	Assignment (Major Contribution)
1	8718.0	8739	2*ν*_as_CH_2_ + *ν*_as_CH_3_
2	8430.0	8526	3*ν*_as_CH_2_
3	8329.0	8416	3*ν*_s_CH_2_
4	8131.0	8329	2*ν*_s_CH_2_ + *ν*_as_CH_2_
5	7400–7300	7400–7300	*δ*_s_CH_3_ + *ν*_as_CH_3_ + *ν*_as’_CH_3_ [*δ*_as’_CH_3_, *δ*_as_CH_3_] + *ν*_as_CH_3_ + *ν*_as_’CH_3_ *δ*_sciss_CH_2_ + *ν*_as_CH_2_ + *ν*_as_’CH_3_ [*δ*_rock_CH_2,_ *δ*_rock_CH_3_] + *ν*_as_CH_2_ + *ν*OH
6	7300–7200	7300–7200	*δ*_twist_CH_2_ + *ν*_as_CH_3_ + *ν*_as_’CH_3_ *δ*_s_CH_3_ + *ν*_s_CH_3_ + *ν*_as_’CH_3_ [*δ*_as_CH_3,_ *δ*_as_’CH_3_] + *ν*_s_CH_3_ + *ν*_as_CH_3_ [*δ*_as_CH_3,_ *δ*_as_’CH_3_] + *ν*_s_CH_3_ + *ν*_as_’CH_3_
7	7099.0	7125	2*ν*OH
8	6610.0	6609	*ν*_s_CH_3_ + *ν*OH
9	6565.0	6540	2*δ*_as_CH_3_ + *ν*OH
10	6520.4	6513	*ν*_s_CH_2_ + *ν*OH
11	6331.0	6314	2*δ*_twist_CH_2_ + *ν*OH
12	6271.8	6275	*δ*_ip_COH + *δ*_wagg_CH_2_ + *ν*OH
13	6193.0	6178	[*τ*CC, *δ*_oop_COH] + *ν*_as_CH_3_ + *ν*_as_’CH_3_
14	6063.0	6085	2*δ*_ip_COH + *ν*OH
15	6051.0	6021	[*ν*CC, *δ*_ip_COH] + *δ*_twist_CH_2_ + *ν*OH
16	5936.0	5948	2*ν*_as_’CH_3,_ *ν*_as_CH_3_ + *ν*_as_’CH_3_
17	5886.1	5889	2*δ*_as_’CH_3_ + *ν*OH
18	5809.0	5846	[*δ*_as_CH_3,_ *δ*_as_’CH_3_] + *δ*_sciss_CH_2_ + *ν*_as_CH_2_
19	5765.7	5790	2*ν*_as_CH_2_
20	5665.1	5681	2*ν*_s_CH_2_; 2*ν*_s_CH_3_ + *δ*_s_CH_3_
21	5634.0	5632	*δ*_ip_COH + *δ*_wagg_CH_2_ + *ν*_s_CH_3_
22	5287.6	5277	*δ*_ip_OH + *δ*CCO +*ν*OH
23	5111.0	5128	*δ*_sciss_CH_2_+ *ν*OH
24	5071.0	5118	[*δ*_as_CH_3,_ *δ*_as_’CH_3_] + *ν*OH
25	5013.8	5029	[*δ*_wagg_CH_2,_ *δ*_s_CH_3_] + *ν*OH
26	4996.2		
27	4954.2	4979	[*δ*_twist_CH_2,_ *δ*_ip_COH, *δ*_wagg_CH_2_] + *ν*OH
28	4873.0	4868	*δ*_ip_COH + *ν*OH
29	4724.3	4763	*δ*_s_CH_3_ + 2[*δ*_as_CH_3,_ *δ*_as_’CH_3_]
30	4677.0	4726	[*ν*CO, *δ*_rock_’CH_3_] + *ν*OH
31	4582.9	4648	[*δ*_oop_COH, *τ*CC] + *δ*_as_’CH_3_ + *ν*_s_CH_3_
32	4454.0	4450	3*δ*_sciss_CH_2_
33	4409.0	4396	*δ*_sciss_CH_2_ + *ν*_as_CH_2_
34	4394.8	4366	*δ*_s_CH_3_ + *ν*_as_’CH_3_
35	4333.5	4331	[*δ*_oop_COH, *τ*CC] + *ν*OH
36	4232.6	4269	*δ*_twist_CH_2_ + *ν*_as_’CH_3_
37	4162.0	4177	*δ*_ip_COH + *ν*_s_CH_2_
38	4131.7	4137	*δ*_twist_CH_2_ + *ν*_s_CH_2_
39	4057.4	4020	[*δ*_rock_CH_2,_ *δ*_rock_CH_3_] + *ν*_s_CH_2_
40	4024.0	3997	[*ν*CO, *δ*_rock_’CH_3_] + *ν*_as_CH_2_

**Table 3:** Band assignments in NIR spectra of CH_3_CH_2_OD based on GVPT2//B2PLYP-GD3BJ/def2-TZVP//CPCM calculations. Band numbering corresponds to that presented in Figure 5B.

Peak Number	*ν*_exp_	*ν*_calc_	Assignment (Major Contribution)
1	8428.0	8334	2*ν*_s_CH_2_ + *ν*_as_CH_2_
2	7777.5	7796	3*ν*OD
3	7260.0	7227	*δ*_twist_CH_2_ + *ν*_as_CH_3_ + *ν*_as_’CH_3_
4	7099.1	7112	*δ*_as_’CH_3_ + *ν*_s_CH_2_ + *ν*_as_CH_2_
5	6133.4	6200	*τ*CC + *ν*_as_CH_3_ + *ν*_as_’CH_3_
6	5935.0	5946	2*ν*_as_’CH_3_
7	5885.2	5895	2*ν*_as_CH_3_
8	5850.0	5847	[*δ*_as_CH_3,_ *δ*_as_’CH_3_] + *δ*_sciss_CH_2_ + *ν*_as_CH_2_
9	5765.6	5788	2*ν*_as_CH_2_
10	5665.7	5669	2*δ*_wagg_CH_2_ + *ν*_s_CH_2_
11	5564.3	5559	*ν*OD + *ν*_s_CH_2_
12	5494.3	5498	*ν*_as_CH_2_ + *δ*_twist_CH_2_ + *δ*_wagg_CH_2_
13	5277.1	5289	2*ν*OD
14	4947.0	4963	[*δ*_rock_CH_2,_ *δ*_rock_CH_3_] + *δ*_twist_CH_2_ + *ν*_s_CH_2_
15	4873.0	4846	[*δ*_rock_CH_2,_ *δ*_rock_CH_3_] + [*δ*_rock_CH_2,_ *δ*_rock_CH_3_] + *ν*_s_CH_2_
16	4720.8	4717	[*ν*CO, *δ*_rock_’CH_3,_ *δ*_ip_COD] + [*δ*_rock_’CH_3,_ *δ*_ip_COD, *δ*_sciss_CH_2_CO] +*ν*OD
17	4393.7	4397	*δ*_sciss_CH_2_ + *ν*_as_CH_2_
18	4331.8	4364	[*δ*_s_CH_3,_ *δ*_wagg_CH_2_] +*ν*_as_CH_3_
19	4253.4	4275	*δ*_twist_CH_2_ + *ν*_as_’CH_3_
20	4155.1	4127	[*δ*_rock_’CH_3,_ *δ*_ip_COD, *δ*_sciss_CH_2_CO] +*ν*_as_CH_3_
21	4105.0	4063	[*δ*_rock_’CH_3,_ *δ*_ip_COD, *δ*_sciss_CH_2_CO] + *ν*_s_CH_3_
22	4054.1	4037	[*δ*_rock_CH_2_, *δ*_rock_CH_3_] + *δ*_s_CH_2_

**Table 4:** Band assignments in NIR spectra of CH_3_CD_2_OH based on GVPT2//B2PLYP-GD3BJ/def2-TZVP//CPCM calculations. Band numbering corresponds to that presented in Figure 5C.

Peak Number	*ν*_exp_	*ν*_calc_	Assignment (Major Contribution)
1	8717.0	8788	3*ν*_as_CH_3_
2	8434.1	8728	*ν*_s_CH_3_ + *ν*_as_CH_3_ + *ν*_as_’CH_3_
3	8337.0	8665	2*ν*_s_CH_3_ + *ν*_as_CH_3_
4	7345.0	7324	[*δ*_rock_CD_2,_ *δ*_twist_ CD_2_] + *ν*_as_’CH_3_ + *ν*OH
5	7251.0	7255	[*δ*_as_CH_3,_ *δ*_as_’CH_3_] + *ν*_s_CH_3_ + *ν*_as_CH_3_
6	7098.2	7126	2*ν*OH
7	6590.4	6618	*ν*_as_’CH_3_ + *ν*OH
8	6205.8	6257	2*δ*_ip_COH + *ν*OH
9	6158.0	6198	2*δ*_ip_COH + *ν*OH
10	5929.9	5943	2*ν*_as_CH_3_
11	5895.4	5892	2*ν*_as_’CH_3_
12	5828.0	5844	*ν*_s_CH_3_ + *ν*_as_’CH_3_
13	5763.8	5744	*ν*_s_CD_2_ + *ν*OH
14	5669.1	5595	*ν*_s_CH_3_ + 2*ν*_as_CH_3_
15	5449.0	5496	*δ*_rock_CH_3_ + *δ*_s_CH_3_ + *ν*_as_’CH_3_
16	5439.4	5449	*δ*_sciss_CD_2_CO + 2*ν*_as_CH_3_
17	5282.0	5309	*ν*_as_CD_2_ + 2[*δ*_as_CH_3,_ *δ*_as_’CH_3_]
18	5070.1	5094	*ν*_s_CD_2_ + *ν*_as_CH_3_
19	5017.0	5018	[*τ*CC, *δ*_oop_ COH] + 2[*δ*_as_CH_3,_ *δ*_as_’CH_3_]
20	4927.0	4954	*δ*_ip_COH + *ν*OH
21	4898.3	4930	*δ*_ip_COH + *ν*OH
22	4792.1	4806	[*ν*CO, *δ*_wagg_CD_2_] + *ν*OH
23	4737.2	4744	*δ*_sciss_CD_2_ + *ν*OH
24	4650.2	4641	[*ν*CC, *δ*_rock_’CH_3_] + *ν*OH
25	4591.7	4600	[*ν*CO, *δ*_wagg_CD_2_] + *ν*OH
26	4513.3	4525	*δ*_ip_COH + *ν*OH
27	4404.6	4437	[*δ*_as_CH_3,_ *δ*_as_’CH_3_] + *ν*_as_’CH_3_
28	4338.0	4373	*δ*_s_CH_3_ + *ν*_as_’CH_3_
29	4275.7	4285	*δ*_ip_COH + *ν*_as_CH_3_
30	4238.1	4218	*ν*_s_CD_2_ + *ν*_as_CD_2_
31	4129.6	4147	[*ν*CO, *δ*_wagg_CD_2_] + *ν*_as_’CH_3_
32	4079.7	4117	*δ*_rock_CH_3_ + *ν*_as_CH_3_
33	4056.2	4052	*δ*_rock_CH_3_ + *ν*_s_CH_3,_ *δ*_sciss_CD_2_CO + *ν*OH

**Table 5:** Band assignments in NIR spectra of CD_3_CH_2_OH based on GVPT2//B2PLYP-GD3BJ/def2-TZVP//CPCM calculations. Band numbering corresponds to that presented in Figure 5D.

Peak Number	*ν*_exp_	*ν*_calc_	Assignment (Major Contribution)
1	8405.0	8511	3*ν*_as_CH_2_
2	8307.6	8323	2*ν*_s_CH_2_ + *ν*_as_CH_2_
3	7098.5	7124 (*t*) 7100 (*g*)	2*ν*OH
4	6841.1	6897	[*ν*CO, *δ*_as_’CD_3_] + *ν*_s_CH_2_ + *ν*_as_CH_2_
5	6517.6	6510	*ν*_s_CH_2_ + *ν*OH
6	6324.0	6261	[*δ*_ip_COH, *δ*_twist_CH_2_] + *δ*_wagg_CH_2_ + *ν*OH
7	6268.0	6111	[*δ*_rock_’CD_3,_ *ν*CC] + *ν*_as_’CD_3_ + *ν*OH
8	6070.4	6059	2[*δ*_ip_COH, *δ*_twist_CH_2_] + *ν*OH
9	5966.3	5966	[*δ*_s_CD_3,_ *ν*CC] + *δ*_wagg_CH_2_ + *ν*OH
10	5839.1	5825	2*ν*_as_CH_2_
11	5772.1	5793	2*ν*_as_CH_2_
12	5628.0	5686	2*ν*_s_CH_2_
13	5533.0	5641	2*ν*_s_CH_2_
14	5427.9	5419	2*δ*_twist_CH_2_ + *ν*_as_CH_2_
15	5358.0	5367	[*ν*CO, *δ*_as_’CD_3_] + *ν*_s_CD_3_ + *ν*_as_’CD_3_
16	5286.8	5287	*δ*_s_CD_3_ + *δ*_twist_CH_2_ + *ν*_as_CH_2_
17	5190.0	5188	2[*δ*_s_CD_3,_ *ν*CC] + *ν*_s_CH_2_
18	5102.7	5084	*δ*_oop_COH + 2*δ*_wagg_CH_2_
19	5007.1	5028	*δ*_wagg_CH_2_ + *ν*OH
20	4955.8	4987	[*δ*_twist_CH_2,_ *δ*_ip_COH, *δ*_wagg_CH_2_] + *ν*OH
21	4853.8	4853	[*δ*_ip_COH, *δ*_twist_CH_2_] + *ν*OH
22	4764.7	4768	[*δ*_s_CD_3,_ *ν*CC] + *ν*OH
23	4676.7	4676	*ν*CO + *ν*OH
24	4558.4	4511	*δ*_as_’CD_3_ + [*δ*_twist_CH_2,_ *δ*_ip_COH] + *ν*_as_’CD_3_
25	4443.0	4429	*δ*_sciss_CH_2_ + *ν*_as_CH_2_
26	4390.5	4390	*δ*_sciss_CH_2_ + *ν*_as_CH_2_
27	4329.0	4356	*δ*_sciss_CH_2_ + *ν*_s_CH_2_
28	4263.8	4332	*δ*_sciss_CH_2_ + *ν*_s_CH_2_
29	4174.6	4180	2[*δ*_twist_CH_2_, *δ*_ip_COH, *δ*_wagg_CH_2_] + *δ*_sciss_CH_2_
30	4100.0	4107	*τ*CC + *δ*_oop_COH + *ν*OH

**Table 6:** Band assignments in NIR spectra of CD_3_CD_2_OH based on GVPT2//B2PLYP-GD3BJ/def2-TZVP//CPCM calculations. Band numbering corresponds to that presented in Figure 6A.

Peak Number	*ν*_exp_	*ν*_calc_	Assignment (Major Contribution)
1	7099.0	7126 (*t*) 7102 (*g*)	2*ν*OH
2	6444.0	6495	*ν*_s_CD_2_ + [*ν*_as_CD_2_, *ν*_as_CD_3_] + [*ν*_as_’CD_3_, *ν*_as_CD_2_]
3	6232.1	6244	2*δ*_ip_COH +*ν*OH
4	6162.9	6224	2[*ν*CC, *δ*_wagg_CD_2_] + *ν*OH
5	6063.0	6059	[*δ*_sciss_CD_2,_ *ν*CO] + [*ν*CC, *δ*_wagg_CD_2_] + *ν*OH
6	5838.3	5861	[*ν*_as_CD_2_, *ν*_as_CD_3_] + *ν*OH
7	5732.3	5746	[*δ*_rock_CD_2,_ *δ*_rock_CD_3_] + [*δ*_s_CD_3,_ *δ*_wagg_CD_2_] + *ν*OH
8	5478.7	5488	[*ν*CC, *δ*_wagg_CD_2_] + *ν*_s_CD_3_ + [*ν*_as_CD_2_, *ν*_as_CD_3_]
9	5285.8	5247	*ν*_s_CD_3_ + *ν*_as_CD_2_ + *ν*CO
10	5160.0	5103	[*δ*_twist_CD_2,_ *δ*_rock_CD_3,_ *δ*_rock_CD_2_] + 2[*ν*CC, *δ*_wagg_CD_2_]
11	4903.7	4947	*δ*_ip_COH + *ν*OH
12	4769.2	4766	[*δ*_sciss_CD_2,_ *ν*CO] + *ν*OH
13	4701.4	4714	[*δ*_as_’CD_3_, *δ*_as_CD_3_] + *ν*OH
14	4598.4	4604	[*ν*CO, *δ*_wagg_CD_2_] + *ν*OH
15	4525.0	4539	[*δ*_twist_CD_2,_ *δ*_rock_CD_3,_ *δ*_rock_CD_2_] + *δ*_ip_COH + [*ν*_as_’CD_3,_ *ν*_as_CD_2_]
16	4506.9	4499	2*δ*_sciss_CD_2_CO + *ν*_as_CD_3_
17	4430.0	4447	[*ν*_as_CD_2_, *ν*_as_CD_3_] +*ν*_as_CD_3_, 2[*ν*_as_’CD_3_, *ν*_as_CD_2_]
18	4409.6	4420	[*ν*_as_CD_2_, *ν*_as_CD_3_] + [*ν*_as_’CD_3_, *ν*_as_CD_2_]
19	4332.0	4334	2*ν*_as_CD_2_
20	4267.4	4235	[*ν*_s_CD_2,_ *ν*_s_CD_3_] + *ν*_as_CD_2_
21	4156.8	4140	2*δ*_oop_COH +*ν*OH
22	4013.0	4012	*δ*_sciss_CD_2_CO +*ν*OH

**Table 7:** Band assignments in NIR spectra of CD_3_CD_2_OD based on GVPT2//B2PLYP-GD3BJ/def2-TZVP//CPCM calculations. Band numbering corresponds to that presented in Figure 6B.

Peak Number	*ν*Exp	*ν*Calc	Assignment (Major Contribution)
1	7771.0	7799	3*ν*OD
2	6468.0	6525	*ν*_s_CD_3_ + [*ν*_as_’CD_3,_ *ν*_as_CD_2_] + *ν*_as_CD_3_
3	6450.0	6447	3*ν*_as_CD_2_
4	6290.1	6267	2*ν*_s_CD_2_ + *ν*_as_CD_2_
5	5276.0	5289	2*ν*OD
6	4948.0	5020	[*ν*CO, *δ*_twist_CD_2_] + 2[*δ*_sciss_CD_2_, *ν*CO]
7	4902.2	4929	[*δ*_twist_CD_2,_ *δ*_rock_CD_2_] + 2[*δ*_sciss_CD_2_, *ν*CO]
8	4779.2	4795	*ν*_s_CD_2_ + *ν*OD
9	4509.0	4510	[*δ*_as_CD_3,_ *δ*_as_’CD_3_] + [*ν*CC, *δ*_wagg_CD_2_] +*ν*_as_CD_3_
10	4437.4	4465	2*ν*_as_’CD_3,_ *ν*_as_CD_3_ + *ν*_as_’CD_3_
11	4409.3	4434	2*ν*_as_CD_3,_ *ν*_as_CD_2_ + *ν*_as_’CD_3_
12	4325.2	4381	2[*δ*_sciss_CD_2_, *ν*CO] + *ν*_s_CD_3_
13	4269.5	4337	2*ν*_as_CD_2_
14	4170.2	4241	[*ν*_s_CD_2,_ *ν*_s_CD_3_] + *ν*_as_CD_2_

**Table 8:** Band assignments in NIR spectra of CH_3_CD_2_OD based on GVPT2//B2PLYP-GD3BJ/def2-TZVP//CPCM calculations. Band numbering corresponds to that presented Figure 6C.

Peak Number	*ν*Calc	Assignment (Major Contribution)
1	8781	3*ν*_as_’CH_3_
2	8722	*ν*_s_CH_3_ + *ν*_as_CH_3_ + *ν*_as_’CH_3_
3	8666	2*ν*_s_CH_3_ + *ν*_as_CH_3,_ 2*ν*_s_CH_3_ + *ν*_as_’CH_3_
4	7802	3*ν*OD
5	7257	[*δ*_as_CH_3,_ *δ*_as_’CH_3_] + *ν*_s_CH_3_ + *ν*_as_CH_3_
6	6445	3*ν*_as_CD_2_
7	6188	*τ*CC + *ν*_as_CH_3_ + *ν*_as_’CH_3_
8	5943	2*ν*_as_CH_3,_ *ν*_as_CH_3_ + *ν*_as_’CH_3_
9	5890	2[*δ*_as_’CH_3_, *δ*_as_CH_3_] + *ν*_as_’CH_3,_ 2*ν*_as_’CH_3_
10	5845	*ν*_s_CH_3_ + *ν*_as_CH_3_
11	5728	2*δ*_s_CH_3_ + *ν*_as_CH_3_
12	5288	2*ν*OD
13	5182	*ν*_as_CD_2_ + *ν*_as_’CH_3_
14	5120	*ν*_as_CD_2_ + *ν*_s_CH_3_
15	5097	*ν*_s_CD_2_ + *ν*_as_CH_3_
16	5046	*τ*CC + 2[*δ*_as_CH_3,_*δ*_as_’CH_3_]
17	4796	*δ*_oop_COD + 2[*δ*_as_’CH_3,_*δ*_as_CH_3_]
18	4434	*τ*CC + [*δ*_wagg_CD_2,_*ν*CC] + *ν*_as_’CH_3_
19	4372	*δ*_s_CH_3_ +*ν*_as_CH_3,_ *δ*_s_CH_3_ +*ν*_as_’CH_3_
20	4341	2*ν*_as_CD_2_
21	4289	*τ*CC + *δ*_sciss_CD_2_ + [*δ*_sciss_CD_2,_*ν*CO]
22	4233	*ν*_s_CD_2_ + *ν*_as_CD_2_
23	4190	2*ν*_s_CD_2_, [*δ*_wagg_CD_2,_ *ν*CC] + *ν*_as_CH_3_
24	4149	[*δ*_sciss_CD_2,_ *ν*CO] + *ν*_as_CH_3,_ [*δ*_sciss_CD_2,_ *ν*CO] +*ν*_as_’CH_3_
25	4113	*δ*_rock_CH_3_ + *ν*_as_’CH_3_
26	4089	[*δ*_sciss_CD_2,_ *ν*CO] + *ν*_s_CH_3_
27	4056	*δ*_rock_CH_3_ + *ν*_s_CH_3_

**Table 9:** Band assignments in NIR spectra of CD_3_CH_2_OD based on GVPT2//B2PLYP-GD3BJ/def2-TZVP//CPCM calculations. Band numbering corresponds to that presented Figure 6D.

Peak Number	*ν*_calc_	Assignment (Major Contribution)
1	8560	3*ν*_as_CH_2,_ 2*ν*_s_CH_2_ + *ν*_as_CH_2_
2	8508	3*ν*_as_CH_2_
3	8305	2*ν*_s_CH_2_ + *ν*_as_CH_2_
4	7802	3*ν*OD
5	7088	*δ*_sciss_CH_2_ + *ν*_s_CH_2_ + *ν*_as_CH_2_
6	6736	*δ*_rock_CH_2_ + *ν*_s_CH_2_ + *ν*_as_CH_2_
7	6706	[*ν*CO, *δ*_as_’CD_3_] + *ν*_s_CH_2_ + *ν*_as_CH_2_
8	6614	3*ν*_as_’CD_3_
9	6505	*ν*_s_CD_3_ +*ν*_as_CD_3_ +*ν*_as_’CD_3_
10	6382	2*ν*_s_CD_3_ + *ν*_as_CD_3,_ 2*ν*_s_CD_3_ + *ν*_as_’CD_3_
11	5821	[*δ*_oop_COD, *τ*CC] + *ν*_as_CH_2_ + *ν*_s_CH_2_
12	5785	2*ν*_as_CH_2,_ *δ*_wagg_CH_2_ + *δ*_sciss_CH_2_ + *ν*_as_CH_2_
13	5638	2*ν*_s_CH_2,_ *ν*_s_CH_2_ + *ν*_as_CH_2_
14	5547	*ν*OD + *ν*_s_CH_2_
15	5473	*δ*_rock_CH_2_ + *δ*_sciss_CH_2_ + *ν*_as_CH_2_
16	5288	2*ν*OD
17	5106	[*τ*CC, *δ*_oop_COD] + 2*δ*_wagg_CH_2_
18	4986	2[*ν*CC, *δ*_s_CD_3_] + *ν*OD
19	4585	*δ*_rock_’CD_3_ + 2*δ*_wagg_CH_2_
20	4503	[*τ*CC, *δ*_oop_COD] + *δ*_wagg_CH_2_ + *ν*_as_CH_2_
21	4459	2*ν*_as_CD_3_
22	4431	[*δ*_as_’CD_3_, *ν*CO] + [*ν*CC, *δ*_s_CD_3_] + *ν*_as_’CD_3_
23	4385	*δ*_sciss_CH_2_ + *ν*_as_CH_2_
24	4324	2*ν*_as_’CD_3,_ *δ*_sciss_CH_2_ + *ν*_s_CH_2_
25	4237	*δ*_wagg_CH_2_ + *ν*_s_CH_2_
26	4167	*δ*_twist_CH_2_ + *ν*_as_CH_2_
27	4112	*δ*_twist_CH_2_ + *ν*_s_CH_2_
28	4015	[*δ*_rock_CD_3,_ *δ*_rock_CH_2_] + *δ*_rock_CH_2_ + *ν*_as_’CD_3_

**Table 10:** Contributions (in %) from the first and second overtones as well as binary and ternary combinations into NIR spectra of ethanol isotopomers based on GVPT2//B2PLYP-GD3BJ/def2-TZVP//CPCM calculations. ^a^

	**10,000–4000 cm^−1^**				
	**2*ν*_x_**	**3*ν*_x_**	***ν*_x_ + *ν*_y_**	***ν*_x_ + *ν*_y_ + *ν*_z_**	**2*ν*_x_ + *ν*_y_**
CH_3_CH_2_OH	26.1	1.7	47.0	14.3	10.9
CH_3_CH_2_OD	18.0	2.2	51.2	17.4	11.1
CH_3_CD_2_OH	35.8	1.7	41.5	11.8	9.2
CD_3_CH_2_OH	40.9	1.2	32.6	15.1	10.1
CD_3_CD_2_OH	46.0	0.3	23.7	15.8	14.2
CD_3_CD_2_OD	43.1	2.0	19.2	17.8	17.9
CH_3_CD_2_OD	27.9	3.2	44.7	15.0	9.2
CD_3_CH_2_OD	36.0	2.5	35.5	10.8	15.2
	**10,000–7500 cm^−1^**				
	**2*ν*_x_**	**3*ν*_x_**	***ν*_x_ + *ν*_y_**	***ν*_x_ + *ν*_y_ + *ν*_z_**	**2*ν*_x_ + *ν*_y_**
CH_3_CH_2_OH	0.0	39.7	0.0	22.3	38.0
CH_3_CH_2_OD	0.0	55.5	0.0	15.4	29.1
CH_3_CD_2_OH	0.0	43.9	0.0	30.5	25.6
CD_3_CH_2_OH	0.0	66.9	0.0	1.4	31.7
CD_3_CD_2_OH	0.0	0.0	0.0	43.0	57.0
CD_3_CD_2_OD	0.0	100.0	0.0	0.0	0.0
CH_3_CD_2_OD	0.0	69.9	0.0	16.7	13.4
CD_3_CH_2_OD	0.0	76.3	0.0	0.5	23.2
	**7500–4000 cm^−1^**				
	**2*ν*_x_**	**3*ν*_x_**	***ν*_x_ + *ν*_y_**	***ν*_x_ + *ν*_y_ + *ν*_z_**	**2*ν*_x_ + *ν*_y_**
CH_3_CH_2_OH	26.5	1.2	47.6	14.2	10.5
CH_3_CH_2_OD	18.4	1.0	52.4	17.5	10.7
CH_3_CD_2_OH	36.0	1.4	41.8	11.7	9.1
CD_3_CH_2_OH	41.4	0.4	33.0	15.3	9.9
CD_3_CD_2_OH	46.0	0.3	23.7	15.8	14.2
CD_3_CD_2_OD	43.7	0.5	19.5	18.0	18.2
CH_3_CD_2_OD	28.4	2.0	45.5	15.0	9.1
CD_3_CH_2_OD	37.0	0.5	36.4	11.1	15.0

^a^ The comparison is based on integrated intensity (cm^−1^) summed over simulated bands, convoluted with the use of Cauchy−Gauss product function (details in the text) in relation to the total integrated intensity.

**Table 11:** Samples used in this study

	Sample	Purity	D Atom Content	Other Remarks
1	CH_3_CH_2_OD	99%	≥99.5%	
2	CH_3_CD_2_OH	99%	98%	
3	CD_3_CH_2_OH	99%	99%	
4	CD_3_CD_2_OH	99%	99.5%	
5	CD_3_CD_2_OD	>99%	≥99.5%	anhydrous
6	CCl_4_	>99%	-	

Samples were purchased from Sigma-Aldrich Chemie GmbH (Taufkirchen, Germany).

## Supplementary Materials

The following are available online, Figures S1–S35; Tables S1–S9.
