# Supplementary material for: Spectra–Structure Correlations in Isotopomers of Ethanol (CX3CX2OX; X = H, D): Combined Near-Infrared and Anharmonic Computational Study
Source: Molecules. 2019 Jun 11;24(11):2189. doi: 10.3390/molecules24112189 (PMC6600318; doi:10.3390/molecules24112189)
Supplement: Supplementary file 1 [file molecules-24-02189-s001.pdf]

molecules

MDPI

Supplementary Materials

Spectra–Structure Correlations in Isotopomers of Ethanol (CX_3CX_2OX; X = H, D): Combined Near-Infrared and Anharmonic Computational Study

Krzysztof B. Beć [1,2,*], Justyna Grabska [1], Christian W. Huck [1], and Mirosław A. Czarnecki [2]

[1] Institute of Analytical Chemistry and Radiochemistry, Leopold-Franzens University, Innrain 80/82, CCB-Center for Chemistry and Biomedicine, 6020-Innsbruck, Austria; Justyna.Grabska@ uibk.ac.at (J.G.); Christian.W.Huck@uibk.ac.at (C.W.H.)

[2] Faculty of Chemistry, University of Wrocław, F. Joliot-Curie 14, 50-383 Wrocław, Poland; miroslaw.czarnecki@chem.uni.wroc.pl

* Correspondence: Krzysztof.Bec@uibk.ac.at; Tel.: +43-512-507-5783

I. Figures

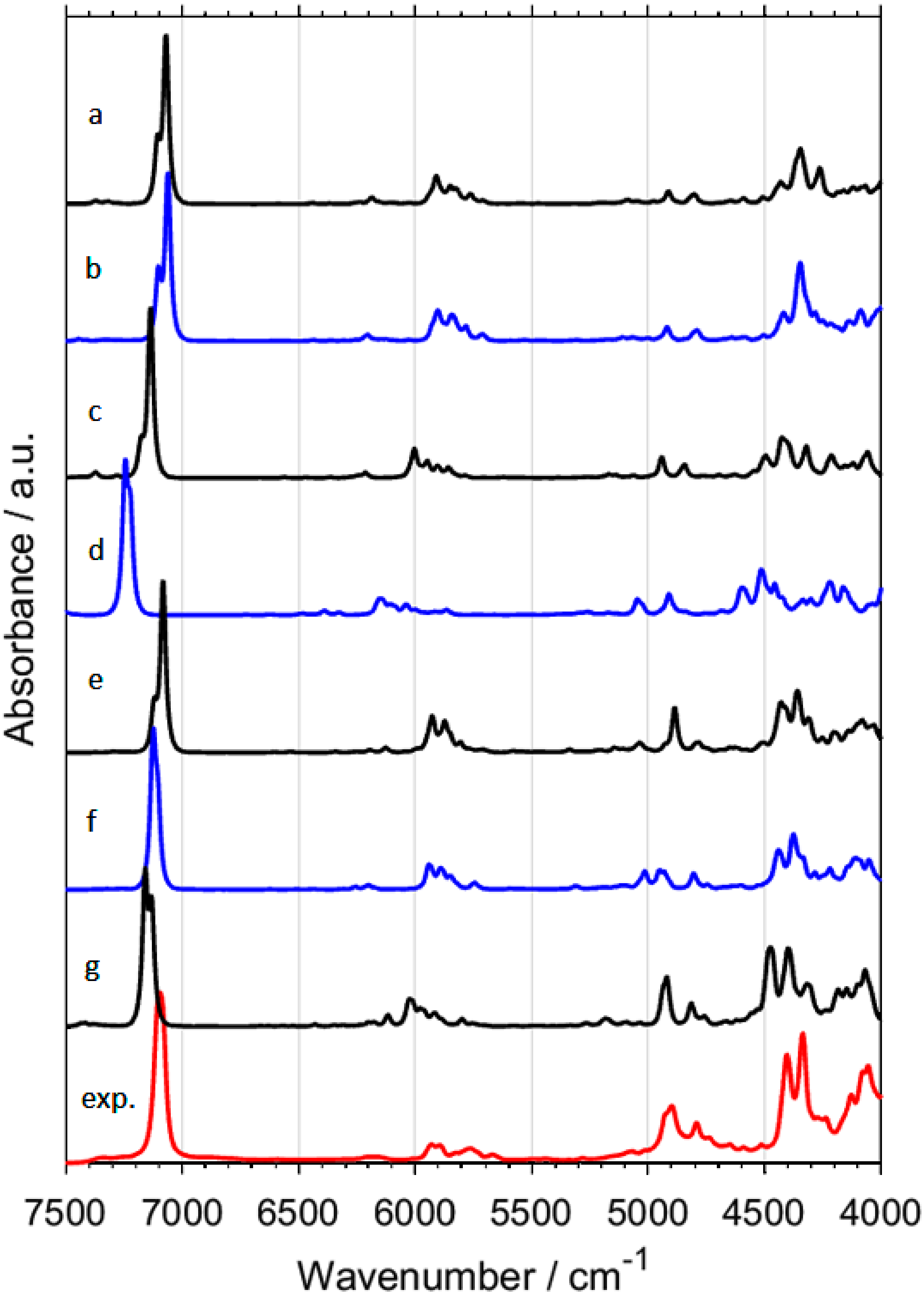

Figure S1. NIR spectra of CH_3CD_2OH calculated with GVPT2 method at different levels of electronic theory; (a) B3LYP-GD3BJ /6-31G(d,p); (b) B3LYP-GD3BJ /6-31G(d,p)//CPCM; (c) B2PLYP-GD3BJ/6-31G(d,p)//CPCM; (d) MP2/6-31G(d,p)//CPCM; (e) B3LYP-GD3BJ/SNST//CPCM; (f) B2PLYP-GD3BJ/def2-TZVP//CPCM; (g) MP2/aug-cc-pVTZ//CPCM. Experimental spectrum (exp.) of CH_3CD_2OH in CCl_4 (0.1 M).

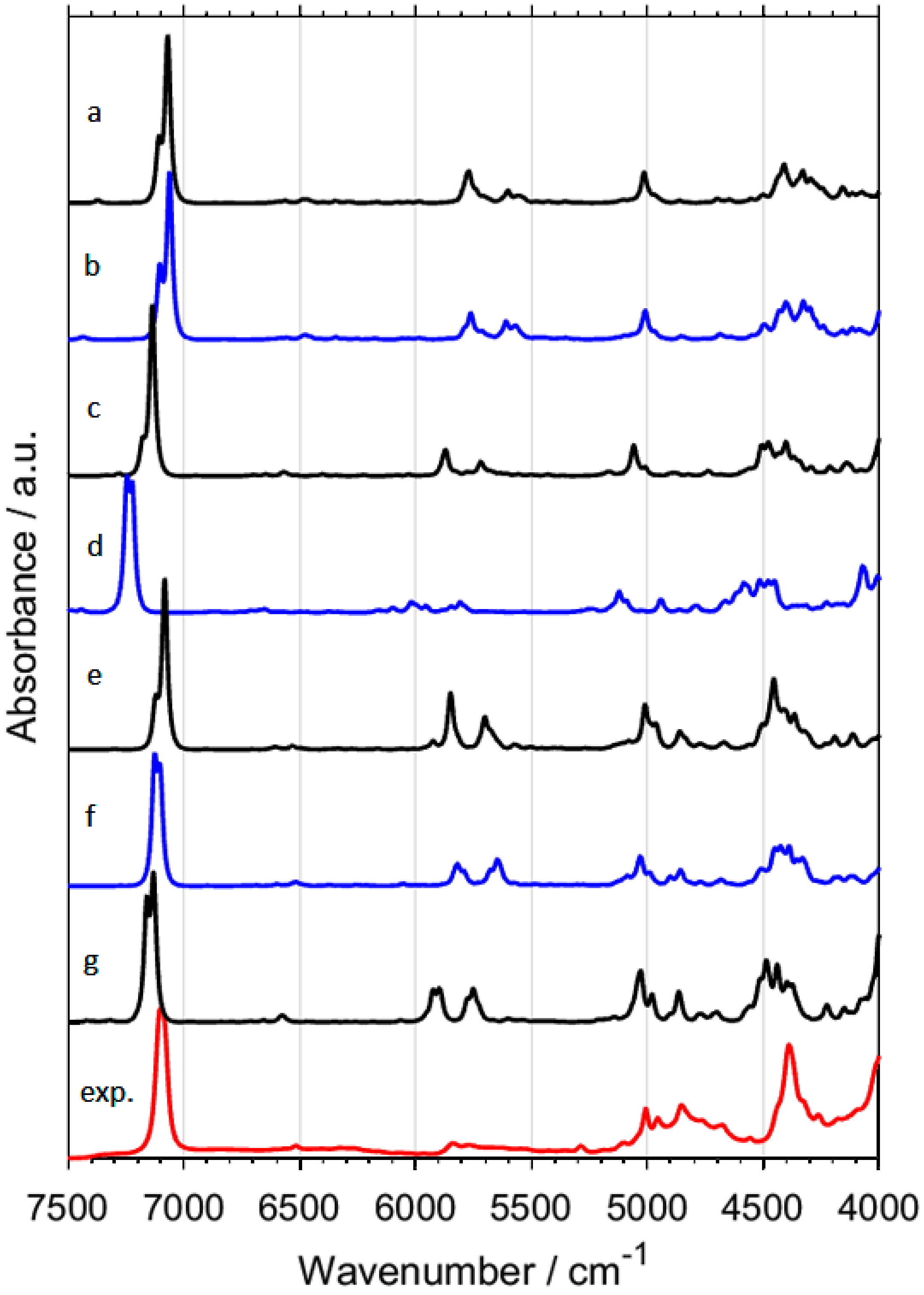

Figure S2. **NIR spectra of CD_3CH_2OH calculated with GVPT2 method at different levels of electronic theory; (a) B3LYP-GD3BJ /6-31G(d,p); (b) B3LYP-GD3BJ /6-31G(d,p)//CPCM; (c) B2PLYP-GD3BJ/6-31G(d,p)//CPCM; (d) MP2/6-31G(d,p)//CPCM; (e) B3LYP-GD3BJ/SNST//CPCM; (f) B2PLYP-GD3BJ/def2-TZVP//CPCM; (g) MP2/aug-cc-pVTZ//CPCM. Experimental spectrum (exp.) of CD_3CH_2OH in CCl_4 (0.1 M).**

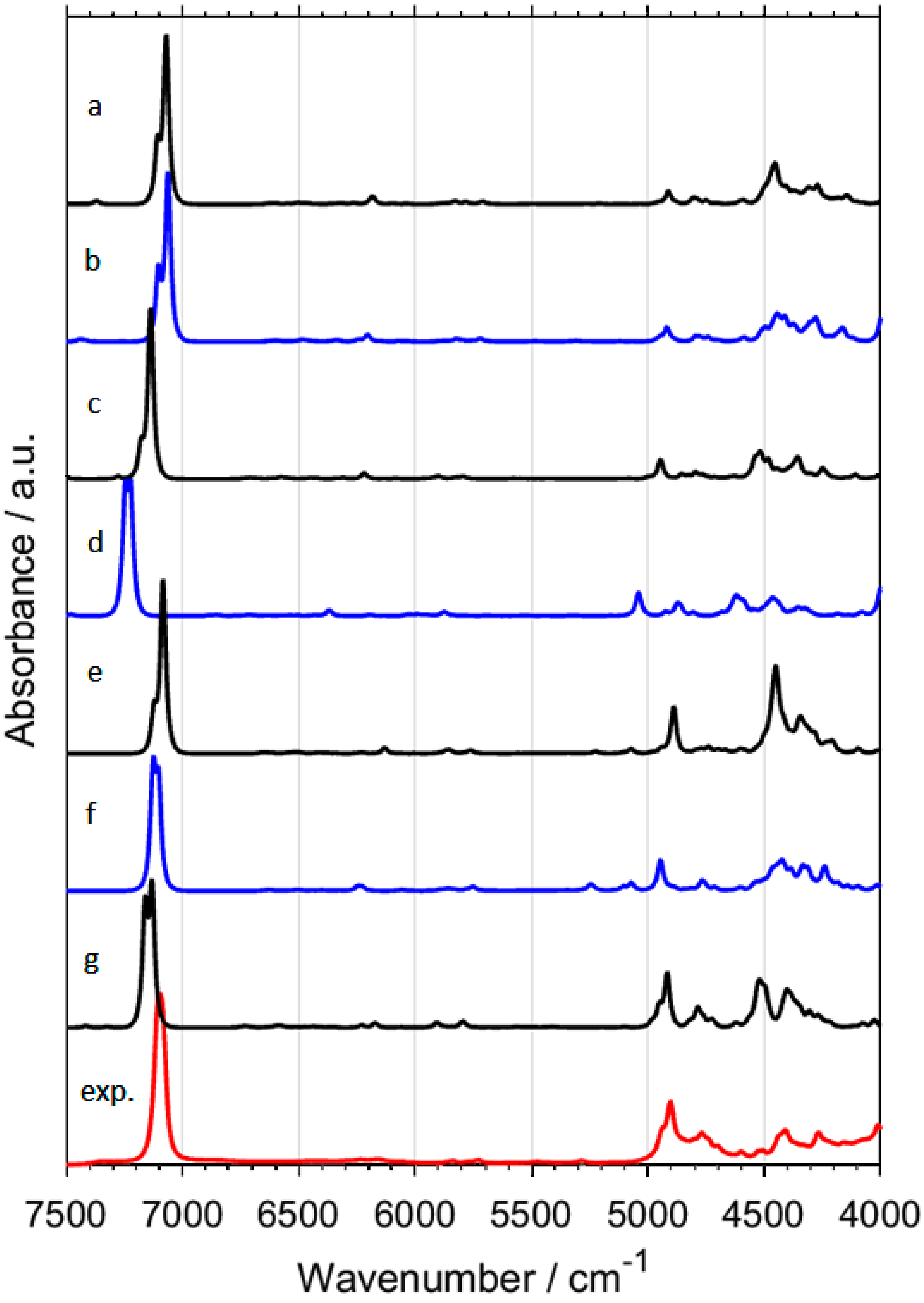

Figure S3. NIR spectra of CD_3CD_2OH calculated with GVPT2 method at different levels of electronic theory; (a) B3LYP-GD3BJ /6-31G(d,p); (b) B3LYP-GD3BJ /6-31G(d,p)//CPCM; (c) B2PLYP-GD3BJ/6-31G(d,p)//CPCM; (d) MP2/6-31G(d,p)//CPCM; (e) B3LYP-GD3BJ/SNST//CPCM; (f) B2PLYP-GD3BJ/def2-TZVP//CPCM; (g) MP2/aug-cc-pVTZ//CPCM. Experimental spectrum (exp.) of CD_3CD_2OH in CCl_4 (0.1 M).

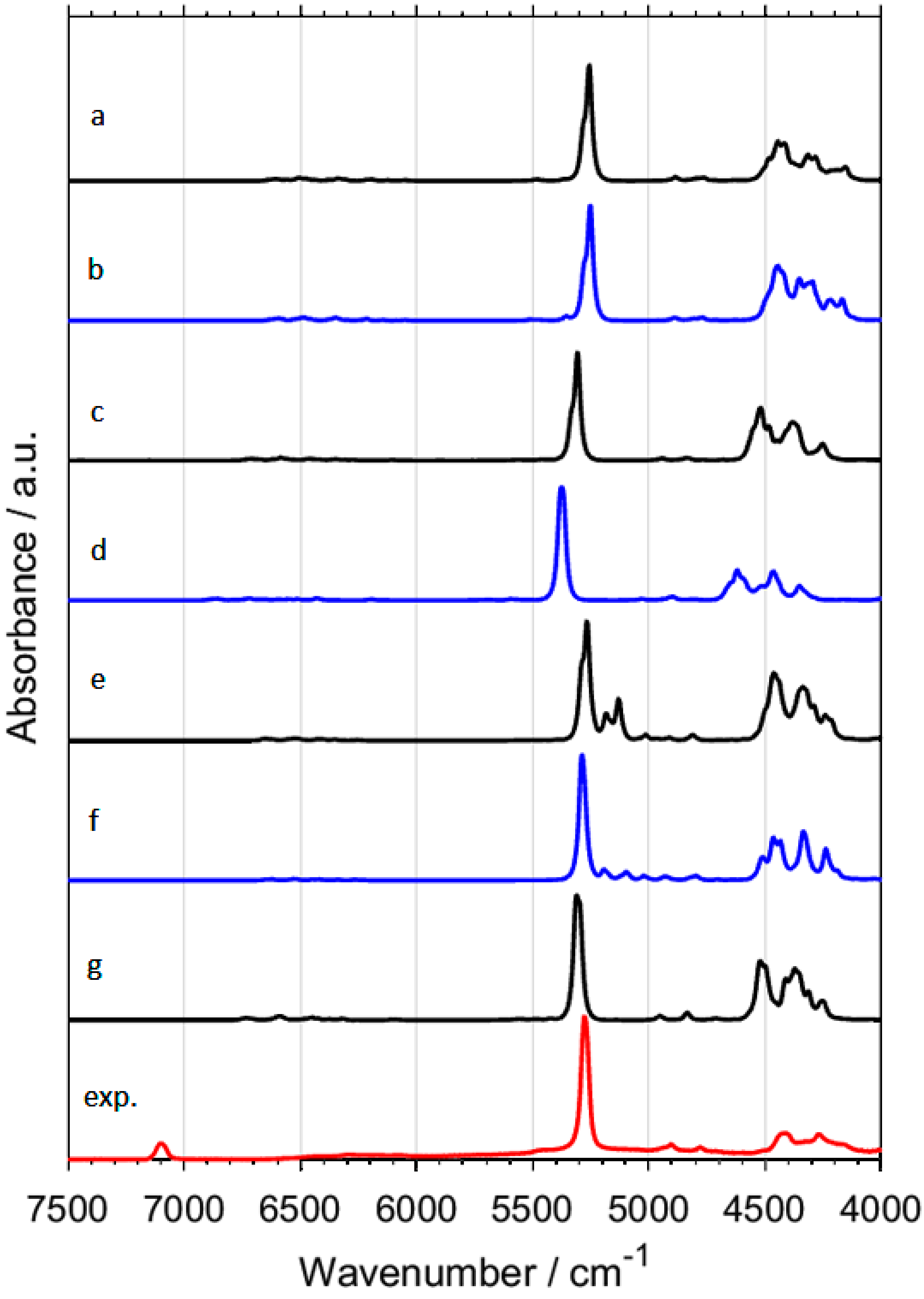

Figure S4. NIR spectra of CD_3CD_2OD calculated with GVPT2 method at different levels of electronic theory; (a) B3LYP-GD3BJ /6-31G(d,p); (b) B3LYP-GD3BJ /6-31G(d,p)//CPCM; (c) B2PLYP-GD3BJ/6-31G(d,p)//CPCM; (d) MP2/6-31G(d,p)//CPCM; (e) B3LYP-GD3BJ/SNST//CPCM; (f) B2PLYP-GD3BJ/def2-TZVP//CPCM; (g) MP2/aug-cc-pVTZ//CPCM. Experimental spectrum (exp.) of CD_3CD_2OD in CCl_4 (0.1 M).

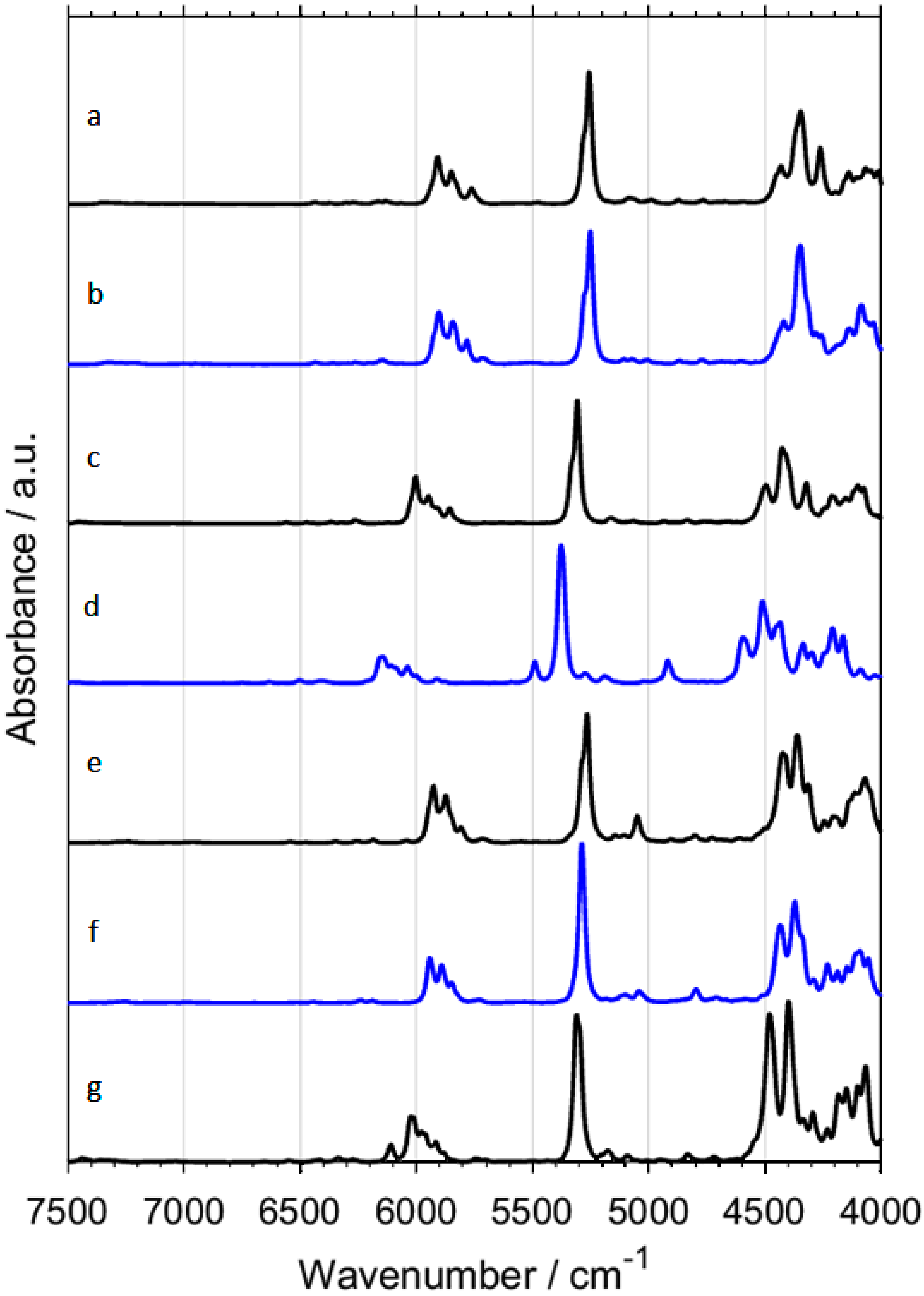

Figure S5. **NIR spectra of CH_3CD_2OD calculated with GVPT2 method at different levels of electronic theory; (a) B3LYP-GD3BJ /6-31G(d,p); (b) B3LYP-GD3BJ /6-31G(d,p)//CPCM; (c) B2PLYP-GD3BJ/6-31G(d,p)//CPCM; (d) MP2/6-31G(d,p)//CPCM; (e) B3LYP-GD3BJ/SNST//CPCM; (f) B2PLYP-GD3BJ/def2-TZVP//CPCM; (g) MP2/aug-cc-pVTZ//CPCM.**

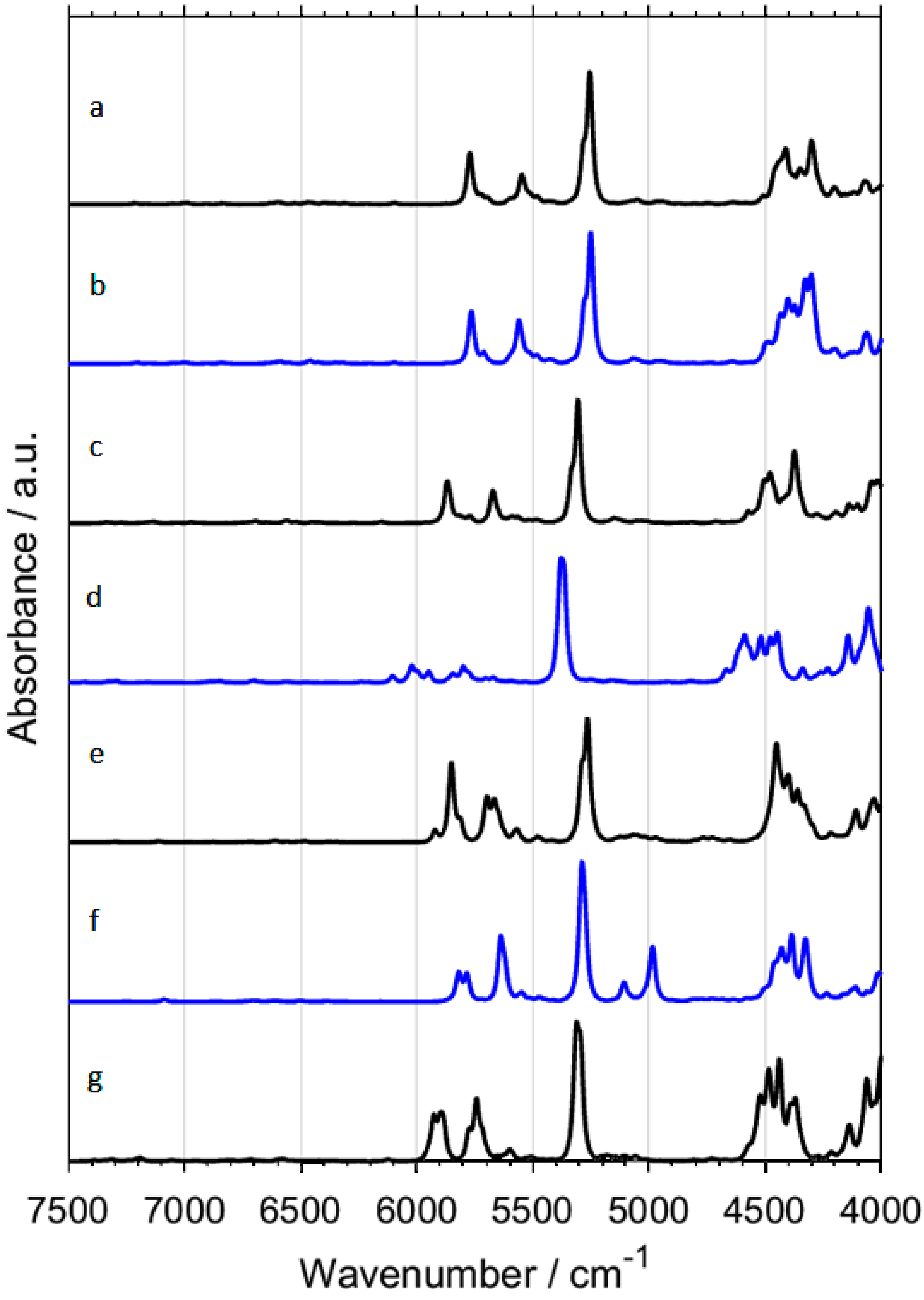

Figure S6. NIR spectra of CD_3CH_2OD calculated with GVPT2 method at different levels of electronic theory; (a) B3LYP-GD3BJ /6-31G(d,p); (b) B3LYP-GD3BJ /6-31G(d,p)//CPCM; (c) B2PLYP-GD3BJ/6-31G(d,p)//CPCM; (d) MP2/6-31G(d,p)//CPCM; (e) B3LYP-GD3BJ/SNST//CPCM; (f) B2PLYP-GD3BJ/def2-TZVP//CPCM; (g) MP2/aug-cc-pVTZ//CPCM.

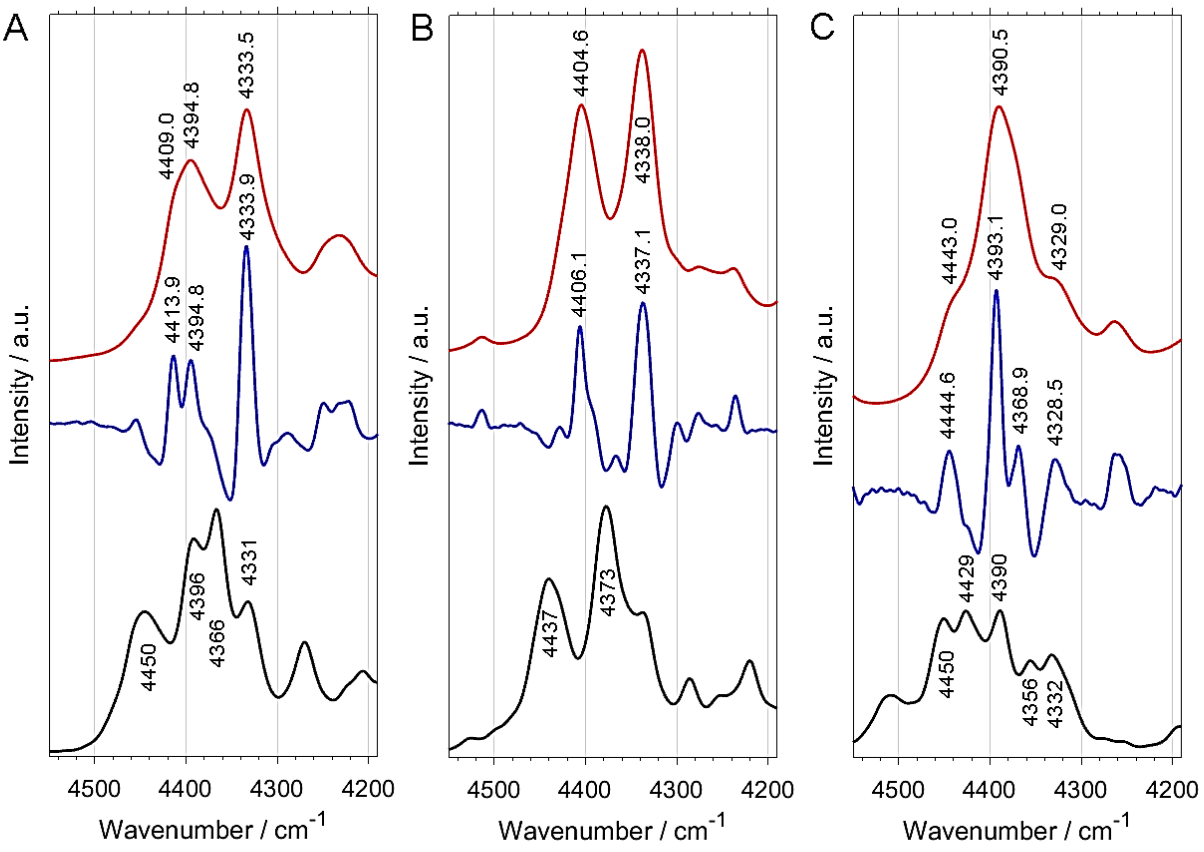

Figure S7. The details of NIR spectra of (A) CH_3CH_2OH; (B) CH_3CD_2OH; (C) CD_3CH_2OH in the 4550-4200 cm^{-1}. From up to bottom: experimental NIR spectrum (red line); second derivative spectrum, multiplied by -1 (blue line); theoretical (B2PLYP-GD3BJ/def2-TZVP//CPCM) spectrum (black line).

molecules

MDPI

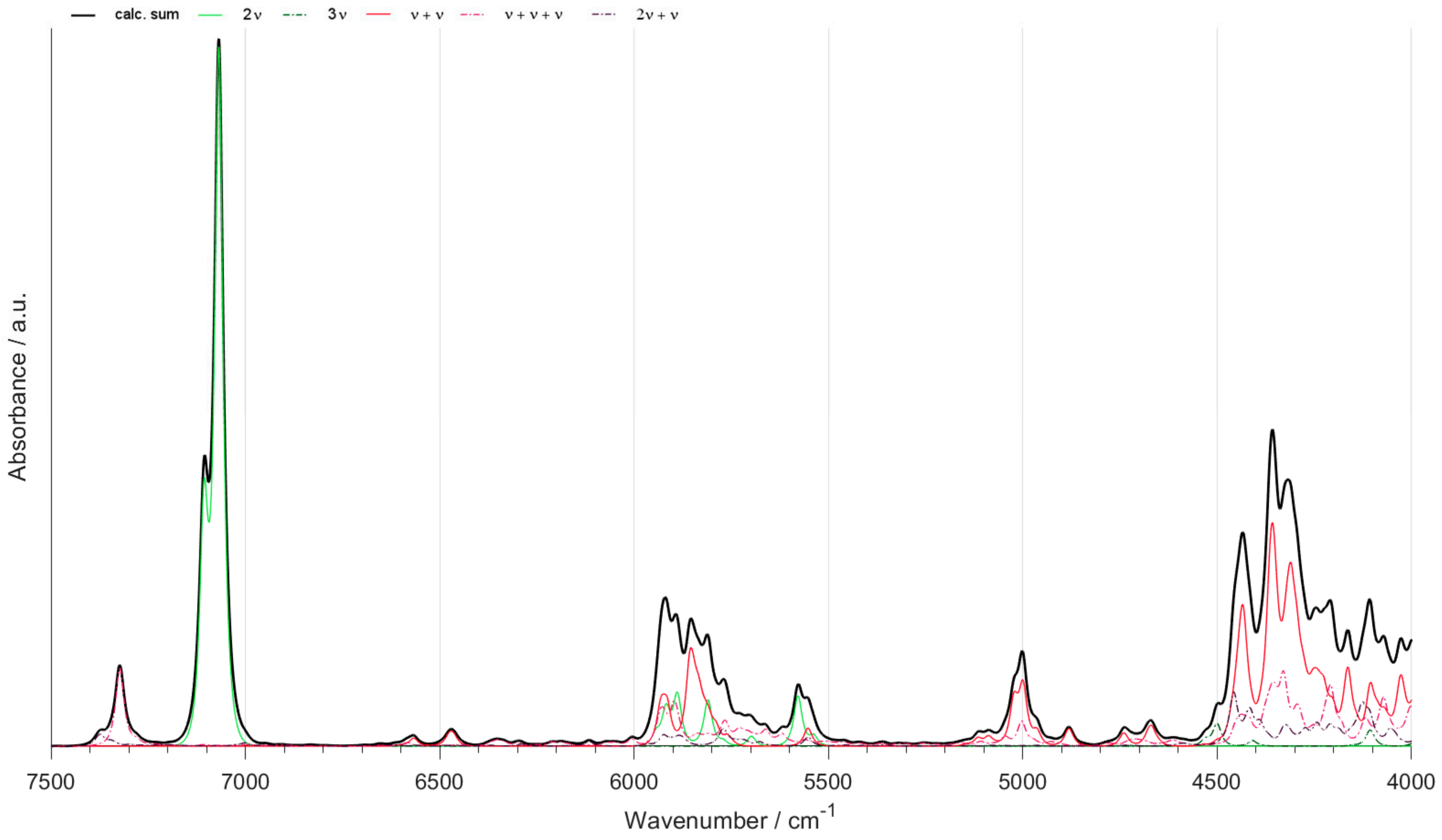

Figure S8. (Enlarged) NIR spectra of CH_3CH_2OH calculated with GVPT2 method at B3LYP-GD3BJ/6-31G(d,p) level of electronic theory

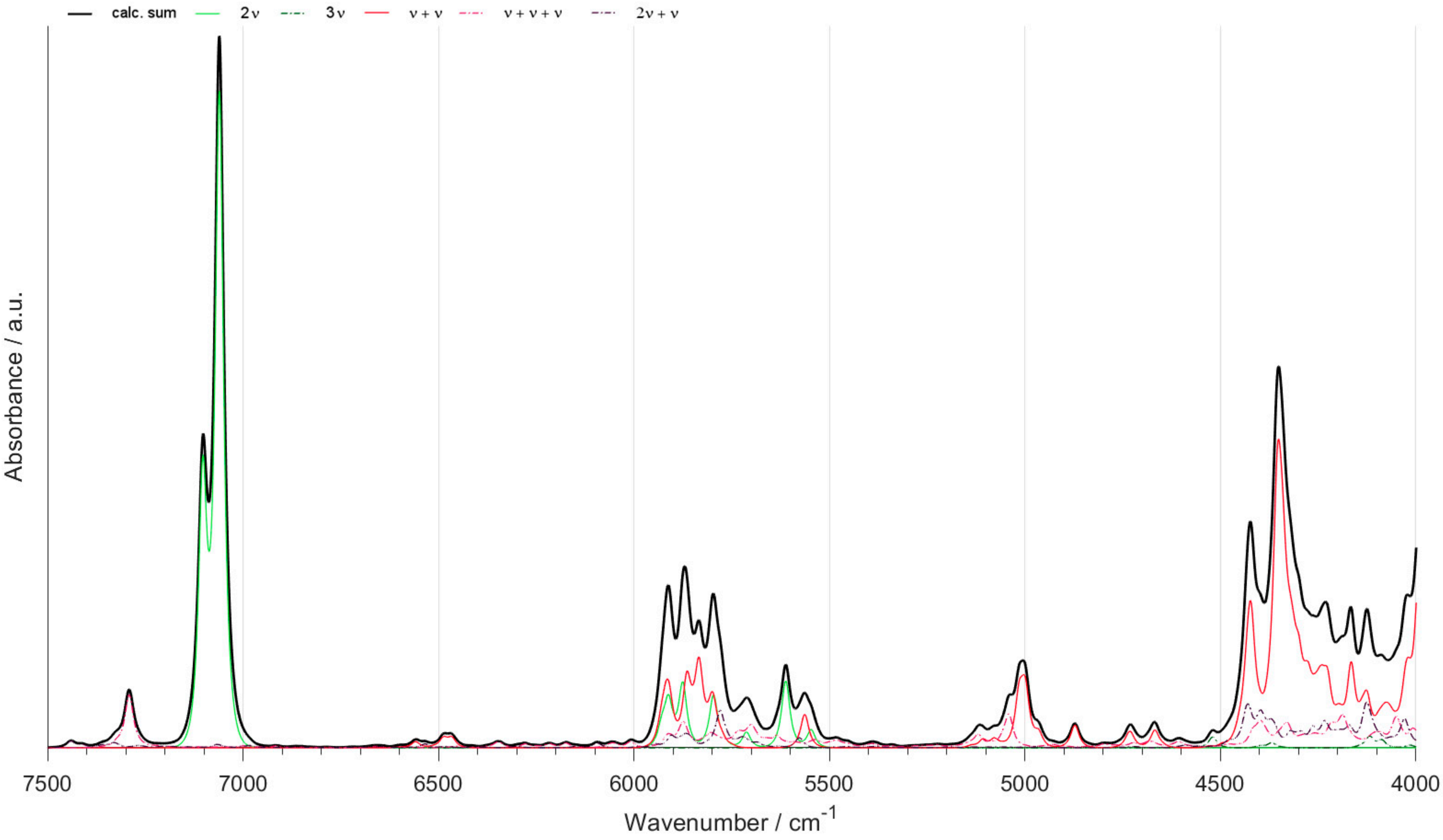

Figure S9. (High-resolution copy of Figure 1) NIR spectra of CH_3CH_2OH calculated with GVPT2 method at B3LYP-GD3BJ/6-31G(d,p)//CPCM level of electronic theory.

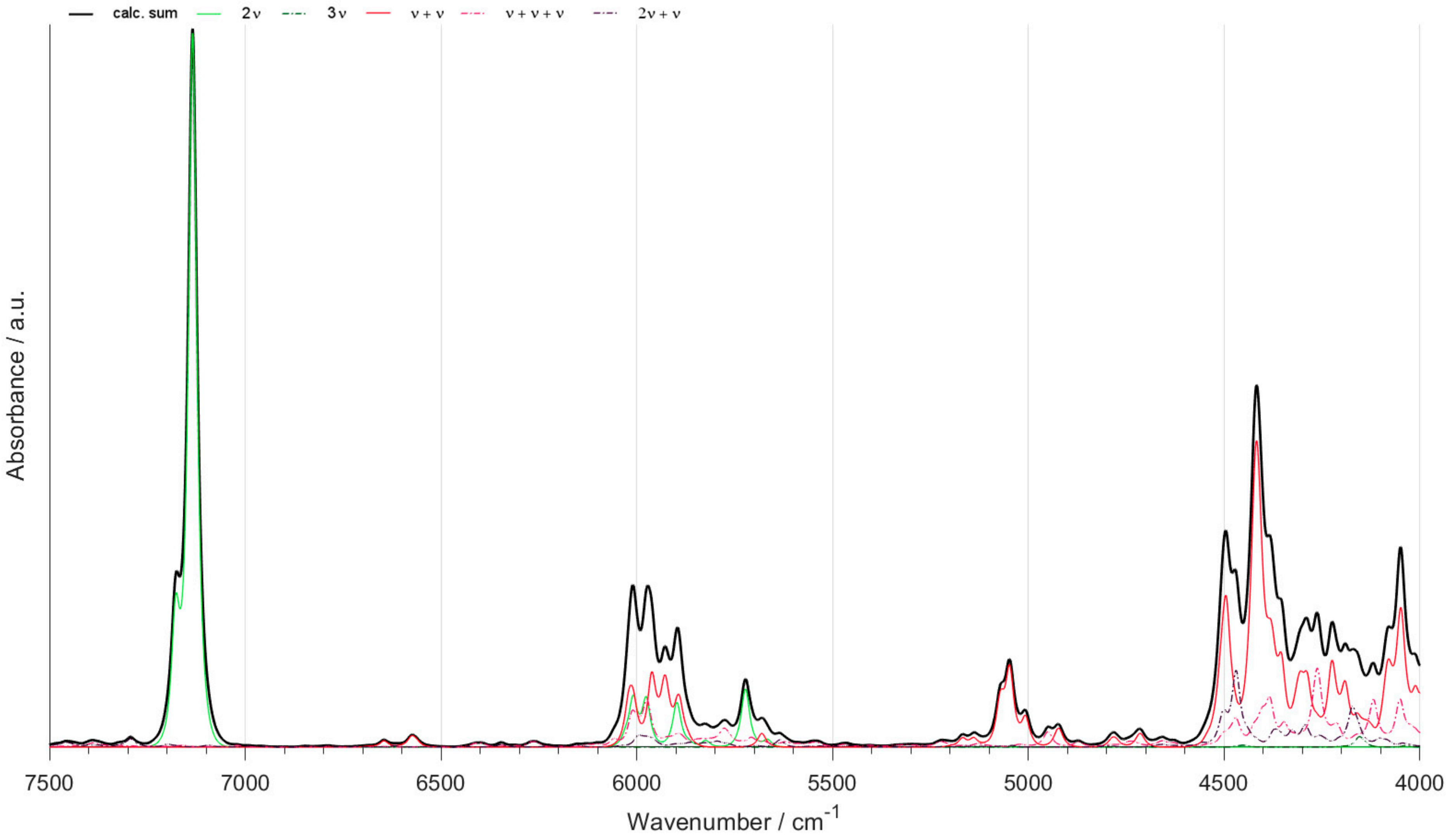

Figure S10. (High-resolution copy of Figure 1) NIR spectra of CH_3CH_2OH calculated with GVPT2 method at B2PLYP-GD3BJ/6-31G(d,p)//CPCM level of electronic theory.

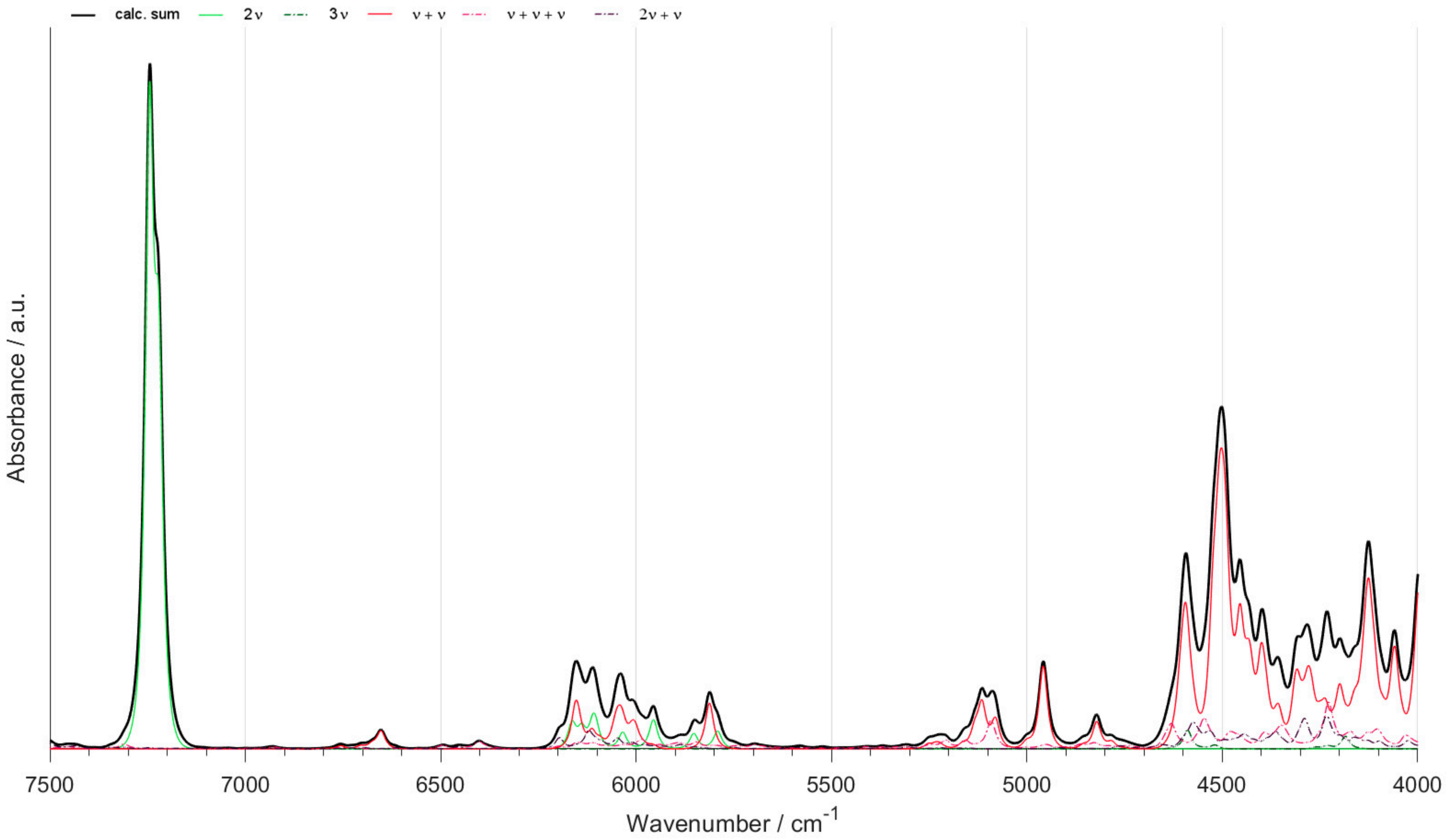

#

Figure S11. (High-resolution copy of Figure 1) NIR spectra of CH_3CH_2OH calculated with GVPT2 method at MP2/6-31G(d,p)//CPCM level of electronic theory.

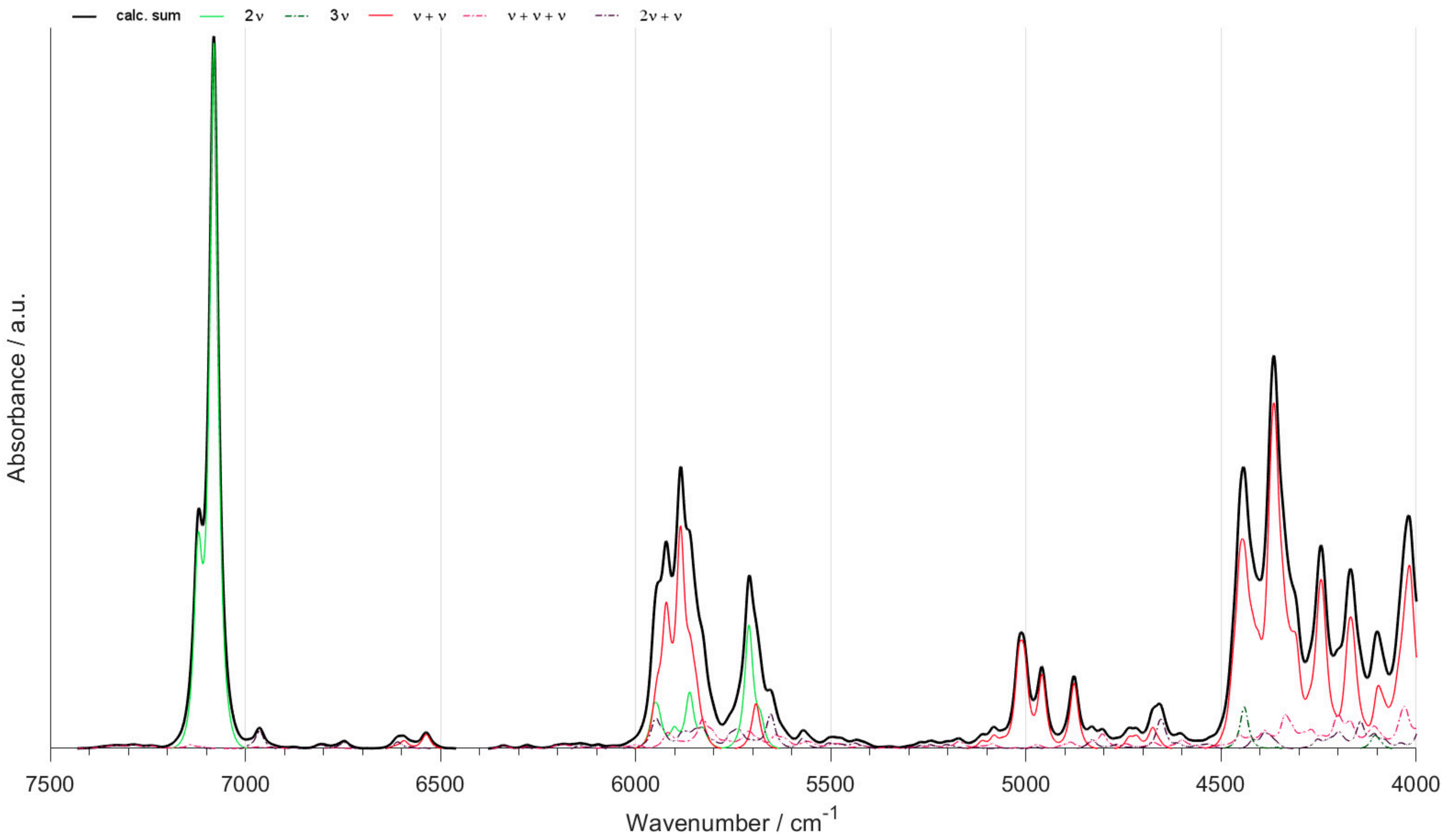

Figure S12. (High-resolution copy of Figure 1) NIR spectra of CH_3CH_2OH calculated with GVPT2 method at B3LYP-GD3BJ/SNST//CPCM level of electronic theory.

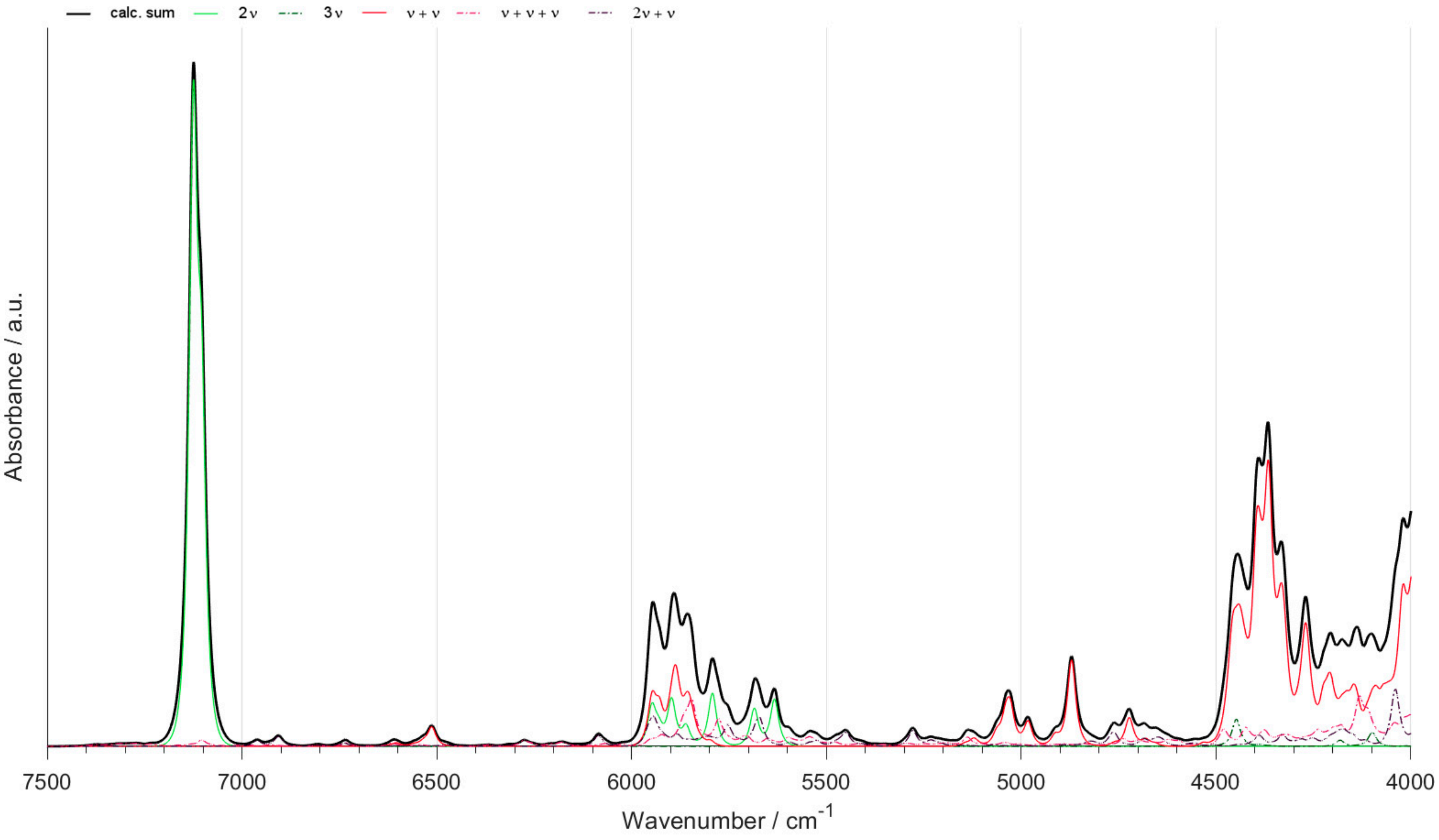

Figure S13. (High-resolution copy of Figure 1) NIR spectra of CH_3CH_2OH calculated with GVPT2 method at B2PLYP-GD3BJ/def2-TZVP//CPCM level of electronic theory.

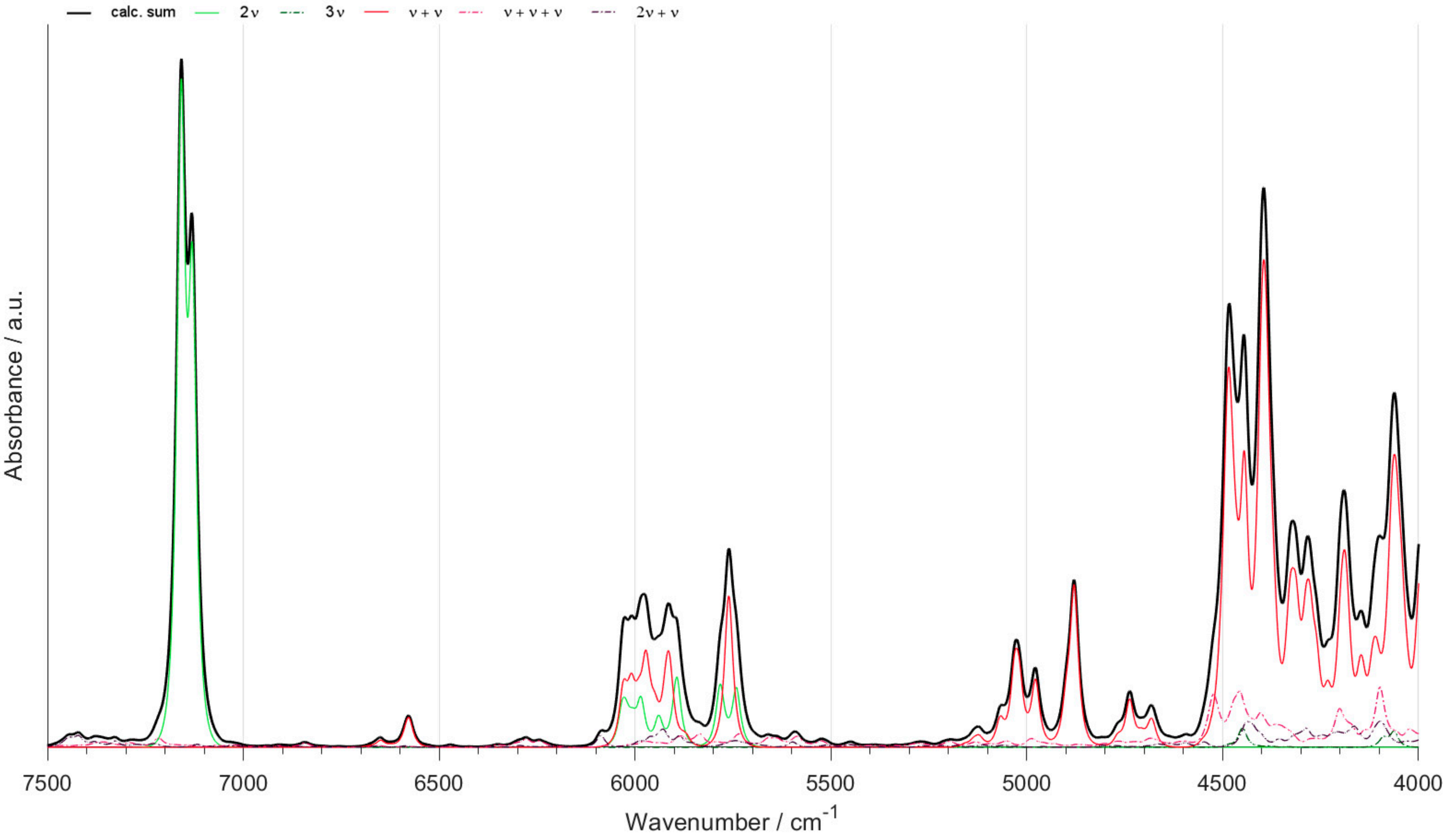

Figure S14. (High-resolution copy of Figure 1) NIR spectra of CH_3CH_2OH calculated with GVPT2 method at MP2/aug-cc-pVTZ//CPCM level of electronic theory.

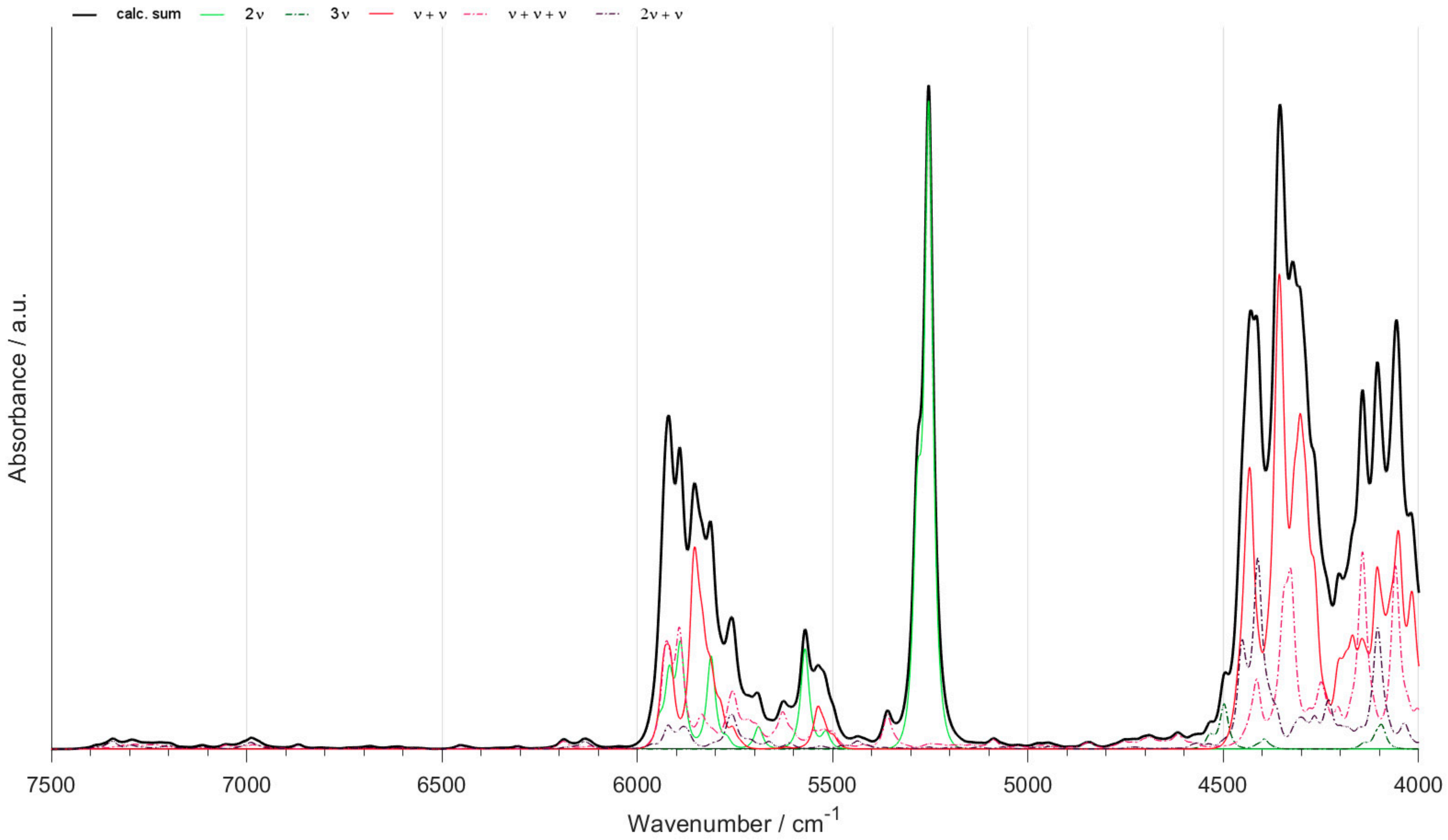

Figure S15. (High-resolution copy of Figure 2) NIR spectra of CH_3CH_2OD calculated with GVPT2 method at B3LYP-GD3BJ/6-31G(d,p) level of electronic theory.

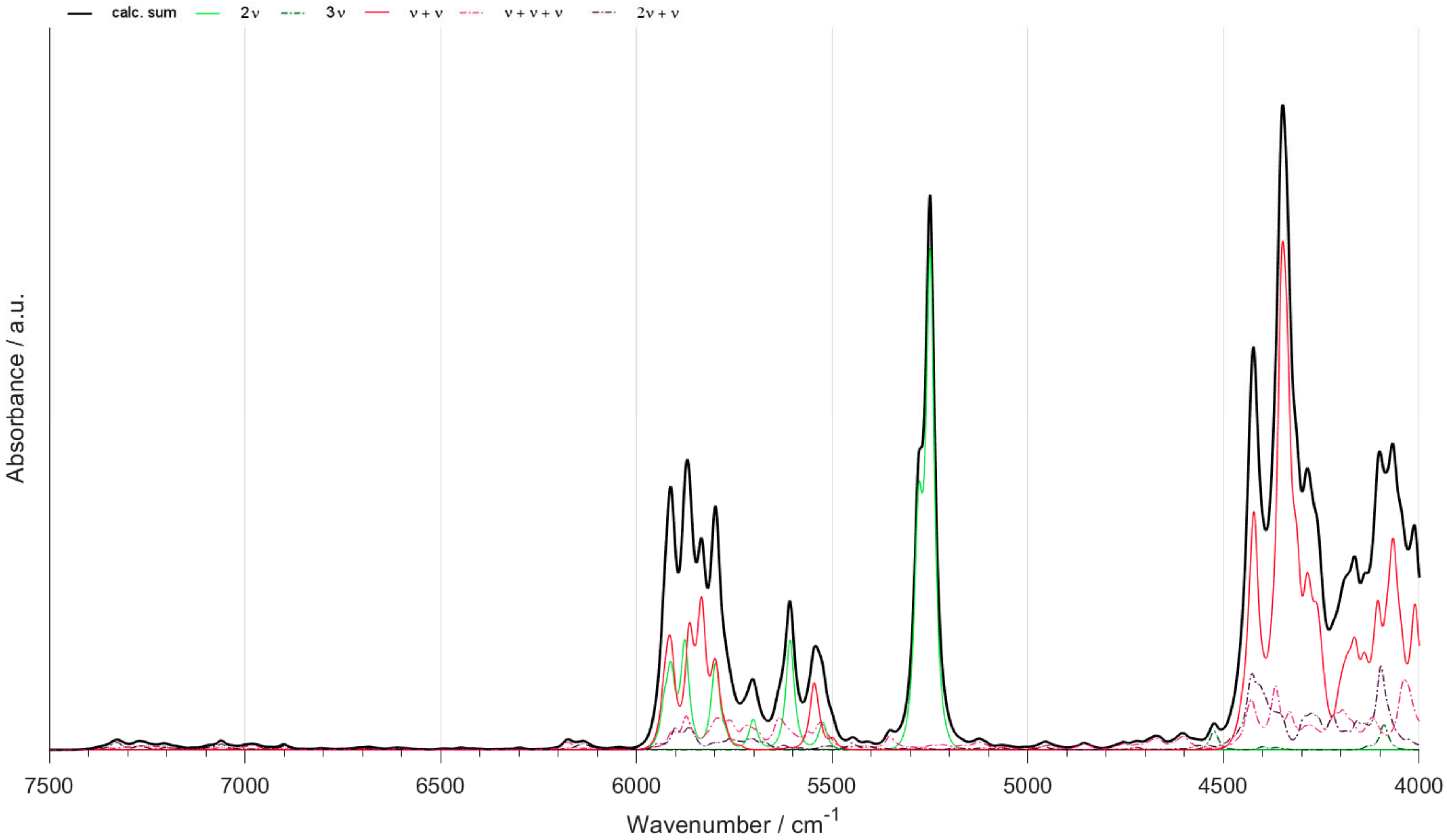

Figure S16. (High-resolution copy of Figure 2) NIR spectra of CH_3CH_2OD calculated with GVPT2 method at B3LYP-GD3BJ/6-31G(d,p)//CPCM level of electronic theory.

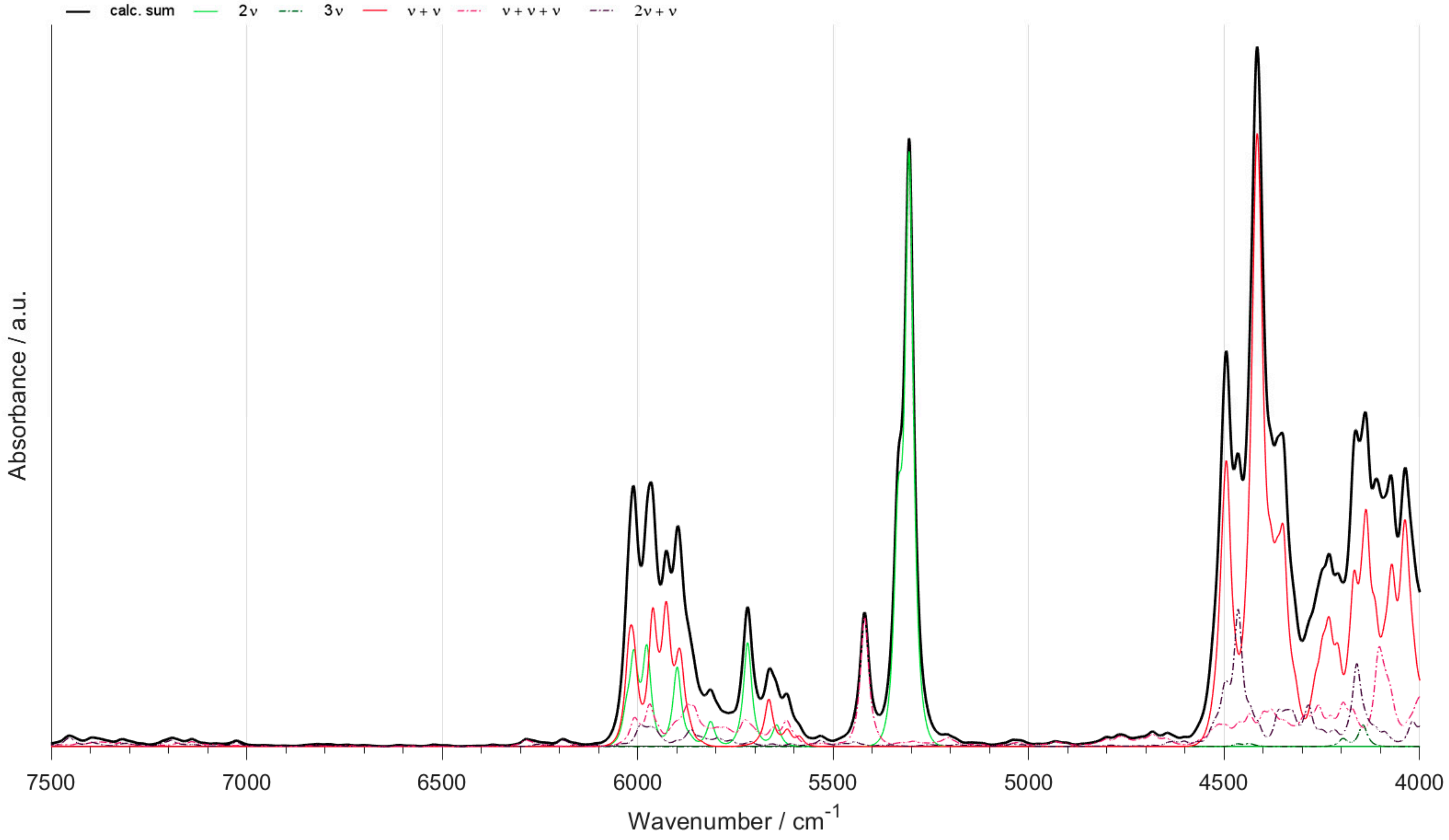

Figure S17. (High-resolution copy of Figure 2) NIR spectra of CH_3CH_2OD calculated with GVPT2 method at B2PLYP-GD3BJ/6-31G(d,p)//CPCM level of electronic theory.

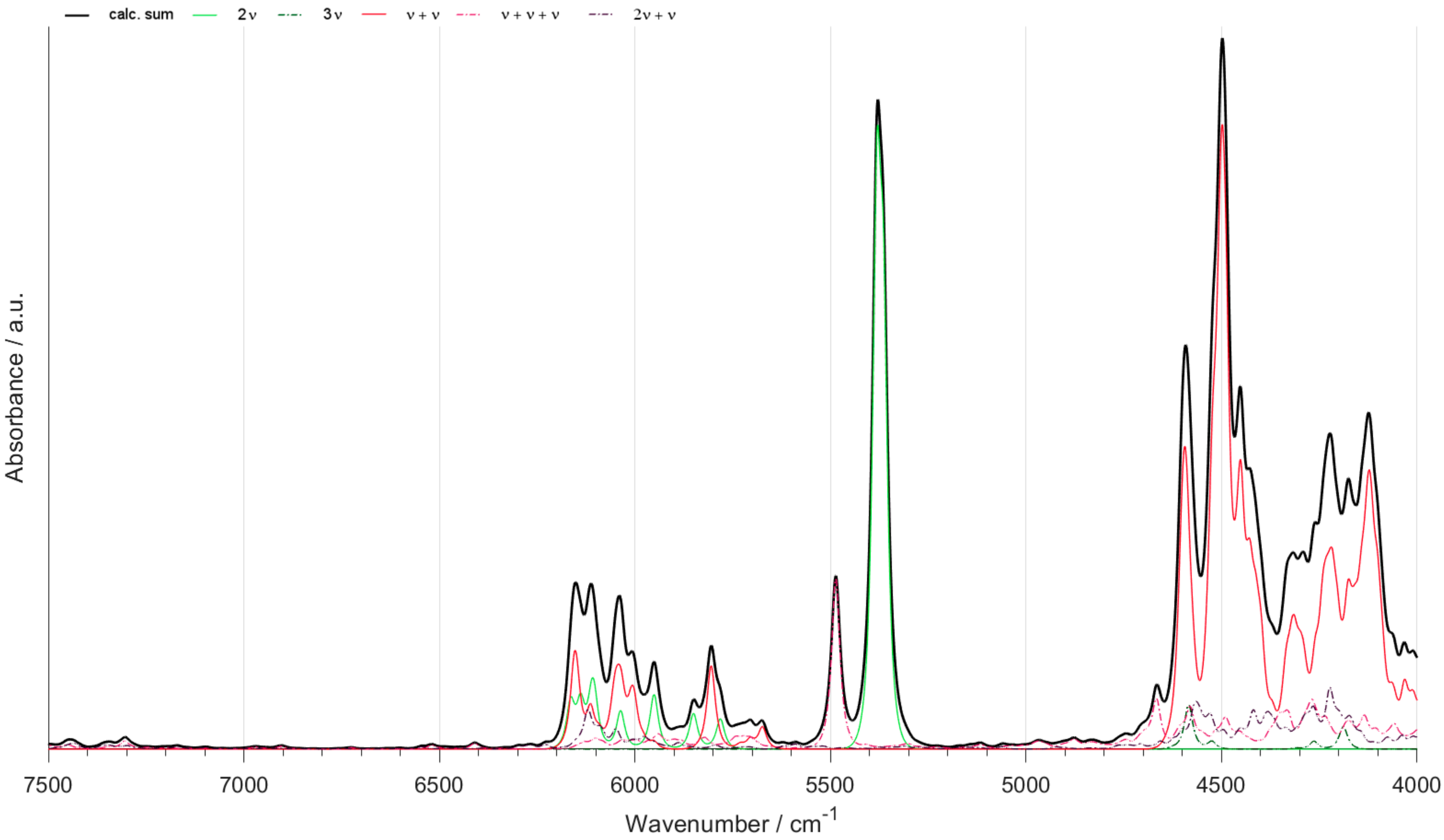

Figure S18. (High-resolution copy of Figure 2) NIR spectra of CH_3CH_2OD calculated with GVPT2 method at MP2/6-31G(d,p)//CPCM t level of electronic theory.

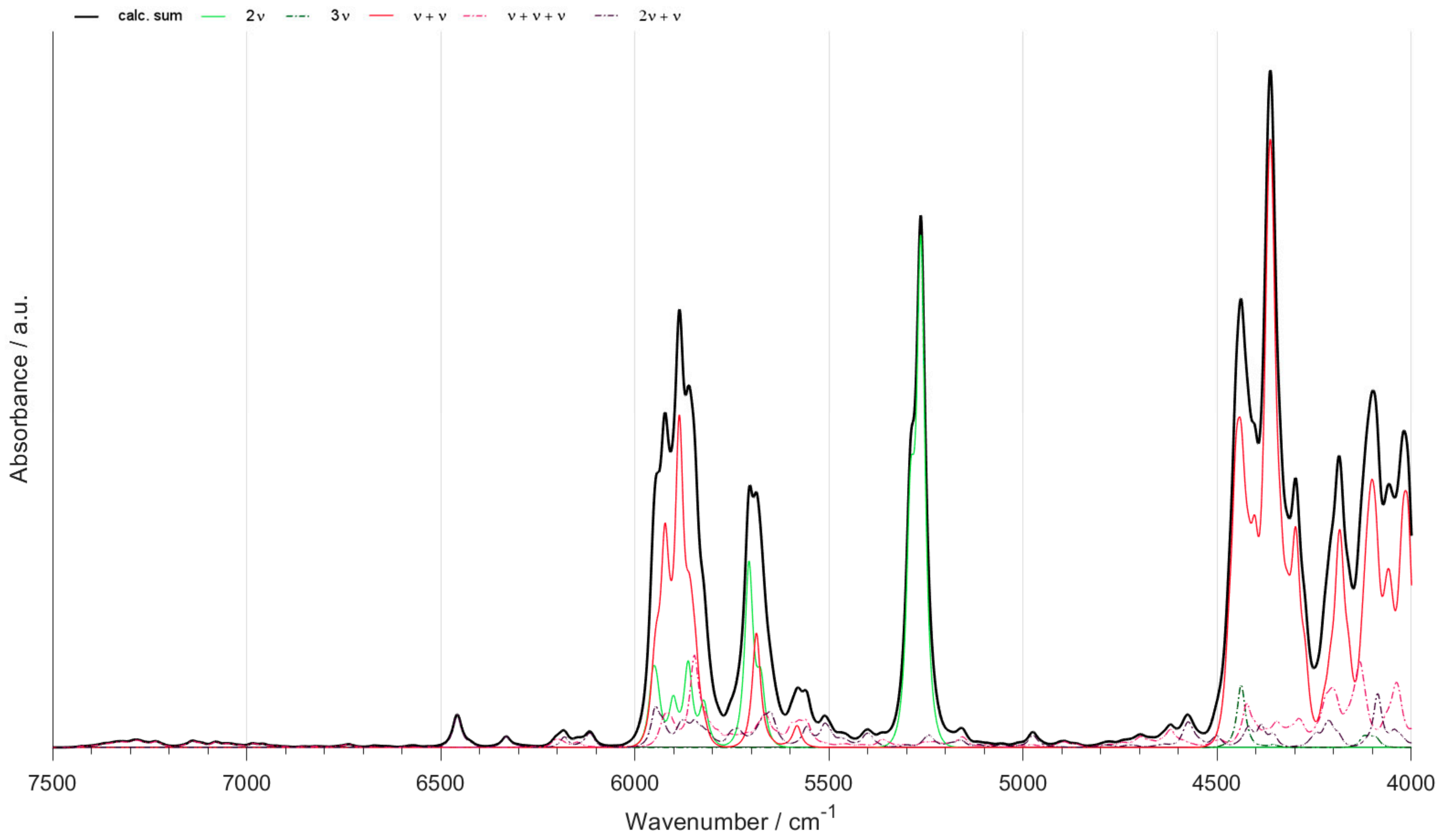

Figure S19. (High-resolution copy of Figure 2) NIR spectra of CH_3CH_2OD calculated with GVPT2 method at B3LYP-GD3BJ/SNST//CPCM level of electronic theory.

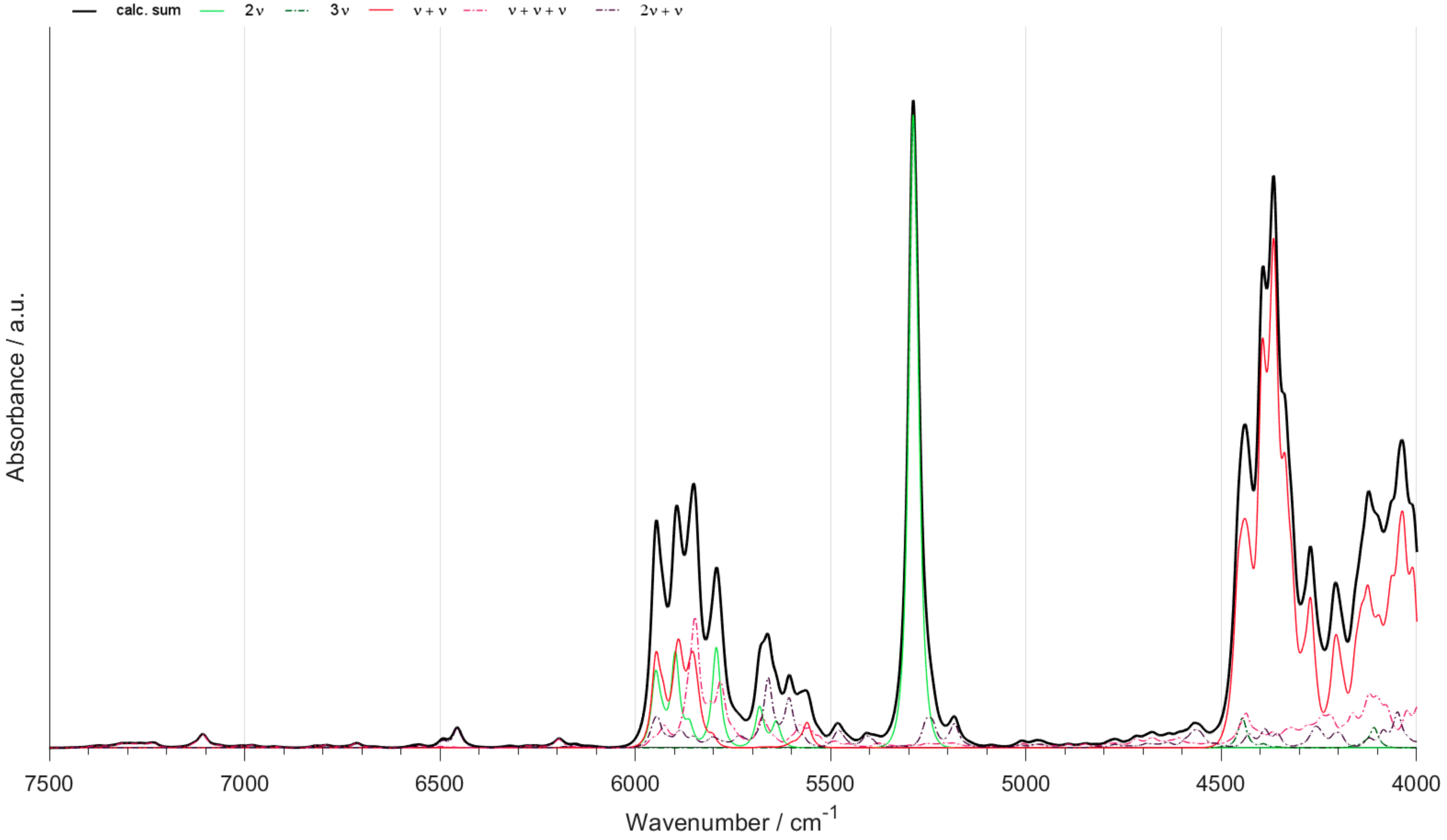

Figure S20. (High-resolution copy of Figure 2) NIR spectra of CH_3CH_2OD calculated with GVPT2 method at B2PLYP-GD3BJ/def2-TZVP//CPCM level of electronic theory.

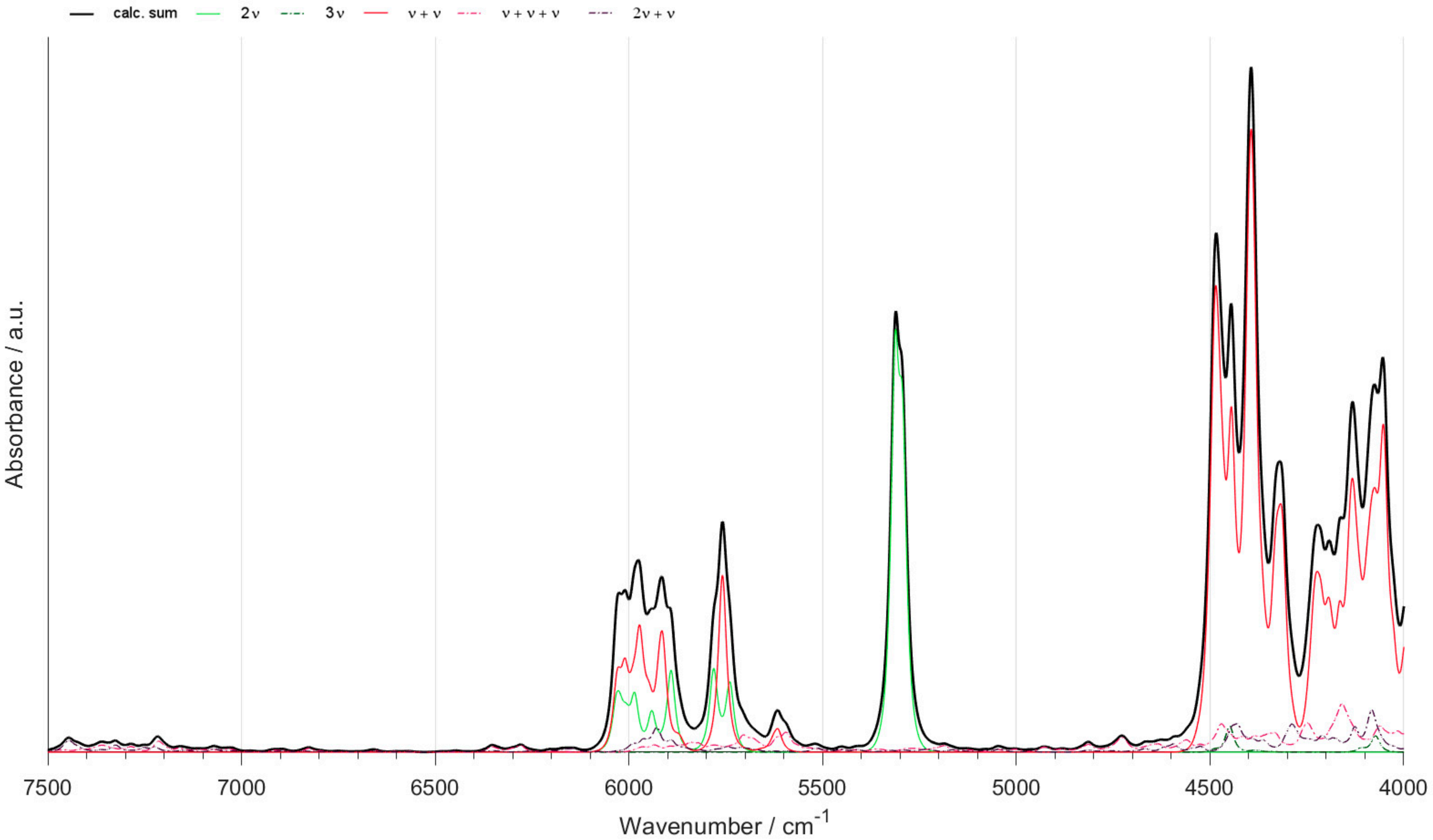

Figure S21. (High-resolution copy of Figure 2) NIR spectra of CH_3CH_2OD calculated with GVPT2 method at MP2/aug-cc-pVTZ//CPCM level of electronic theory.

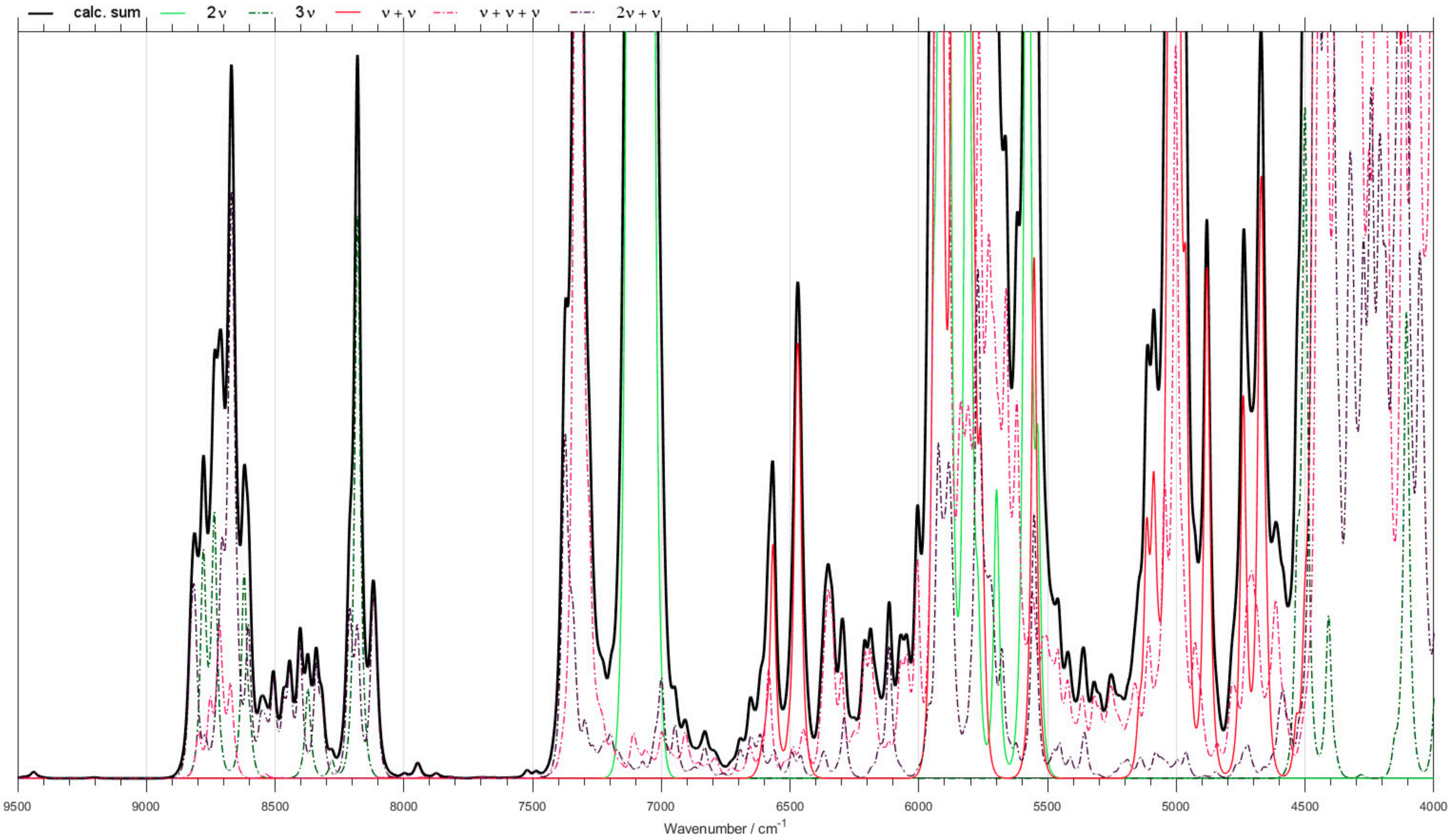

Figure S22. (High-resolution copy of Figure 3) Contributions from minor bands in NIR spectra of CH_3CH_2OH calculated with GVPT2 method at B3LYP-GD3BJ /6-31G(d,p) level of electronic theory.

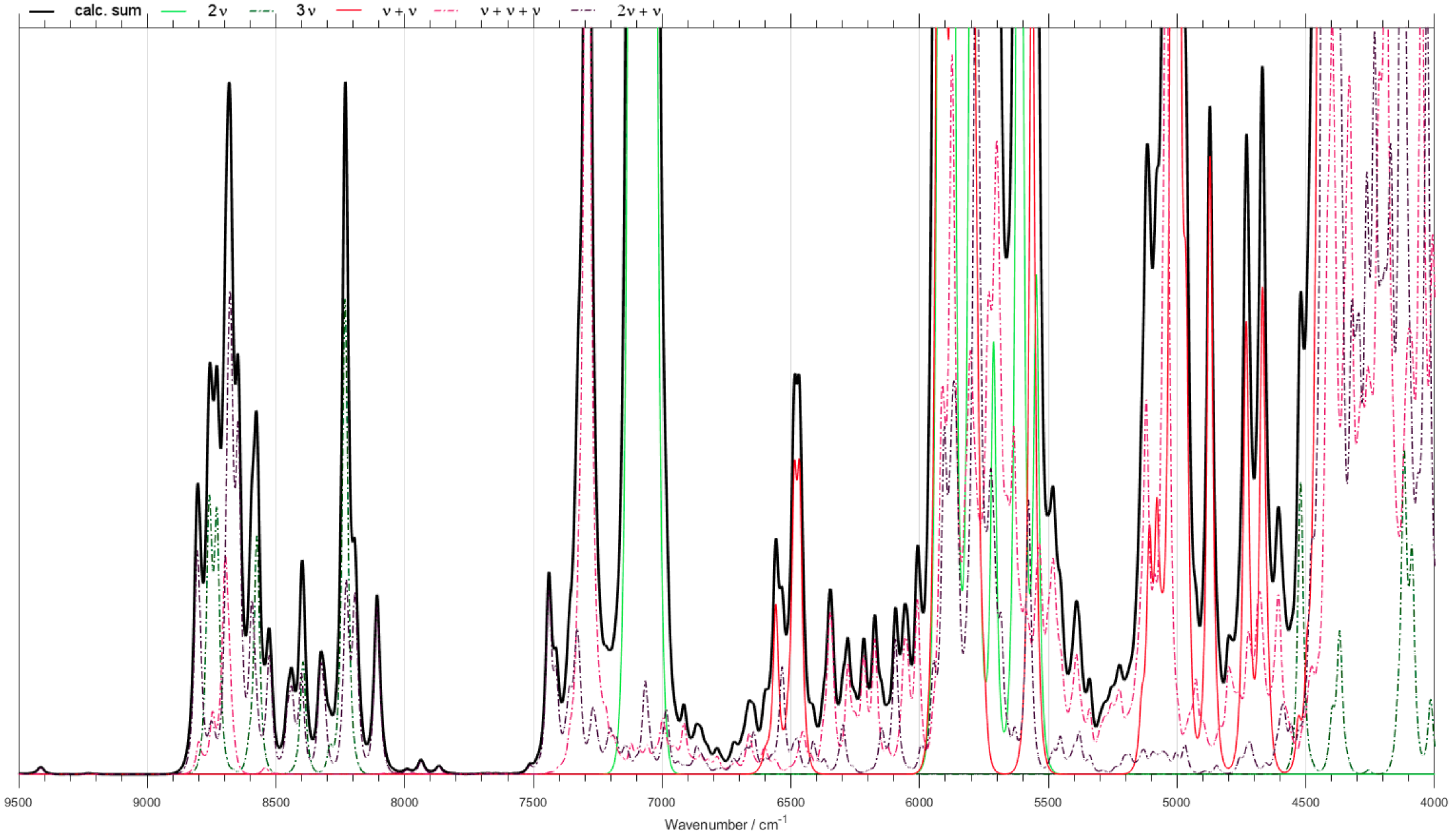

Figure S23. **(High-resolution copy of Figure 3) Contributions from minor bands in NIR spectra of CH_3CH_2OH calculated with GVPT2 method at B3LYP-GD3BJ /6-31G(d,p)//CPCM level of electronic theory.**

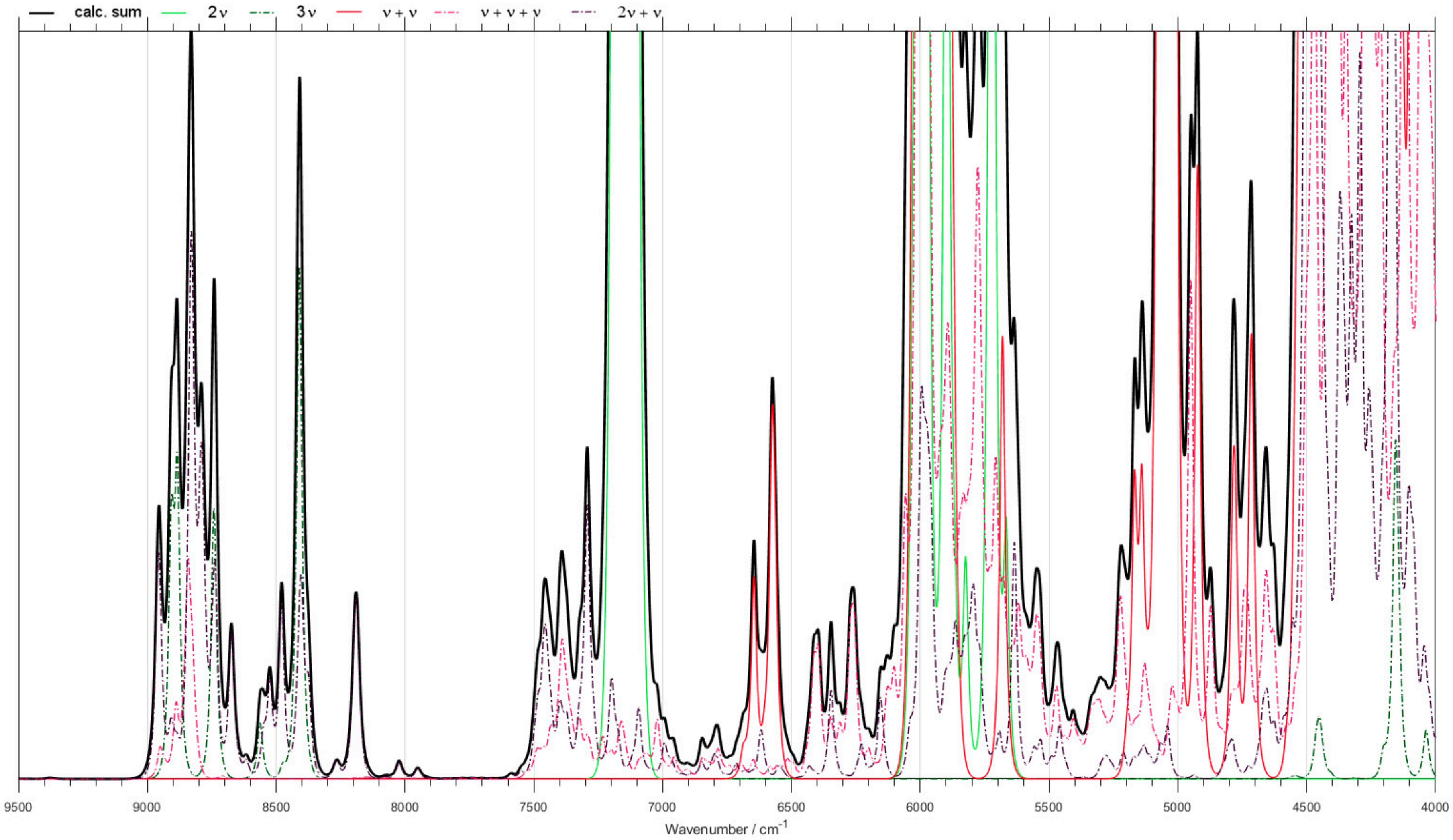

Figure S24. **(High-resolution copy of Figure 3) Contributions from minor bands in NIR spectra of CH_3CH_2OH calculated with GVPT2 method at B2PLYP-GD3BJ/6-31G(d,p)//CPCM level of electronic theory.**

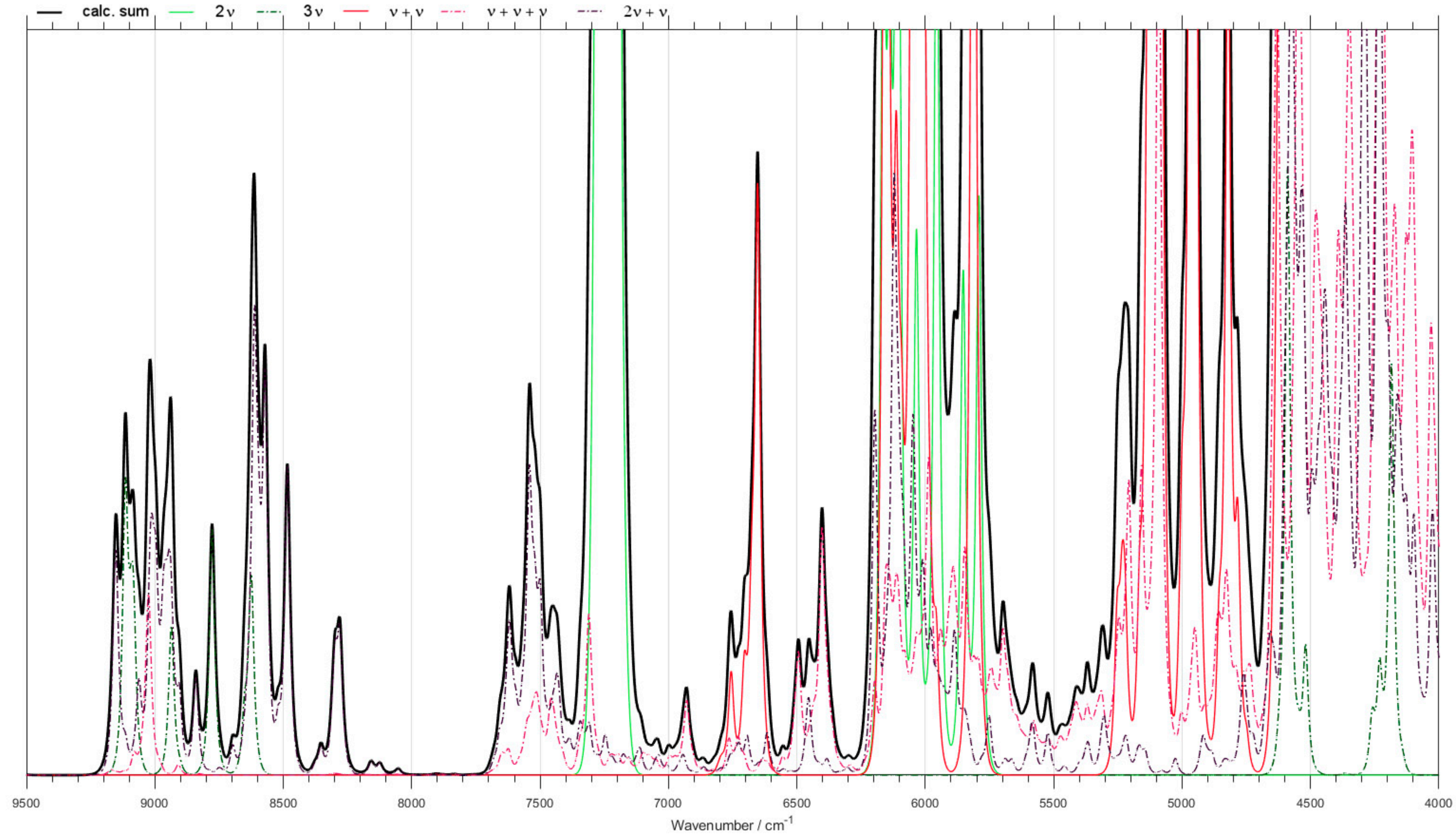

Figure S25. (High-resolution copy of Figure 2) Contributions from minor bands in NIR spectra of CH_3CH_2OH calculated with GVPT2 method at MP2/6-31G(d,p)//CPCM level of electronic theory.

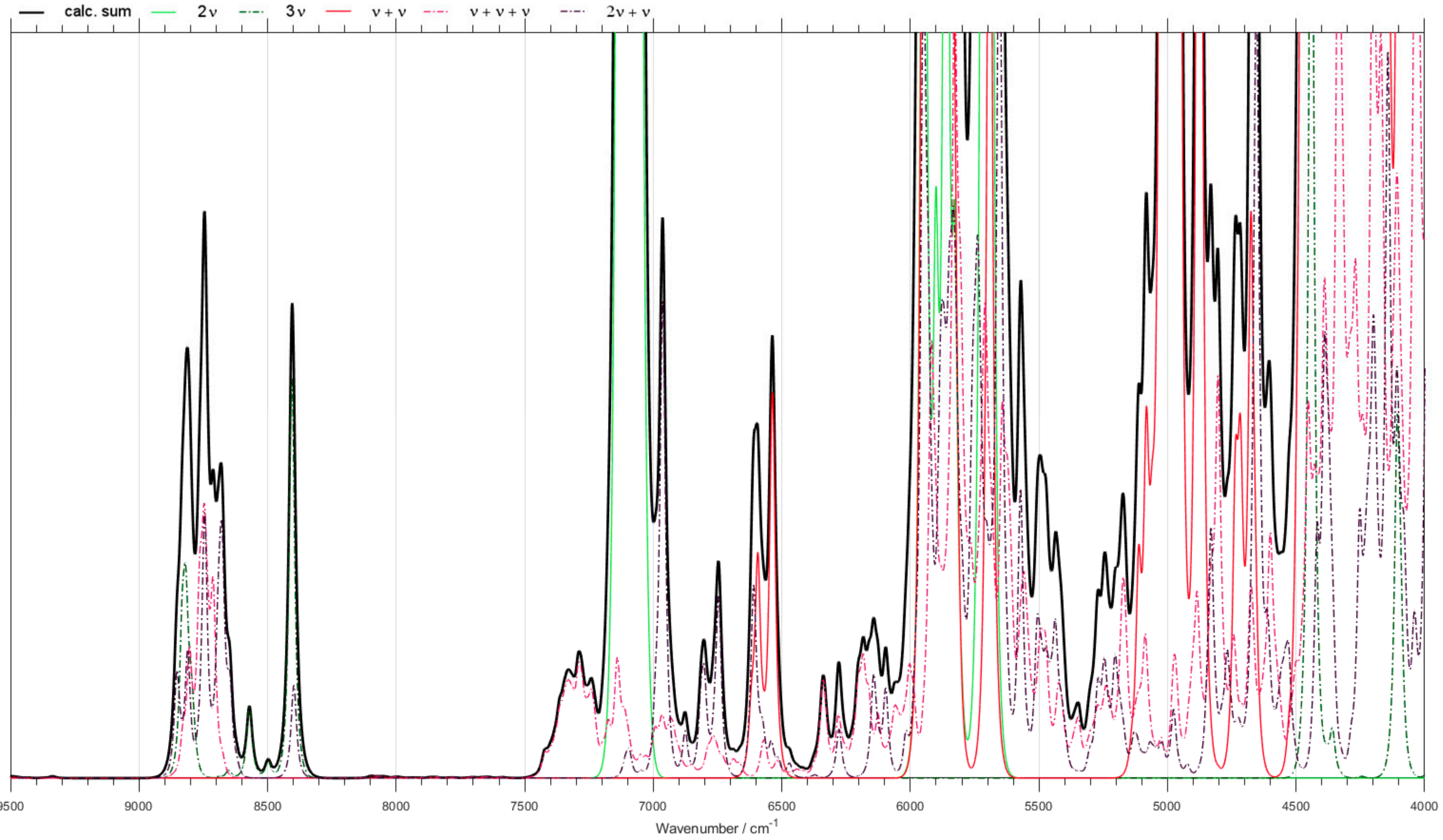

Figure S26. (High-resolution copy of Figure 3) Contributions from minor bands in NIR spectra of CH_3CH_2OH calculated with GVPT2 method at B3LYP-GD3BJ/SNST//CPCM level of electronic theory.

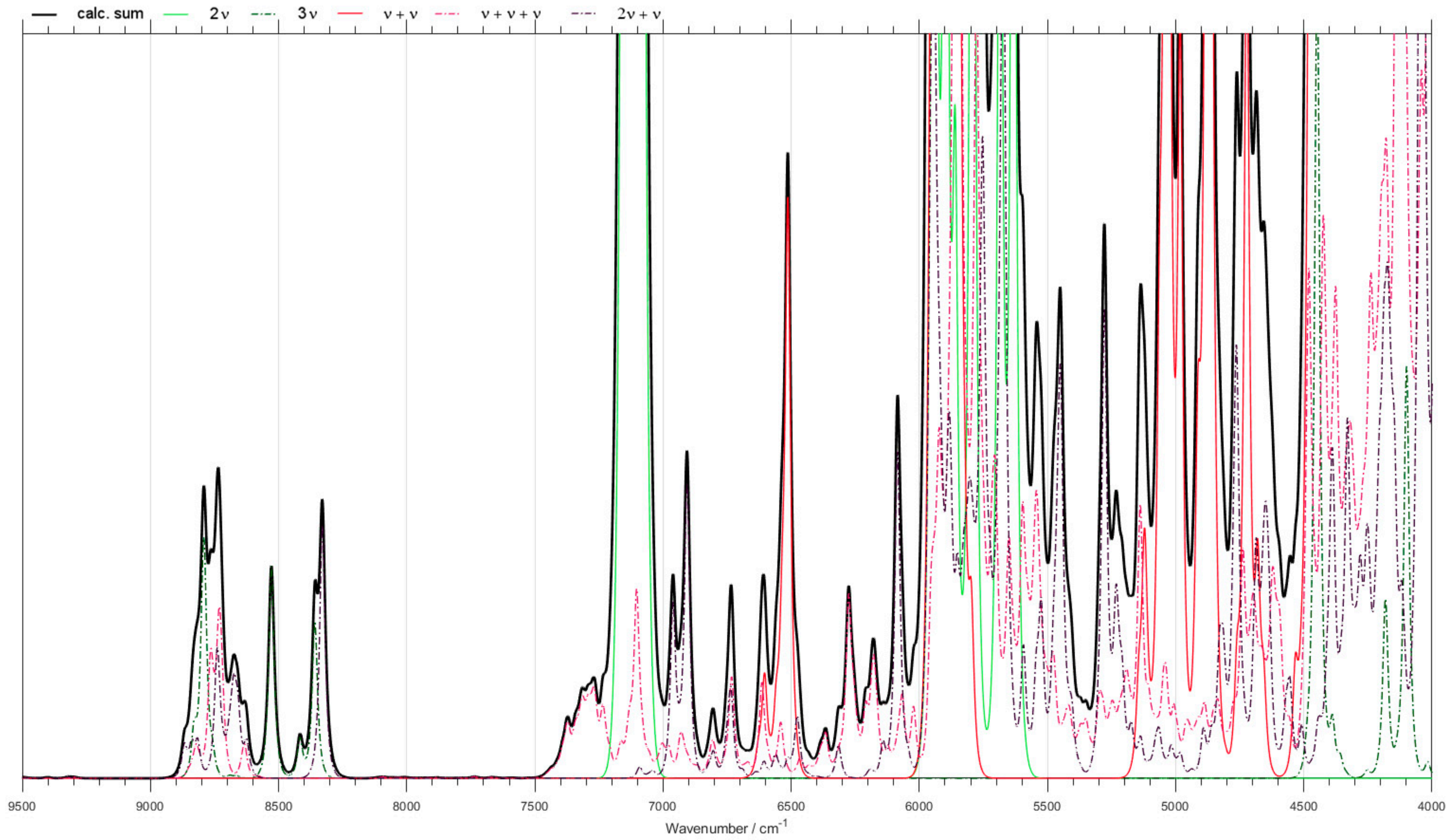

Figure S27. (**High-resolution copy of Figure 3**) **Contributions from minor bands in NIR spectra of CH_3CH_2OH calculated with GVPT2 method at B2PLYP-GD3BJ/def2-TZVP//CPCM level of electronic theory.**

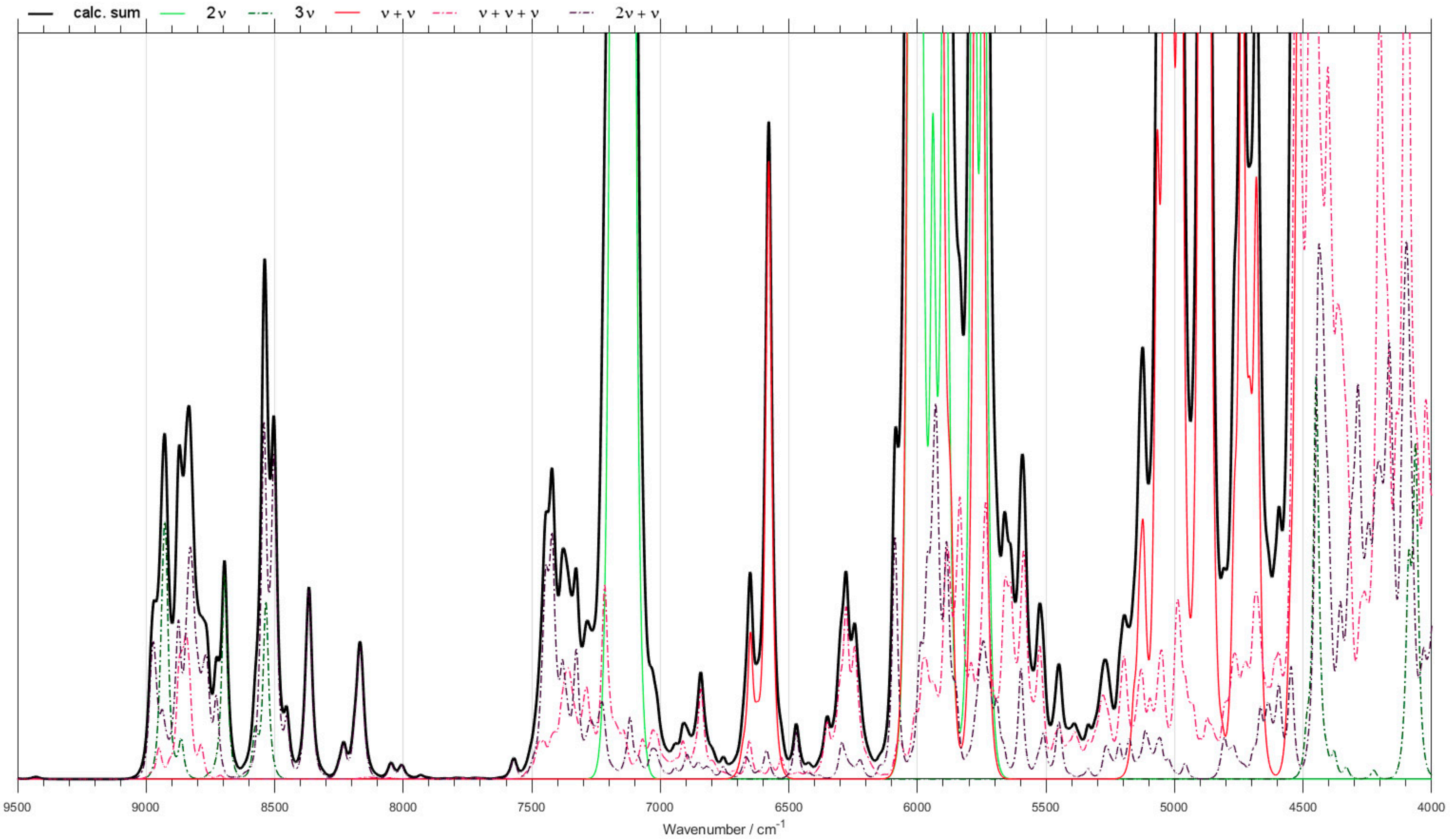

Figure S28. (High-resolution copy of Figure 3) Contributions from minor bands in NIR spectra of CH_3CH_2OH calculated with GVPT2 method at MP2/aug-cc-pVTZ//CPCM level of electronic theory.

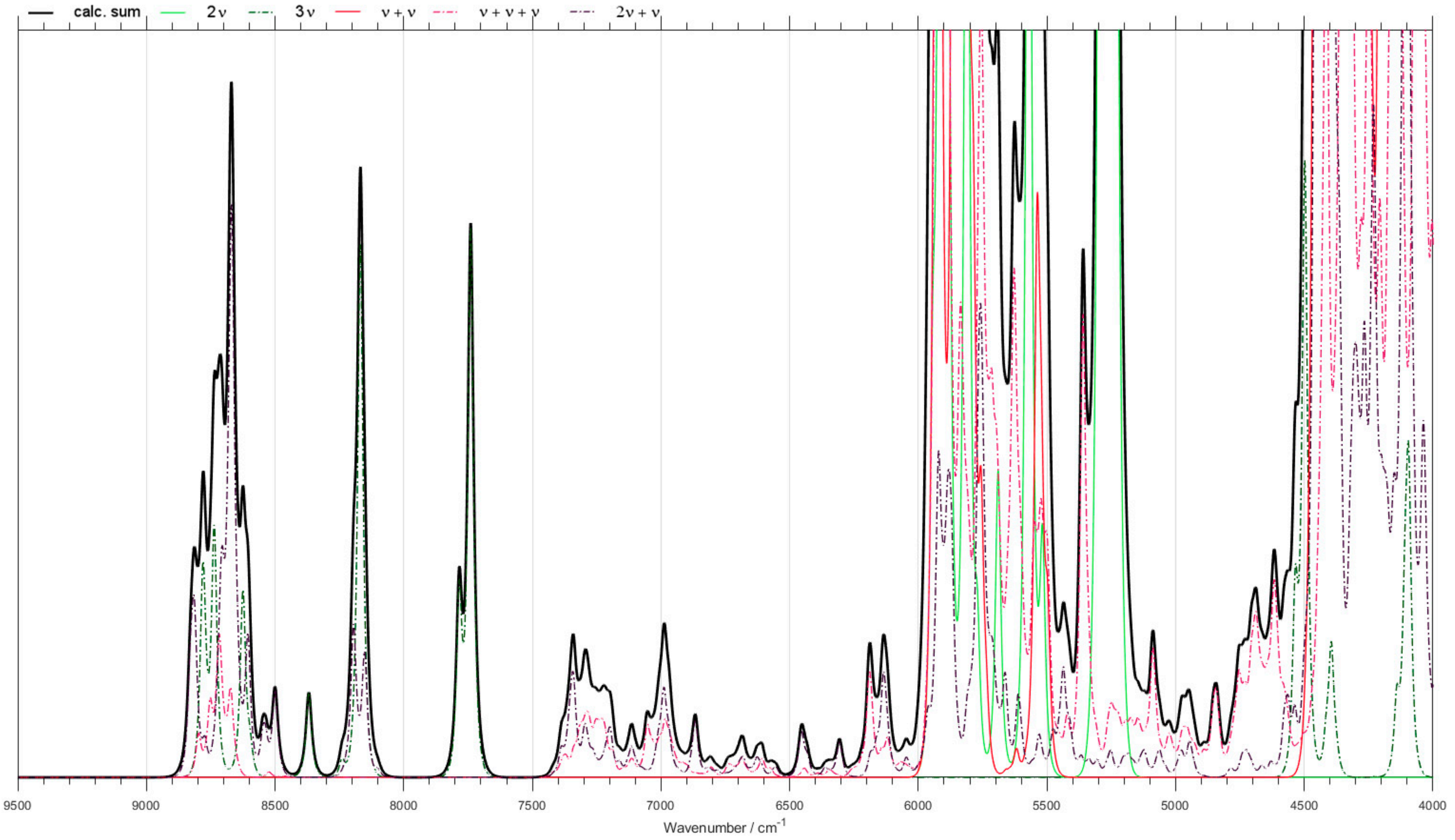

Figure S29. (High-resolution copy of Figure 4) Contributions from minor bands in NIR spectra of CH_3CH_2OD calculated with GVPT2 method at B3LYP-GD3BJ /6-31G(d,p) level of electronic theory.

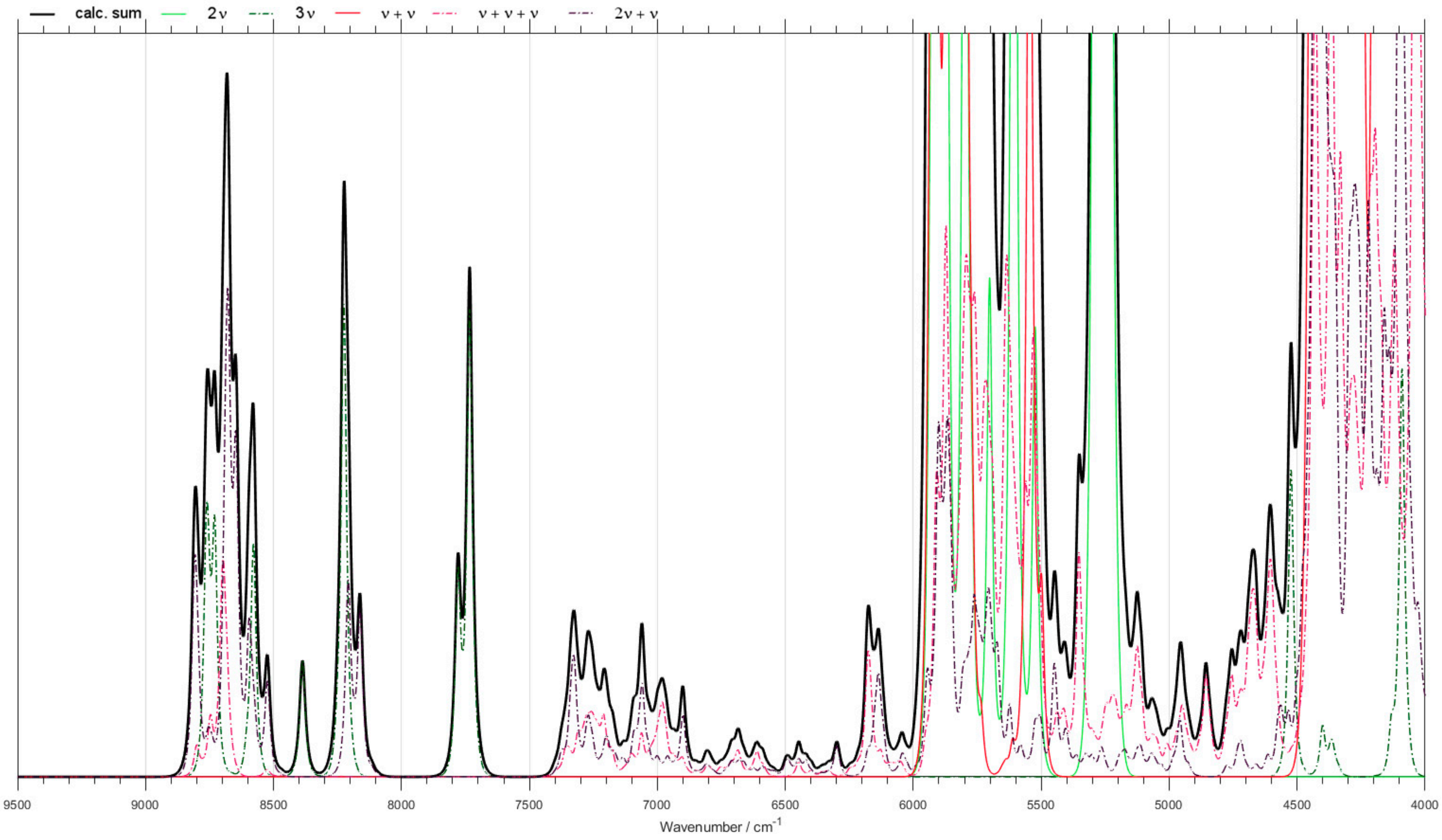

Figure S30. (High-resolution copy of Figure 4) Contributions from minor bands in NIR spectra of CH_3CH_2OD calculated with GVPT2 method at B3LYP-GD3BJ /6-31G(d,p)//CPCM level of electronic theory.

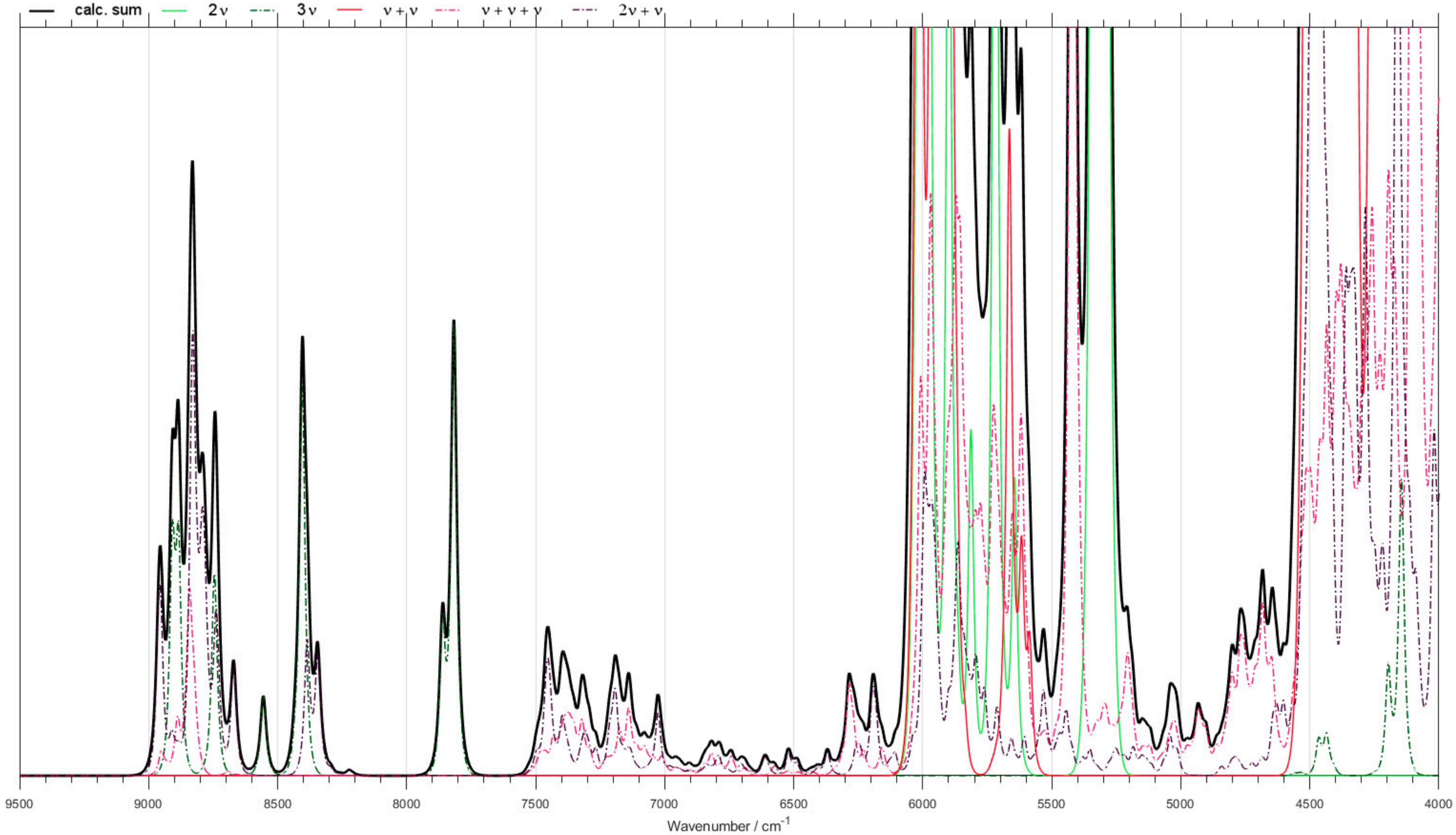

Figure S31. **(High-resolution copy of Figure 4) Contributions from minor bands in NIR spectra of CH_3CH_2OD calculated with GVPT2 method at B2PLYP-GD3BJ/6-31G(d,p)//CPCM level of electronic theory.**

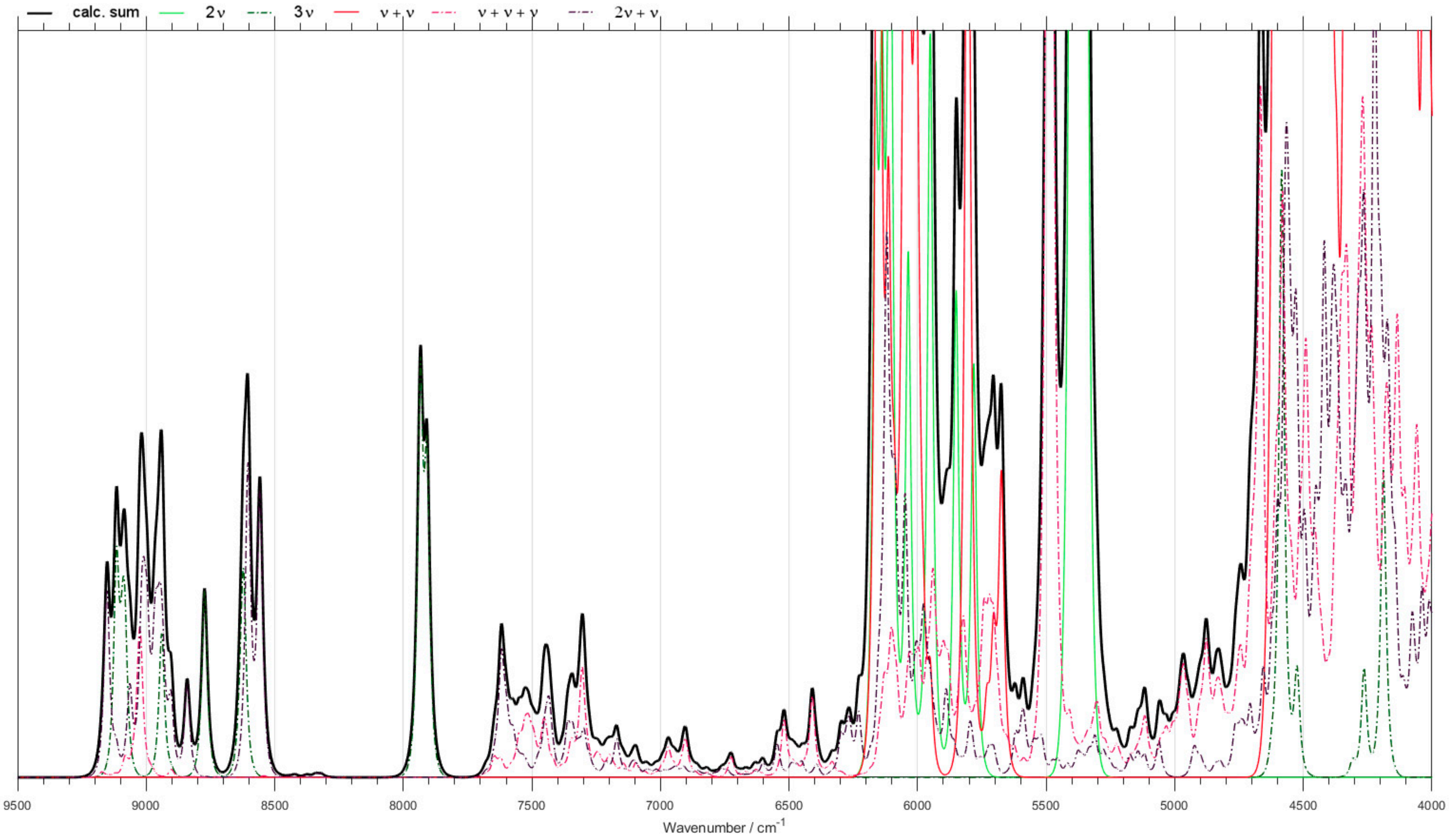

Figure S32. (High-resolution copy of Figure 4) Contributions from minor bands in NIR spectra of CH_3CH_2OD calculated with GVPT2 method at MP2/6-31G(d,p)//CPCM level of electronic theory.

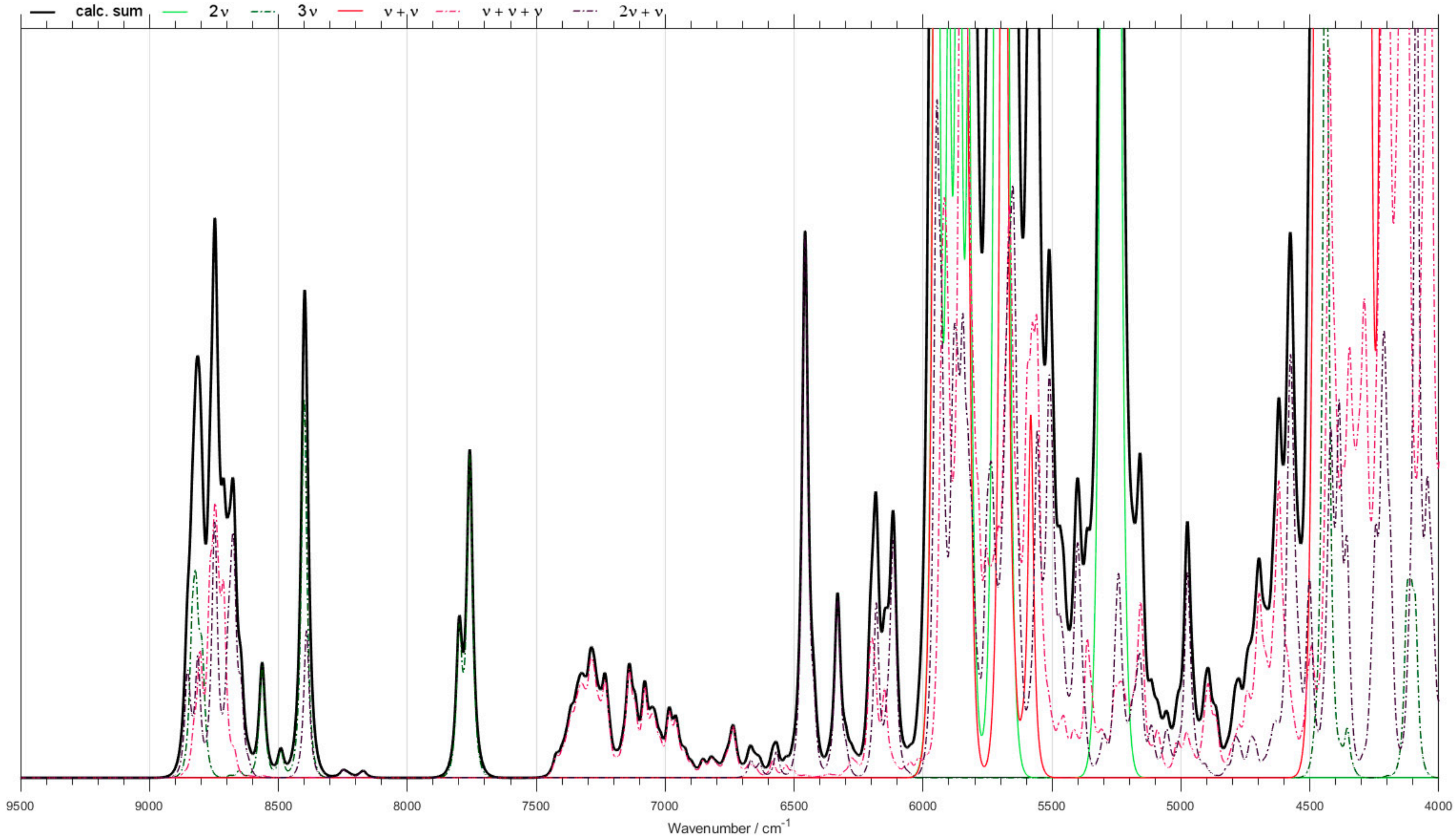

Figure S33. (High-resolution copy of Figure 4) Contributions from minor bands in NIR spectra of CH_3CH_2OD calculated with GVPT2 method at B3LYP-GD3BJ/SNST//CPCM level of electronic theory.

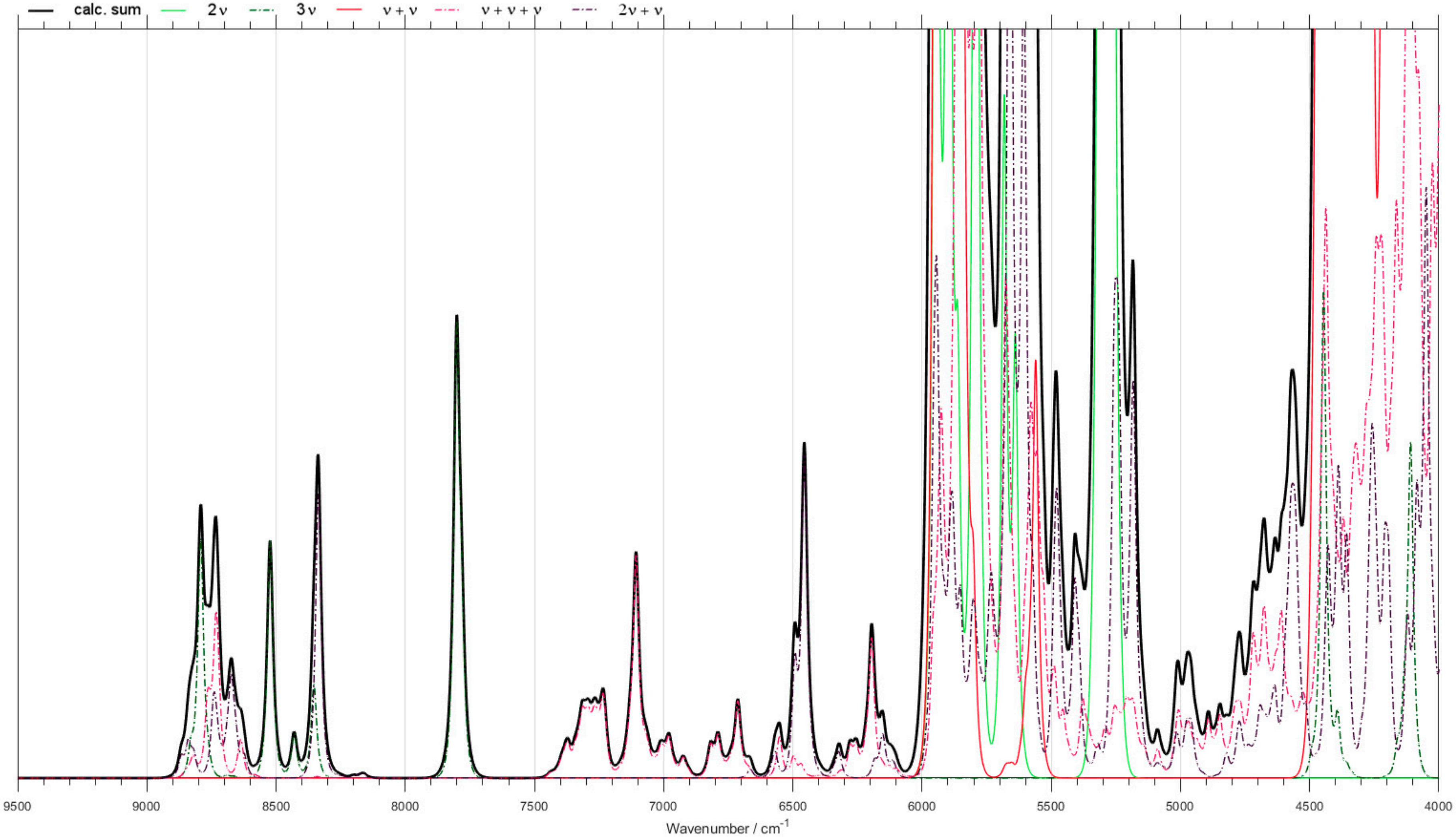

Figure S34. **(High-resolution copy of Figure 4) Contributions from minor bands in NIR spectra of CH_3CH_2OD calculated with GVPT2 method at B2PLYP-GD3BJ/def2-TZVP//CPCM level of electronic theory.**

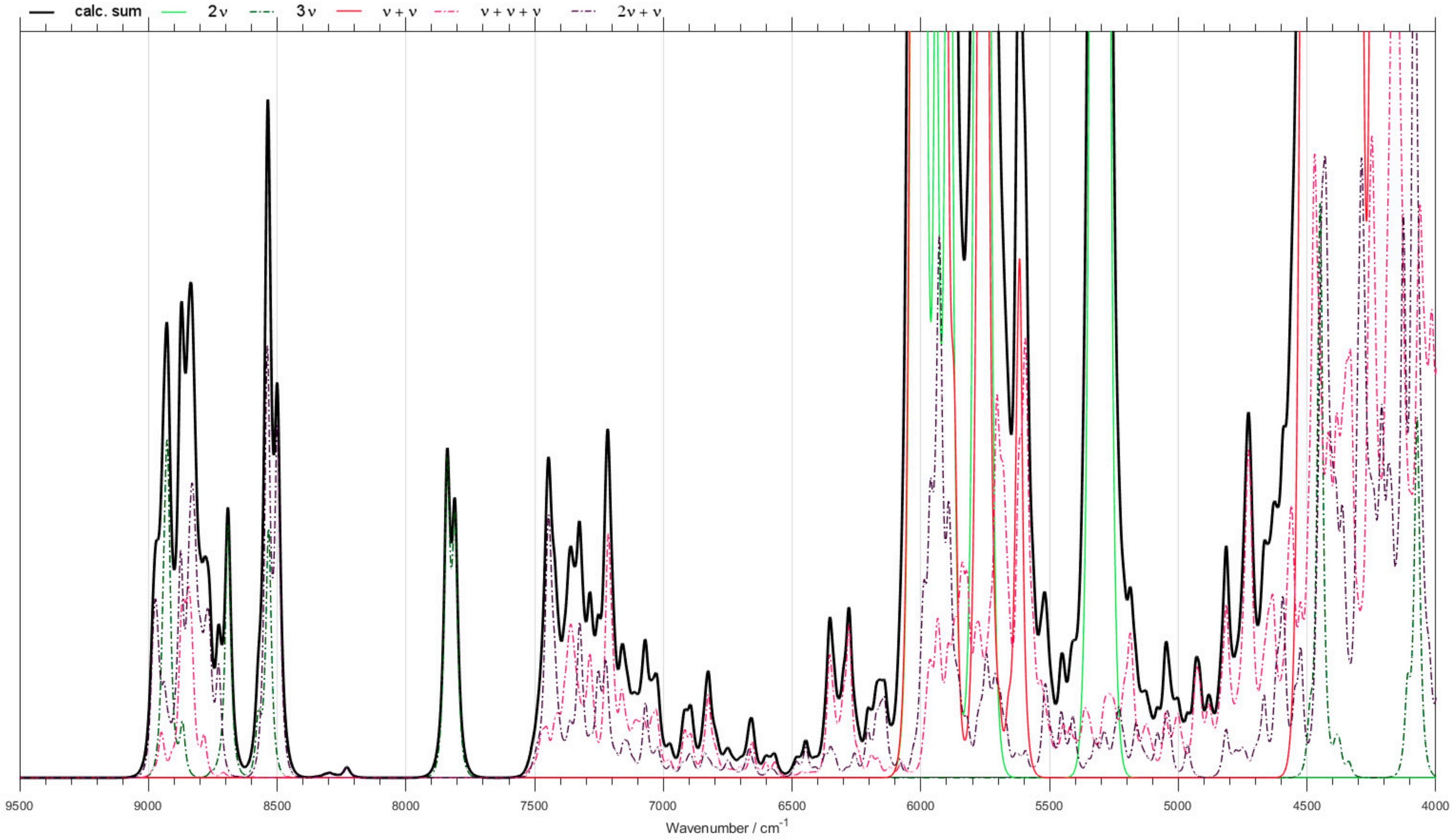

Figure S35. (High-resolution copy of Figure 4) Contributions from minor bands in NIR spectra of CH_3CH_2OD calculated with GVPT2 method at MP2/aug-cc-pVTZ//CPCM level of electronic theory.

molecules

MDPI

II. Tables

Table S1. The relative abundances of gauche (g) and trans (t) conformers for ethanol isotopomers calculated by different methods.

	Conf.	Calculated abundance						
		MP2 /aVTZ +CPCM	B2PLYP-GD3BJ /def2-TZVP +CPCM	B2PLYP-GD3BJ /SNST +CPCM	MP2 /6-31G(d,p) +CPCM	B2PLYP-GD3BJ /6-31G(d,p) +CPCM	B3LYP-GD3BJ /6-31G(d,p) +CPCM	B3LYP-GD3BJ /6-31G(d,p)
CH_3CH_2OH	*g*	45%	37%	82%	40%	87%	74%	79%
	t	55%	63%	18%	60%	13%	26%	21%
CH_3CH_2OD	*g*	48%	24%	73%	46%	78%	76%	79%
	t	52%	76%	27%	54%	22%	24%	21%
CH_3CD_2OH	*g*	47%	38%	83%	44%	87%	74%	79%
	t	53%	62%	17%	56%	13%	26%	21%
CD_3CH_2OH	*g*	60%	51%	83%	52%	88%	73%	79%
	t	40%	49%	17%	48%	12%	27%	21%
CD_3CD_2OH	*g*	58%	51%	84%	54%	87%	73%	79%
	t	42%	49%	16%	46%	13%	27%	21%
CD_3CD_2OD	*g*	51%	37%	69%	53%	80%	77%	79%
	t	49%	63%	31%	47%	20%	23%	21%
CH_3CD_2OD	*g*	48%	24%	73%	46%	79%	76%	79%
	t	52%	76%	27%	54%	21%	24%	21%
CD_3CH_2OD	*g*	50%	35%	70%	52%	80%	77%	79%
	t	50%	65%	30%	48%	20%	23%	21%

Table S2. Potential energy distribution (PED) for *trans* and *gauche* conformers of ethanol (CH_3CH_2OH) based on B2PLYP-GD3BJ/def2-TZVP//CPCM calculations.

trans

ν [cm^{-1}]	%	Int. coord.	%	Int. coord.	%	Int. coord.	%	Int. coord.	%	Int. coord.
240.08	**53%**	τCC	**45%**	δ_{oop}COH						
282.43	**53%**	δ_{oop}COH	**42%**	τCC						
420.77	**78%**	$\delta_{sciss}CH_2CO$	**12%**	$\delta_{rock'}CH_3$						
826.43	**40%**	$\delta_{rock}CH_2$	**33%**	$\delta_{rock}CH_3$	**13%**	$\delta_{twist}CH_2$	**11%**	$\delta_{rock'}CH_3$		
904.94	**38%**	νC-O	**24%**	νCC	**21%**	$\delta_{rock'}CH_3$	**7%**	$\delta_{rock}CH_3$	**6%**	$\delta_{wagg}CH_2$
1045.00	**49%**	νCC	**21%**	δ_{ip}COH	**14%**	νC-O	**8%**	$\delta_{rock'}CH_3$		
1105.80	**54%**	νC-O	**22%**	$\delta_{rock'}CH_3$	**8%**	$\delta_{rock}CH_3$	**5%**	$\delta_{sciss}CH_2CO$		

1188.46	**55%**	$\delta_{rock}CH_2$	**21%**	$\delta_{rock}CH_3$	**11%**	$\delta_{twist}CH_2$	**7%**	$\delta_{rock'}CH_3$		
1272.73	**58%**	$\delta_{ip}COH$	**18%**	$\delta_{wagg}CH_2$	**7%**	$\delta_{rock'}CH_3$				
1310.73	**85%**	$\delta_{twist}CH_2$	**9%**	$\delta_{rock}CH_3$						
1414.01	**85%**	$\delta_{s}CH_3$	**10%**	$\delta_{wagg}CH_2$						
1462.36	**60%**	$\delta_{wagg}CH_2$	**14%**	$\delta_{ip}COH$	**12%**	νCC	**8%**	$\delta_{s}CH_3$		
1492.43	**68%**	$\delta_{as}CH_3$	**23%**	$\delta_{as'}CH_3$	**6%**	$\delta_{rock}CH_3$				
1508.12	**58%**	$\delta_{as'}CH_3$	**19%**	$\delta_{as}CH_3$	**10%**	$\delta_{sciss}CH_2$				
1541.60	**87%**	$\delta_{sciss}CH_2$								
3016.45	**100%**	$\nu_{s}CH_2$								
3047.82	**98%**	$\nu_{as}CH_2$								
3058.32	**99%**	$\nu_{s}CH_3$								
3132.50	**100%**	$\nu_{as}CH_3$								
3134.94	**98%**	$\nu_{as'}CH_3$								
3821.81	**100%**	νOH								

gauche

ν [cm^{-1}]	%	Int. coord.	%	Int. coord.	%	Int. coord.	%	Int. coord.	%	Int. coord.
267.71	**89%**	τCC	**8%**	$\delta_{oop}COH$						
292.31	**84%**	$\delta_{oop}COH$	**9%**	τCC						
425.65	**76%**	$\delta_{sciss}CH_2CO$	**14%**	$\delta_{rock'}CH_3$						
814.37	**44%**	$\delta_{rock}CH_2$	**31%**	$\delta_{rock}CH_3$	**11%**	$\delta_{twist}CH_2$	**9%**	$\delta_{rock'}CH_3$		
892.97	**33%**	$\nu C\text{-}O$	**30%**	νCC	**23%**	$\delta_{rock'}CH_3$	**6%**	$\delta_{rock}CH_3$		
1072.09	**33%**	$\delta_{ip}COH$	**25%**	$\delta_{rock}CH_3$	**9%**	$\delta_{rock}CH_2$	**8%**	$\nu C\text{-}O$	**7%**	$\delta_{rock'}CH_3$
	**7%**	$\delta_{twist}CH_2$								
1076.14	**52%**	$\nu C\text{-}O$	**45%**	νCC						
1142.84	**36%**	$\delta_{rock'}CH_3$	**21%**	$\delta_{rock}CH_2$	**12%**	$\nu C\text{-}O$	**9%**	$\delta_{sciss}CH_2CO$	**7%**	$\delta_{twist}CH_2$
1291.84	**38%**	$\delta_{twist}CH_2$	**22%**	$\delta_{ip}COH$	**18%**	$\delta_{rock}CH_3$	**12%**	$\delta_{rock}CH_2$		
1381.13	**37%**	$\delta_{twist}CH_2$	**28%**	$\delta_{ip}COH$	**22%**	$\delta_{wagg}CH_2$				
1412.78	**72%**	$\delta_{s}CH_3$	**17%**	$\delta_{wagg}CH_2$						
1429.26	**54%**	$\delta_{wagg}CH_2$	**21%**	$\delta_{s}CH_3$	**10%**	νCC	**6%**	$\delta_{ip}COH$		
1497.42	**62%**	$\delta_{as}CH_3$	**27%**	$\delta_{as'}CH_3$	**7%**	$\delta_{rock}CH_3$				
1501.59	**50%**	$\delta_{as'}CH_3$	**24%**	$\delta_{as}CH_3$	**17%**	$\delta_{sciss}CH_2$				
1530.40	**82%**	$\delta_{sciss}CH_2$	**9%**	$\delta_{as'}CH_3$						
3027.04	**99%**	$\nu_{s}CH_2$								
3046.71	**99%**	$\nu_{s}CH_3$								
3100.59	**57%**	$\nu_{as}CH_2$	**39%**	$\nu_{as}CH_3$						
3120.42	**69%**	$\nu_{as'}CH_3$	**28%**	$\nu_{as}CH_2$						
3132.07	**90%**	$\nu_{as}CH_3$	**8%**	$\nu_{as}CH_2$						
3808.92	**100%**	νOH								

Table S3. Potential energy distribution (PED) for *trans* and *gauche* conformers of ethanol-d_1 (CH_3CH_2OD) based on B2PLYP-GD3BJ/def2-TZVP//CPCM calculations.

trans

ν [cm^{-1}]	%	Int. coord.	%	Int. coord.	%	Int. coord.	%	Int. coord.	%	Int. coord.
190.45	**88%**	$\delta_{oop}COD$	**11%**	τCC						
263.60	**91%**	τCC								
411.13	**77%**	$\delta_{sciss}CH_2CO$	**11%**	$\delta_{rock'}CH_3$						
826.34	**40%**	$\delta_{rock}CH_2$	**33%**	$\delta_{rock}CH_3$	**13%**	$\delta_{twist}CH_2$	**11%**	$\delta_{rock'}CH_3$		
863.78	**60%**	$\delta_{ip}COD$	**32%**	νCC	**7%**	νC-O				
914.47	**30%**	νC-O	**25%**	$\delta_{rock'}CH_3$	**22%**	$\delta_{ip}COD$	**9%**	$\delta_{wagg}CH_2$	**8%**	$\delta_{rock}CH_3$
1085.01	**55%**	νC-O	**39%**	νCC						
1167.15	**27%**	$\delta_{rock'}CH_3$	**15%**	$\delta_{ip}COD$	**14%**	$\delta_{sciss}CH_2CO$	**14%**	νCC	**13%**	νC-O
	**9%**	$\delta_{rock}CH_3$								
1188.46	**55%**	$\delta_{rock}CH_2$	**21%**	$\delta_{rock}CH_3$	**11%**	$\delta_{twist}CH_2$	**7%**	$\delta_{rock'}CH_3$		
1310.71	**85%**	$\delta_{twist}CH_2$	**9%**	$\delta_{rock}CH_3$						
1412.18	**73%**	$\delta_{s}CH_3$	**23%**	$\delta_{wagg}CH_2$						
1441.73	**60%**	$\delta_{wagg}CH_2$	**19%**	$\delta_{s}CH_3$	**13%**	νCC				
1492.41	**68%**	$\delta_{as}CH_3$	**23%**	$\delta_{as'}CH_3$	**6%**	$\delta_{rock}CH_3$				
1507.94	**59%**	$\delta_{as}'CH_3$	**19%**	$\delta_{as}CH_3$	**10%**	$\delta_{sciss}CH_2$				
1541.60	**87%**	$\delta_{sciss}CH_2$								
2782.51	**100%**	νOD								
3016.41	**100%**	$\nu_{s}CH_2$								
3047.82	**98%**	$\nu_{as}CH_2$								
3058.32	**99%**	$\nu_{s}CH_3$								
3132.50	**100%**	$\nu_{as}CH_3$								
3134.94	**98%**	$\nu_{as'}CH_3$								

gauche

ν [cm^{-1}]	%	Int. coord.	%	Int. coord.	%	Int. coord.	%	Int. coord.	%	Int. coord.	%	Int. coord.
214.16	**94%**	$\delta_{oop}COD$										
269.87	**90%**	τCC										
418.35	**76%**	$\delta_{sciss}CH_2CO$	**14%**	$\delta_{rock'}CH_3$								
802.16	**46%**	$\delta_{rock}CH_2$	**21%**	$\delta_{rock}CH_3$	**11%**	$\delta_{ip}COD$	**11%**	$\delta_{rock'}CH_3$	**7%**	$\delta_{twist}CH_2$		
890.32	**23%**	νC-O	**22%**	$\delta_{rock}CH_3$	**18%**	νCC	**17%**	$\delta_{rock'}CH_3$	**11%**	$\delta_{ip}COD$	**6%**	$\delta_{wagg}CH_2$
898.75	**52%**	$\delta_{ip}COD$	**13%**	νCC	**11%**	νC-O	**9%**	$\delta_{twist}CH_2$	**8%**	$\delta_{rock}CH_3$	**5%**	$\delta_{rock'}CH_3$
1075.02	**54%**	νC-O	**42%**	νCC								
1129.02	**44%**	$\delta_{rock'}CH_3$	**16%**	νC-O	**15%**	$\delta_{sciss}CH_2CO$	**10%**	νCC				
1227.78	**46%**	$\delta_{rock}CH_2$	**30%**	$\delta_{rock}CH_3$	**13%**	$\delta_{ip}COD$						
1336.60	**90%**	$\delta_{twist}CH_2$										
1408.02	**49%**	$\delta_{wagg}CH_2$	**47%**	dsCH3								

1424.48	**42%**	dsCH3	**40%**	$\delta_{wagg}CH_2$	**11%**	νCC						
1496.19	**61%**	$\delta_{as}CH_3$	**29%**	$\delta_{as'}CH_3$	**7%**	$\delta_{rock}CH_3$						
1501.56	**48%**	$\delta_{as'}CH_3$	**26%**	$\delta_{as}CH_3$	**16%**	$\delta_{sciss}CH_2$						
1530.30	**82%**	$\delta_{sciss}CH_2$	**9%**	$\delta_{as'}CH_3$								
2772.31	**100%**	νOD										
3027.09	**99%**	$\nu_s CH_2$										
3046.76	**99%**	$\nu_s CH_3$										
3100.95	**56%**	$\nu_{as}CH_2$	**41%**	$\nu_{as}CH_3$								
3120.52	**69%**	$\nu_{as'}CH_3$	**29%**	$\nu_{as}CH_2$								
3132.08	**90%**	$\nu_{as}CH_3$	**8%**	$\nu_{as}CH_2$								

Table S4. Potential energy distribution (PED) for *trans* and *gauche* conformers of ethanol-*d*$_2$ (CH_3CD_2OH) based on B2PLYP-GD3BJ/def2-TZVP//CPCM calculations.

trans

ν [cm^{-1}]	%	Int. coord.	%	Int. coord.	%	Int. coord.	%	Int. coord.	%	Int. coord.
238.36	**57%**	τCC	**41%**	δ_{oop} COH						
279.21	**59%**	δ_{oop} COH	**37%**	τCC						
415.53	**78%**	$\delta_{sciss}CD_2CO$	**11%**	$\delta_{rock'}CH_3$						
692.46	**48%**	$\delta_{rock}CD_2$	**23%**	δ_{twist} CD_2	**20%**	$\delta_{rock}CH_3$	**7%**	$\delta_{rock'}CH_3$		
868.09	**31%**	νCC	**26%**	$\delta_{wagg}CD_2$	**17%**	νC-O	**16%**	$\delta_{rock'}CH_3$	**6%**	$\delta_{rock}CH_3$
929.51	**71%**	δ_{twist} CD_2	**26%**	$\delta_{rock}CD_2$						
976.18	**66%**	νC-O	**30%**	$\delta_{wagg}CD_2$						
1024.92	**26%**	νCC	**18%**	$\delta_{rock'}CH_3$	**16%**	δ_{ip} COH	**16%**	$\delta_{sciss}CD_2$	**6%**	$\delta_{sciss}CD_2CO$
	**6%**	$\delta_{rock}CH_3$	**6%**	νC-O						
1130.99	**60%**	$\delta_{sciss}CD_2$	**15%**	νCC	**13%**	δ_{ip} COH	**7%**	$\delta_{wagg}CD_2$		
1152.61	**50%**	$\delta_{rock}CH_3$	**17%**	$\delta_{rock}CD_2$	**16%**	$\delta_{rock'}CH_3$	**9%**	δ_{twist} CD_2	**6%**	dasCH3
1191.98	**28%**	νC-O	**23%**	$\delta_{wagg}CD_2$	**18%**	$\delta_{rock'}CH_3$	**15%**	$\delta_{sciss}CD_2$	**6%**	$\delta_{rock}CH_3$
1339.86	**61%**	$\delta_{ip}COH$	**15%**	$\delta_{wagg}CD_2$	**11%**	νCC	**8%**	dsCH3		
1423.43	**83%**	$\delta_s CH_3$	**12%**	νCC						
1491.00	**69%**	$\delta_{as}CH_3$	**23%**	$\delta_{as'}CH_3$	**6%**	$\delta_{rock}CH_3$				
1509.03	**68%**	$\delta_{as'}CH_3$	**22%**	$\delta_{as}CH_3$	**6%**	$\delta_{rock'}CH_3$				
2192.04	**99%**	$\nu_s CD_2$								
2266.52	**98%**	$\nu_{as}CD_2$								
3058.29	**100%**	$\nu_s CH_3$								
3132.15	**100%**	$\nu_{as}CH_3$								
3133.16	**100%**	$\nu_{as'}CH_3$								
3821.79	**100%**	νOH								

gauche

ν [cm^{-1}]	%	Int. coord.	%	Int. coord.	%	Int. coord.	%	Int. coord.	%	Int. coord.
264.47	**94%**	τCC								

289.31	**91%**	δ_{oop}COH								
420.04	**76%**	$\delta_{sciss}CD_2CO$	**13%**	$\delta_{rock'}CH_3$						
683.50	**53%**	$\delta_{rock}CD_2$	**19%**	$\delta_{rock}CH_3$	**18%**	$\delta_{twist}CD_2$	**6%**	$\delta_{rock'}CH_3$		
858.34	**37%**	νCC	**22%**	$\delta_{wagg}CD_2$	**17%**	$\delta_{rock'}CH_3$	**15%**	νC-O		
904.18	**62%**	δ_{ip}COH	**17%**	$\delta_{rock}CD_2$	**14%**	δ_{ip}COH				
979.09	**67%**	νC-O	**24%**	$\delta_{wagg}CD_2$						
1047.93	**38%**	$\delta_{sciss}CD_2$	**21%**	$\delta_{rock'}CH_3$	**14%**	νCC	**10%**	$\delta_{sciss}CD_2CO$	**10%**	$\delta_{rock}CH_3$
1137.78	**41%**	$\delta_{rock}CH_3$	**22%**	$\delta_{sciss}CD_2$	**13%**	δ_{ip}COH	**7%**	δ_{ip}COH	**5%**	$\delta_{rock}CD_2$
1155.43	**45%**	$\delta_{sciss}CD_2$	**18%**	νCC	**16%**	$\delta_{rock'}CH_3$	**6%**	$\delta_{rock}CD_2$		
1193.50	**43%**	$\delta_{wagg}CD_2$	**28%**	νC-O	**13%**	νCC	**10%**	$\delta_{rock'}CH_3$		
1322.11	**83%**	δ_{ip}COH								
1417.05	**90%**	$\delta_{s}CH_3$	**8%**	νCC						
1495.37	**71%**	$\delta_{as}CH_3$	**20%**	$\delta_{as'}CH_3$	**6%**	$\delta_{rock}CH_3$				
1504.32	**70%**	$\delta_{as'}CH_3$	**21%**	$\delta_{as}CH_3$	**5%**	$\delta_{rock'}CH_3$				
2208.01	**98%**	$\nu_{s}CD_2$								
2305.19	**98%**	$\nu_{as}CD_2$								
3046.67	**99%**	$\nu_{s}CH_3$								
3113.45	**95%**	$\nu_{as'}CH_3$								
3129.50	**96%**	$\nu_{as}CH_3$								
3808.77	**100%**	νOH								

Table S5. Potential energy distribution (PED) for *trans* and *gauche* conformers of ethanol-d_3 (CD_3CH_2OH) based on B2PLYP-GD3BJ/def2-TZVP//CPCM calculations.

trans

ν [cm^{-1}]	%	Int. coord.	%	Int. coord.	%	Int. coord.	%	Int. coord.	%	Int. coord.
192.63	**85%**	τCC	**10%**	$\delta_{oop}COH$						
266.42	**95%**	$\delta_{oop}COH$								
376.32	**70%**	$\delta_{sciss}CH_2CO$	**18%**	$\delta_{rock'}CD_3$	**6%**	$\delta_{rock}CD_3$				
683.12	**48%**	$\delta_{rock}CD_3$	**23%**	$\delta_{rock}CH_2$	**16%**	$\delta_{rock'}CD_3$	**9%**	$\delta_{twist}CH_2$		
770.55	**40%**	$\delta_{rock'}CD_3$	**19%**	νC-O	**14%**	νCC	**14%**	$\delta_{rock}CD_3$		
934.38	**34%**	νCC	**31%**	$\delta_{s}CD_3$	**10%**	$\delta_{sciss}CH_2CO$	**10%**	$\delta_{ip}COH$	**9%**	$\delta_{rock'}CD_3$
1061.99	**63%**	νC-O	**17%**	$\delta_{as'}CD_3$	**6%**	$\delta_{sciss}CH_2CO$	**6%**	$\delta_{as}CD_3$		
1072.32	**72%**	$\delta_{as}CD_3$	**24%**	$\delta_{as'}CD_3$						
1090.74	**54%**	$\delta_{as'}CD_3$	**18%**	$\delta_{as}CD_3$	**16%**	νC-O	**5%**	$\delta_{rock'}CD_3$		
1125.48	**79%**	$\delta_{rock}CH_2$	**15%**	$\delta_{rock}CD_3$						
1154.80	**48%**	$\delta_{s}CD_3$	**33%**	νCC	**11%**	$\delta_{ip}COH$	**6%**	νC-O		
1257.45	**59%**	$\delta_{ip}COH$	**25%**	$\delta_{twist}CH_2$	**6%**	νCC				
1284.71	**96%**	$\delta_{twist}CH_2$								
1459.15	**72%**	$\delta_{wagg}CH_2$	**15%**	$\delta_{ip}COH$	**10%**	νCC				
1538.68	**95%**	$\delta_{sciss}CH_2$								
2197.73	**99%**	$\nu_{s}CD_3$								
2320.27	**98%**	$\nu_{as}CD_3$								
2322.02	**98%**	$\nu_{as'}CD_3$								
3016.88	**100%**	$\nu_{s}CH_2$								
3049.95	**100%**	$\nu_{as}CH_2$								
3821.81	**100%**	νOH								

gauche

ν [cm^{-1}]	%	Int. coord.	%	Int. coord.	%	Int. coord.	%	Int. coord.	%	Int. coord.
204.58	**94%**	τCC								
287.82	**89%**	$\delta_{oop}COH$								
382.13	**66%**	$\delta_{sciss}CH_2CO$	**21%**	$\delta_{rock'}CD_3$						
677.65	**49%**	$\delta_{rock}CD_3$	**25%**	$\delta_{rock}CH_2$	**13%**	$\delta_{rock'}CD_3$	**9%**	$\delta_{twist}CH_2$		
761.11	**40%**	$\delta_{rock'}CD_3$	**20%**	νCC	**16%**	νC-O	**12%**	$\delta_{rock}CD_3$	**6%**	$\delta_{s}CD_3$
950.19	**33%**	$\delta_{s}CD_3$	**28%**	νCC	**13%**	$\delta_{sciss}CH_2CO$	**11%**	$\delta_{rock'}CD_3$	**6%**	$\delta_{rock}CD_3$
	**6%**	$\delta_{ip}COH$								
1038.09	**42%**	νC-O	**21%**	$\delta_{ip}COH$	**20%**	$\delta_{rock}CH_2$	**6%**	$\delta_{rock}CD_3$	**6%**	$\delta_{s}CD_3$
1073.56	**78%**	$\delta_{as}CD_3$	**11%**	$\delta_{rock}CH_2$	**7%**	νC-O				
1076.18	**69%**	$\delta_{as'}CD_3$	**9%**	$\delta_{rock}CH_2$	**7%**	νC-O	**5%**	$\delta_{rock}CD_3$		
1092.43	**28%**	νC-O	**25%**	$\delta_{as'}CD_3$	**15%**	$\delta_{as}CD_3$	**14%**	$\delta_{rock}CH_2$	**13%**	$\delta_{rock'}CD_3$
1163.57	**50%**	$\delta_{s}CD_3$	**44%**	νCC						
1247.02	**55%**	$\delta_{twist}CH_2$	**30%**	$\delta_{ip}COH$	**7%**	$\delta_{rock}CH_2$	**5%**	$\delta_{rock'}CD_3$		
1378.71	**33%**	$\delta_{twist}CH_2$	**29%**	$\delta_{ip}COH$	**27%**	$\delta_{wagg}CH_2$	**5%**	$\delta_{rock}CH_2$		

1425.30	**73%**	$\delta_{wagg}CH_2$	**13%**	$\delta_{ip}COH$	**6%**	νCC				
1526.06	**95%**	$\delta_{sciss}CH_2$								
2189.67	**98%**	$\nu_s CD_3$								
2306.01	**96%**	$\nu_{as'}CD_3$								
2318.96	**99%**	$\nu_{as}CD_3$								
3027.99	**99%**	$\nu_s CH_2$								
3109.63	**100%**	$\nu_{as}CH_2$								
3808.90	**100%**	νOH								

Table S6. Potential energy distribution (PED) for *trans* and *gauche* conformers of ethanol-*d*5 (CD_3CD_2OH) based on B2PLYP-GD3BJ/def2-TZVP//CPCM calculations.

trans

ν [cm^{-1}]	%	Int. coord.	%	Int. coord.	%	Int. coord.	%	Int. coord.	%	Int. coord.
187.52	**87%**	τCC	**9%**	$\delta_{oop}COH$						
265.38	**96%**	$\delta_{oop}COH$								
372.06	**70%**	$\delta_{sciss}CD_2CO$	**17%**	$\delta_{rock'}CD_3$	**6%**	$\delta_{rock}CD_3$				
601.73	**35%**	$\delta_{rock}CD_2$	**35%**	$\delta_{rock}CD_3$	**16%**	$\delta_{twist}CD_2$	**11%**	$\delta_{rock'}CD_3$		
753.55	**34%**	$\delta_{rock'}CD_3$	**18%**	νCC	**14%**	$\nu C\text{-}O$	**13%**	$\delta_{wagg}CD_2$	**12%**	$\delta_{rock}CD_3$
	**5%**	$\delta_s CD_3$								
903.34	**28%**	νCC	**19%**	$\delta_s CD_3$	**15%**	$\delta_{rock'}CD_3$	**12%**	$\delta_{sciss}CD_2CO$	**8%**	$\delta_{sciss}CD_2$
	**6%**	$\delta_{ip}COH$	**5%**	$\delta_{rock}CD_3$						
928.30	**71%**	$\delta_{twist}CD_2$	**25%**	$\delta_{rock}CD_2$						
976.91	**52%**	$\nu C\text{-}O$	**33%**	$\delta_{wagg}CD_2$	**6%**	$\delta_{sciss}CD_2CO$				
1005.34	**38%**	$\delta_{rock}CD_2$	**33%**	$\delta_{rock}CD_3$	**17%**	$\delta_{twist}CD_2$	**11%**	$\delta_{rock'}CD_3$		
1074.89	**72%**	$\delta_{as}CD_3$	**24%**	$\delta_{as'}CD_3$						
1083.27	**66%**	$\delta_{as'}CD_3$	**22%**	$\delta_{as}CD_3$	**6%**	$\delta_{sciss}CD_2$				
1091.17	**27%**	$\delta_s CD_3$	**25%**	$\delta_{wagg}CD_2$	**18%**	$\delta_{sciss}CD_2$	**14%**	$\nu C\text{-}O$		
1154.70	**53%**	$\delta_{sciss}CD_2$	**16%**	$\delta_s CD_3$	**15%**	νCC	**6%**	$\nu C\text{-}O$	**5%**	$\delta_{ip}COH$
1163.43	**23%**	$\nu C\text{-}O$	**17%**	$\delta_s CD_3$	**16%**	$\delta_{ip}COH$	**16%**	νCC	**14%**	$\delta_{wagg}CD_2$
	**8%**	$\delta_{sciss}CD_2$								
1348.97	**55%**	$\delta_{ip}COH$	**21%**	νCC	**17%**	$\delta_{wagg}CD_2$				
2189.80	**80%**	$\nu_s CD_2$	**20%**	$\nu_s CD_3$						
2199.60	**79%**	$\nu_s CD_3$	**18%**	$\nu_s CD_2$						
2265.09	**96%**	$\nu_{as}CD_2$								
2320.38	**98%**	$\nu_{as}CD_3$								
2323.35	**96%**	$\nu_{as'}CD_3$								
3821.79	**100%**	νOH								

gauche

ν [cm^{-1}]	%	Int. coord.	%	Int. coord.	%	Int. coord.	%	Int. coord.	%	Int. coord.
198.93	**96%**	τCC								

286.11	**91%**	$\delta_{oop}COH$								
377.64	**66%**	$\delta_{sciss}CD_2CO$	**20%**	$\delta_{rock'}CD_3$						
595.93	**38%**	$\delta_{rock}CD_2$	**35%**	$\delta_{rock}CD_3$	**15%**	$\delta_{twist}CD_2$	**10%**	$\delta_{rock'}CD_3$		
744.86	**34%**	$\delta_{rock'}CD_3$	**24%**	νCC	**11%**	$\nu C\text{-}O$	**11%**	dwaggCH2	**11%**	$\delta_{rock}CD_3$
	**6%**	$\delta_{s}CD_3$								
894.42	**38%**	$\delta_{twist}CD_2$	**24%**	$\delta_{rock}CD_2$	**15%**	$\delta_{ip}COH$	**9%**	$\delta_{rock}CD_3$		
918.78	**17%**	νCC	**16%**	$\delta_{rock'}CD_3$	**14%**	$\delta_{s}CD_3$	**11%**	$\delta_{twist}CD_2$	**11%**	$\delta_{sciss}CD_2CO$
	**8%**	$\delta_{sciss}CD_2$	**7%**	$\nu C\text{-}O$	**6%**	$\delta_{rock}CD_2$	**5%**	d$\delta_{wagg}CD_2$		
981.92	**48%**	$\nu C\text{-}O$	**18%**	$\delta_{wagg}CD_2$	**12%**	$\delta_{twist}CD_2$	**7%**	$\delta_{sciss}CD_2CO$		
1000.98	**29%**	$\delta_{twist}CD_2$	**25%**	$\delta_{rock}CD_3$	**22%**	$\delta_{rock}CD_2$	**12%**	$\delta_{rock'}CD_3$	**5%**	$\nu C\text{-}O$
1076.06	**65%**	$\delta_{as}CD_3$	**31%**	$\delta_{as'}CD_3$						
1080.46	**58%**	$\delta_{as'}CD_3$	**29%**	$\delta_{as}CD_3$	**7%**	$\delta_{sciss}CD_2$				
1097.28	**42%**	$\delta_{s}CD_3$	**23%**	$\delta_{wagg}CD_2$	**18%**	$\delta_{sciss}CD_2$	**7%**	$\nu C\text{-}O$	**6%**	$\delta_{as'}CD_3$
1143.62	**57%**	$\delta_{sciss}CD_2$	**19%**	$\nu C\text{-}O$	**7%**	$\delta_{wagg}CD_2$	**5%**	$\delta_{rock'}CD_3$		
1206.44	**39%**	νCC	**28%**	$\delta_{wagg}CD_2$	**16%**	$\delta_{s}CD_3$	**14%**	$\nu C\text{-}O$		
1319.55	**86%**	$\delta_{ip}COH$								
2189.05	**96%**	$\nu_{s}CD_3$								
2208.19	**95%**	$\nu_{s}CD_2$								
2298.37	**55%**	$\nu_{as}CD_2$	**42%**	$\nu_{as}CD_3$						
2310.52	**67%**	$\nu_{as'}CD_3$	**29%**	$\nu_{as}CD_2$						
2321.35	**86%**	$\nu_{as}CD_3$	**9%**	$\nu_{as}CD_2$						
3808.76	**100%**	νOH								

Table S7. Potential energy distribution (PED) for *trans* and *gauche* conformers of ethanol-*d*$_6$ (CD_3CD_2OD) based on B2PLYP-GD3BJ/def2-TZVP//CPCM calculations.

trans

ν [cm^{-1}]	%	Int. coord.	%	Int. coord.	%	Int. coord.	%	Int. coord.	%	Int. coord.
179.69	**53%**	τCC	**45%**	$\delta_{oop}COD$						
204.12	**52%**	$\delta_{oop}COD$	**44%**	τCC						
364.00	**70%**	$\delta_{sciss}CD_2CO$	**17%**	$\delta_{rock'}CD_3$	**6%**	$\delta_{rock}CD_3$				
601.58	**35%**	$\delta_{rock}CD_2$	**35%**	$\delta_{rock}CD_3$	**16%**	$\delta_{twist}CD_2$	**11%**	$\delta_{rock'}CD_3$		
751.58	**35%**	$\delta_{rock'}CD_3$	**15%**	νCC	**14%**	$\delta_{wagg}CD_2$	**13%**	$\nu C\text{-}O$	**12%**	$\delta_{rock}CD_3$
828.13	**56%**	$\delta_{ip}COD$	**21%**	νCC	**7%**	$\delta_{s}CD_3$				
928.30	**71%**	$\delta_{twist}CD_2$	**25%**	$\delta_{rock}CD_2$						
929.81	**27%**	$\nu C\text{-}O$	**27%**	$\delta_{wagg}CD_2$	**11%**	$\delta_{s}CD_3$	**10%**	νCC	**8%**	$\delta_{rock'}CD_3$
	**5%**	$\delta_{ip}COD$								
1005.34	**38%**	$\delta_{rock}CD_2$	**33%**	$\delta_{rock}CD_3$	**17%**	$\delta_{twist}CD_2$	**11%**	$\delta_{rock'}CD_3$		
1040.69	**37%**	$\nu C\text{-}O$	**22%**	$\delta_{ip}COD$	**19%**	$\delta_{sciss}CD_2$	**12%**	$\delta_{sciss}CD_2CO$		
1074.86	**72%**	$\delta_{as}CD_3$	**24%**	$\delta_{as'}CD_3$						
1083.27	**67%**	$\delta_{as'}CD_3$	**22%**	$\delta_{as}CD_3$	**6%**	$\delta_{sciss}CD_2$				
1104.25	**60%**	$\delta_{s}CD_3$	**21%**	$\delta_{wagg}CD_2$	**5%**	$\delta_{sciss}CD_2$				

1157.79	**55%**	$\delta_{sciss}CD_2$	**24%**	νC-O	**10%**	$\delta_{wagg}CD_2$	**5%**	$\delta_{rock'}CD_3$		
1256.52	**44%**	νCC	**28%**	$\delta_{wagg}CD_2$	**11%**	$\delta_{s}CD_3$	**7%**	δ_{ip}COD		
2189.63	**80%**	$\nu_{s}CD_2$	**17%**	$\nu_{s}CD_3$						
2199.52	**79%**	$\nu_{s}CD_3$	**18%**	$\nu_{s}CD_2$						
2265.08	**96%**	$\nu_{as}CD_2$								
2320.37	**98%**	$\nu_{as}CD_3$								
2323.35	**96%**	$\nu_{as'}CD_3$								
2782.50	**100%**	νOD								

gauche

ν [cm^{-1}]	%	Int. coord.	%	Int. coord.	%	Int. coord.	%	Int. coord.	%	Int. coord.
193.34	**79%**	τCC	**18%**	δ_{oop}COD						
215.82	**73%**	δ_{oop}COD	**20%**	τCC						
371.21	**68%**	$\delta_{sciss}CD_2CO$	**20%**	$\delta_{rock'}CD_3$						
593.24	**39%**	$\delta_{rock}CD_2$	**32%**	$\delta_{rock}CD_3$	**14%**	$\delta_{twist}CD_2$	**11%**	$\delta_{rock'}CD_3$		
739.08	**32%**	$\delta_{rock'}CD_3$	**19%**	νCC	**17%**	$\delta_{rock}CD_3$	**11%**	$\delta_{wagg}CD_2$	**10%**	νC-O
812.42	**47%**	δ_{ip}COD	**21%**	$\delta_{twist}CD_2$	**12%**	$\delta_{rock}CD_2$	**8%**	νCC	**6%**	$\delta_{rock}CD_3$
918.23	**19%**	νCC	**17%**	$\delta_{rock'}CD_3$	**15%**	$\delta_{s}CD_3$	**11%**	$\delta_{sciss}CD_2CO$	**9%**	$\delta_{sciss}CD_2$
	**8%**	$\delta_{twist}CD_2$	**7%**	νC-O	**6%**	$\delta_{wagg}CD_2$				
980.31	**44%**	νC-O	**19%**	$\delta_{twist}CD_2$	**17%**	$\delta_{wagg}CD_2$	**6%**	$\delta_{sciss}CD_2CO$	**5%**	$\delta_{rock}CD_3$
995.22	**40%**	$\delta_{twist}CD_2$	**15%**	$\delta_{rock}CD_3$	**13%**	$\delta_{rock'}CD_3$	**9%**	$\delta_{rock}CD_2$	**8%**	νC-O
1064.16	**33%**	$\delta_{as}CD_3$	**22%**	δ_{ip}COD	**20%**	$\delta_{rock}CD_2$	**13%**	$\delta_{sciss}CD_2$		
1078.79	**75%**	$\delta_{as'}CD_3$	**10%**	$\delta_{sciss}CD_2$	**5%**	$\delta_{s}CD_3$				
1084.38	**62%**	$\delta_{as}CD_3$	**12%**	$\delta_{as'}CD_3$	**7%**	δ_{ip}COD				
1102.03	**47%**	$\delta_{s}CD_3$	**21%**	$\delta_{wagg}CD_2$	**8%**	nC-O	**7%**	δ_{ip}COD		
1154.46	**56%**	$\delta_{sciss}CD_2$	**22%**	νC-O	**9%**	$\delta_{wagg}CD_2$				
1211.74	**41%**	νCC	**26%**	$\delta_{wagg}CD_2$	**15%**	$\delta_{s}CD_3$	**11%**	νC-O		
2189.05	**96%**	$\nu_{s}CD_3$								
2208.15	**94%**	$\nu_{s}CD_2$								
2298.13	**56%**	$\nu_{as}CD_2$	**40%**	$\nu_{as}CD_3$						
2310.31	**69%**	$\nu_{as'}CD_3$	**28%**	$\nu_{as}CD_2$						
2321.26	**86%**	$\nu_{as}CD_3$	**9%**	$\nu_{as}CD_2$						
2772.85	**100%**	νOD								

Table S8. Potential energy distribution (PED) for *trans* and *gauche* conformers of CH_3CD_2OD based on B2PLYP-GD3BJ/def2-TZVP//CPCM calculations.

trans

ν [cm^{-1}]	%	Int. coord.	%	Int. coord.	%	Int. coord.	%	Int. coord.	%	Int. coord.
190.00	**89%**	$\delta_{oop}COD$	**11%**	τCC						
258.77	**92%**	τCC								
406.06	**77%**	$\delta_{sciss}CD_2CO$	**11%**	$\delta_{rock'}CH_3$						
692.37	**48%**	$\delta_{rock}CD_2$	**23%**	$\delta_{twist}CD_2$	**20%**	$\delta_{rock}CH_3$	**7%**	$\delta_{rock'}CH_3$		
852.44	**68%**	$\delta_{ip}COD$	**14%**	$\delta_{wagg}CD_2$	**10%**	νCC				
867.18	**30%**	νCC	**26%**	$\delta_{wagg}CD_2$	**18%**	$\nu C\text{-}O$	**16%**	$\delta_{rock'}CH_3$	**6%**	$\delta_{rock}CH_3$
929.51	**71%**	$\delta_{twist}CD_2$	**26%**	$\delta_{rock}CD_2$						
988.62	**61%**	$\nu C\text{-}O$	**12%**	νCC	**10%**	$\delta_{ip}COD$	**9%**	$\delta_{wagg}CD_2$	**5%**	$\delta_{rock'}CH_3$
1083.26	**61%**	$\delta_{sciss}CD_2$	**13%**	$\delta_{rock'}CH_3$	**11%**	$\delta_{sciss}CD_2CO$	**7%**	$\delta_{ip}COD$		
1152.60	**50%**	$\delta_{rock}CH_3$	**17%**	$\delta_{rock}CD_2$	**16%**	$\delta_{rock'}CH_3$	**9%**	$\delta_{twist}CD_2$	**6%**	$\delta_{as}CH_3$
1187.81	**32%**	$\delta_{sciss}CD_2$	**23%**	$\nu C\text{-}O$	**19%**	$\delta_{rock'}CH_3$	**10%**	$\delta_{wagg}CD_2$	**7%**	$\delta_{rock}CH_3$
1233.96	**42%**	$\delta_{wagg}CD_2$	**33%**	νCC	**10%**	$\delta_{ip}COD$	**7%**	$\nu C\text{-}O$	**5%**	$\delta_{sciss}CD_2$
1420.81	**88%**	$\delta_{s}CH_3$	**10%**	νCC						
1490.98	**69%**	$\delta_{as}CH_3$	**23%**	$\delta_{as'}CH_3$	**6%**	$\delta_{rock}CH_3$				
1508.99	**68%**	$\delta_{as'}CH_3$	**22%**	$\delta_{as}CH_3$	**6%**	$\delta_{rock'}CH_3$				
2191.79	**98%**	$\nu_{s}CD_2$								
2266.51	**98%**	$\nu_{as}CD_2$								
2782.50	**100%**	νOD								
3058.30	**100%**	$\nu_{s}CH_3$								
3132.15	**100%**	$\nu_{as}CH_3$								
3133.16	**100%**	$\nu_{as'}CH_3$								

gauche

ν [cm^{-1}]	%	Int. coord.	%	Int. coord.	%	Int. coord.	%	Int. coord.	%	Int. coord.
212.69	**96%**	$\delta_{oop}COD$								
264.92	**93%**	τCC								
412.62	**76%**	$\delta_{sciss}CD_{2}2CO$	**13%**	$\delta_{rock'}CH_3$						
675.84	**56%**	$\delta_{rock}CD_2$	**16%**	$\delta_{rock}CH_3$	**14%**	$\delta_{twist}CD_2$	**7%**	$\delta_{rock'}CH_3$		
828.91	**44%**	$\delta_{ip}COD$	**38%**	$\delta_{twist}CD_2$	**11%**	$\delta_{rock}CH_3$				
858.49	**37%**	νCC	**20%**	$\delta_{wagg}CD_2$	**17%**	$\delta_{rock'}CH_3$	**16%**	$\nu C\text{-}O$		
976.66	**69%**	$\nu C\text{-}O$	**25%**	$\delta_{wagg}CD_2$						
1021.81	**28%**	$\delta_{twist}CD_2$	**22%**	$\delta_{ip}COD$	**12%**	νCC	**9%**	$\delta_{rock'}CH_3$	**9%**	$\delta_{rock}CH_3$
	**8%**	$\delta_{rock}CD_2$	**7%**	$\delta_{sciss}CD_2$						
1068.48	**49%**	$\delta_{sciss}CD_2$	**14%**	$\delta_{rock'}CH_3$	**12%**	$\delta_{ip}COD$	**9%**	$\delta_{twist}CD_2$	**8%**	$\delta_{sciss}CD_2CO$
1151.99	**30%**	$\delta_{rock}CH_3$	**19%**	$\delta_{rock'}CH_3$	**15%**	$\delta_{sciss}CD_2$	**14%**	$\delta_{rock}CD_2$	**7%**	νCC
	**5%**	$\delta_{twist}CD_2$	**5%**	$\delta_{as}CH_3$						

1183.07	**37%**	$\delta_{sciss}CD_2$	**18%**	$\delta_{rock}CH_3$	**18%**	νCC	**10%**	δ_{ip}COD		
1193.39	**43%**	$\delta_{wagg}CD_2$	**28%**	νC-O	**13%**	νCC	**10%**	$\delta_{rock'}CH_3$		
1417.04	**90%**	$\delta_{s}CH_3$	**8%**	νCC						
1494.71	**68%**	$\delta_{as}CH_3$	**23%**	$\delta_{as'}CH_3$	**6%**	$\delta_{rock}CH_3$				
1504.11	**67%**	$\delta_{as'}CH_3$	**24%**	$\delta_{as}CH_3$	**5%**	$\delta_{rock'}CH_3$				
2207.97	**98%**	$\nu_{s}CD_2$								
2304.66	**97%**	$\nu_{as}CD_2$								
2772.81	**100%**	νOD								
3046.70	**99%**	$\nu_{s}CH_3$								
3113.44	**95%**	$\nu_{as'}CH_3$								
3129.51	**96%**	$\nu_{as}CH_3$								

Table S9. Potential energy distribution (PED) for *trans* and *gauche* conformers of CD_3CH_2OD based on B2PLYP-GD3BJ/def2-TZVP//CPCM calculations.

trans

ν [cm^{-1}]	%	Int. coord.	%	Int. coord.	%	Int. coord.	%	Int. coord.	%	Int. coord.
181.66	**54%**	δ_{oop}COD	**44%**	τCC						
208.61	**54%**	τCC	**39%**	δ_{oop}COD						
368.12	**70%**	$\delta_{sciss}CH_2CO$	**17%**	$\delta_{rock'}CD_3$	**6%**	$\delta_{rock}CD_3$				
682.97	**48%**	$\delta_{rock}CD_3$	**23%**	$\delta_{rock}CH_2$	**16%**	$\delta_{rock'}CD_3$	**10%**	$\delta_{twist}CH_2$		
769.04	**40%**	$\delta_{rock'}CD_3$	**19%**	νC-O	**14%**	νCC	**14%**	$\delta_{rock}CD_3$		
838.77	**61%**	δ_{ip}COD	**20%**	νCC	**6%**	$\delta_{s}CD_3$	**6%**	$\delta_{rock'}CD_3$		
1011.50	**46%**	$\delta_{s}CD_3$	**20%**	νC-O	**19%**	δ_{ip}COD	**8%**	νCC		
1072.29	**72%**	$\delta_{as}CD_3$	**24%**	$\delta_{as'}CD_3$						
1073.79	**37%**	$\delta_{as'}CD_3$	**31%**	νC-O	**12%**	$\delta_{as}CD_3$	**7%**	δ_{ip}COD	**7%**	$\delta_{sciss}CH_2CO$
1098.25	**38%**	νC-O	**28%**	$\delta_{as'}CD_3$	**10%**	$\delta_{rock'}CD_3$	**9%**	$\delta_{as}CD_3$	**7%**	δ_{ip}COD
1125.47	**79%**	$\delta_{rock}CH_2$	**15%**	$\delta_{rock}CD_3$						
1180.18	**46%**	νCC	**44%**	$\delta_{s}CD_3$						
1284.69	**96%**	$\delta_{twist}CH_2$								
1434.91	**85%**	$\delta_{wagg}CH_2$	**9%**	νCC						
1538.67	**95%**	$\delta_{sciss}CH_2$								
2197.72	**99%**	$\nu_{s}CD_3$								
2320.26	**98%**	$\nu_{as}CD_3$								
2322.02	**98%**	$\nu_{as'}CD_3$								
2782.52	**100%**	νOD								
3016.84	**100%**	$\nu_{s}CH_2$								
3049.94	**100%**	$\nu_{as}CH_2$								

gauche

ν [cm^{-1}]	%	Int. coord.	%	Int. coord.	%	Int. coord.	%	Int. coord.	%	Int. coord.
195.28	**68%**	τCC	**29%**	δ_{oop}COD						
221.20	**62%**	δ_{oop}COD	**29%**	τCC						
375.85	**68%**	$\delta_{sciss}CH_2CO$	**20%**	$\delta_{rock'}CD_3$						
676.57	**47%**	$\delta_{rock}CD_3$	**25%**	drockCH2	**14%**	$\delta_{rock'}CD_3$	**9%**	$\delta_{twist}CH_2$		
755.00	**40%**	$\delta_{rock'}CD_3$	**17%**	$\delta_{rock}CD_3$	**16%**	νCC	**14%**	νC-O		
861.02	**63%**	δ_{ip}COD	**12%**	νCC	**8%**	$\delta_{rock}CH_2$	**6%**	$\delta_{s}CD_3$		
978.67	**32%**	$\delta_{s}CD_3$	**18%**	νCC	**14%**	δ_{ip}COD	**11%**	$\delta_{rock'}CD_3$	**10%**	$\delta_{sciss}CH_2CO$
	**7%**	νC-O	**6%**	$\delta_{rock}CH_2$						
1062.80	**60%**	νC-O	**16%**	$\delta_{as'}CD_3$	**9%**	$\delta_{as}CD_3$	**8%**	$\delta_{sciss}CH_2CO$		
1074.49	**65%**	$\delta_{as}CD_3$	**31%**	$\delta_{as'}CD_3$						
1088.27	**48%**	$\delta_{as'}CD_3$	**22%**	$\delta_{as}CD_3$	**19%**	νC-O	**7%**	$\delta_{rock'}CD_3$		
1157.17	**38%**	$\delta_{rock}CH_2$	**21%**	$\delta_{s}CD_3$	**15%**	νCC	**10%**	$\delta_{rock}CD_3$	**8%**	δ_{ip}COD
1166.86	**35%**	$\delta_{s}CD_3$	**34%**	νCC	**12%**	$\delta_{rock}CH_2$	**6%**	δ_{ip}COD		
1323.98	**90%**	$\delta_{twist}CH_2$								
1414.22	**89%**	$\delta_{wagg}CH_2$	**6%**	νCC						
1526.01	**95%**	$\delta_{sciss}CH_2$								
2189.66	**98%**	$\nu_{as}CD_3$								
2305.98	**96%**	$\nu_{as'}CD_3$								
2318.96	**98%**	$\nu_{as}CD_3$								
2772.36	**100%**	νOD								
3028.05	**100%**	$\nu_{s}CH_2$								
3110.10	**99%**	$\nu_{as}CH_2$								
